# A temporal model of tumor-immune dynamics during the metastatic progression of high-grade serous ovarian cancer

**DOI:** 10.1038/s41698-025-00973-y

**Published:** 2025-06-16

**Authors:** Donagh Egan, Kate Glennon, Ann Treacy, Aurelie Fabre, Janet McCormack, Salisha Hill, Ryan Morrison, Vadim Zhernovkov, Daniel Liebler, Walter Kolch, Donal Brennan

**Affiliations:** 1 Systems Biology Ireland, University College Dublin, School of Medicine, Belfield, Dublin 4, Dublin, Ireland; 2 UCD-Gynaecological Oncology Group, School of Medicine, Mater Misericordiae University Hospital, University College Dublin, Dublin, Ireland; 3 Department of Pathology, Mater Misericordiae University Hospital, Dublin, Ireland; 4 Research Pathology Core Facility, Conway Institute, University College Dublin, Dublin, Ireland; 5 Inotiv Inc, Nashville, Tennessee Ireland; 6 Conway Institute of Biomolecular & Biomedical Research, University College Dublin, Belfield, Dublin 4, Dublin, Ireland

**Keywords:** Cancer microenvironment, Cancer models, Metastasis, Tumour immunology, High-throughput screening

## Abstract

Patients with high-grade serous ovarian cancer (HGSOC) typically present with widespread metastasis, obscuring a temporal understanding of tumor-immune dynamics. To address this, we perform multi-site global proteomics alongside matched immunohistochemistry (IHC) for CD4⁺ and CD8⁺ tumor-infiltrating lymphocytes (TILs) in patient samples. We order the protein expression profiles using an unbiased pseudotime analysis, recapitulating clinical observations of metastatic progression, and providing a framework to explore tumor-immune dynamics from localized to metastatic disease. Metastatic progression correlates with immune cell infiltration, the recruitment of regulatory T cells (Tregs) to counterbalance γδ T cell abundance, and an increased abundance of exhausted CD8⁺ T cells. The accumulation of Tregs at metastatic sites correlates with SNX8 expression, a critical regulator of the STING pathway. In early-stage tumors, keratin-expressing cancer cells recruit Tregs via MHC class II, fostering an inflammatory phenotype with limited IFNγ production and non-clonally expanded T cells. Together, our findings reveal novel mechanisms of immune escape associated with both localized disease and metastatic progression in HGSOC.

## Introduction

High-grade serous ovarian cancer (HGSOC) typically presents at an advanced stage with extensive metastatic spread. This prolonged latency period facilitates extensive tumor-immune interactions, which are instrumental in shaping both tumor heterogeneity and immunogenicity^1,2^. As cancer cells colonize diverse metastatic niches, the tumor-immune relationship evolves, leading to divergent fates among tumors from the same patient^3^. Despite the clinical significance of this process, a temporal understanding of tumor-immune coevolution from the primary tumor to metastatic disease remains elusive.

Tumor-infiltrating lymphocytes (TILs) are a hallmark of immune response and a prognostic marker in HGSOC^4^. To carry out their function, T cells must directly engage with cancer cells, prompting the use of immunohistochemistry (IHC) to map the spatial relationships between cancer and immune cells. This approach has identified three basic tumor-immune phenotypes: (1) the infiltrated phenotype, characterized by TILs within the tumor epithelium; (2) the immune-excluded phenotype, wherein TILs are restricted to stromal regions surrounding the tumor; and (3) the desert immune phenotype, which lacks TILs entirely^5^. These immune phenotypes offer a valuable framework for understanding the immune landscape of HGSOC. However, the mechanisms governing the spatial evolution of TILs during metastatic progression remain largely unexplored.

Conducting longitudinal analyses in clinically relevant HGSOC samples is challenging because patients often present with tumors at multiple sites, obscuring the temporal sequence of metastatic dissemination. However, pseudotime analysis can approximate temporal variation by projecting samples into a unidimensional latent space that quantifies relative disease progression^6^. Although pseudotime analysis is mainly applied to single-cell RNA sequencing (scRNA-seq) data, it originates from earlier applications in microarray data for modeling cancer progression^7^. In this study, we leverage this approach to analyze multi-site biopsies obtained during surgical debulking of HGSOC.

**Fig. 1:**
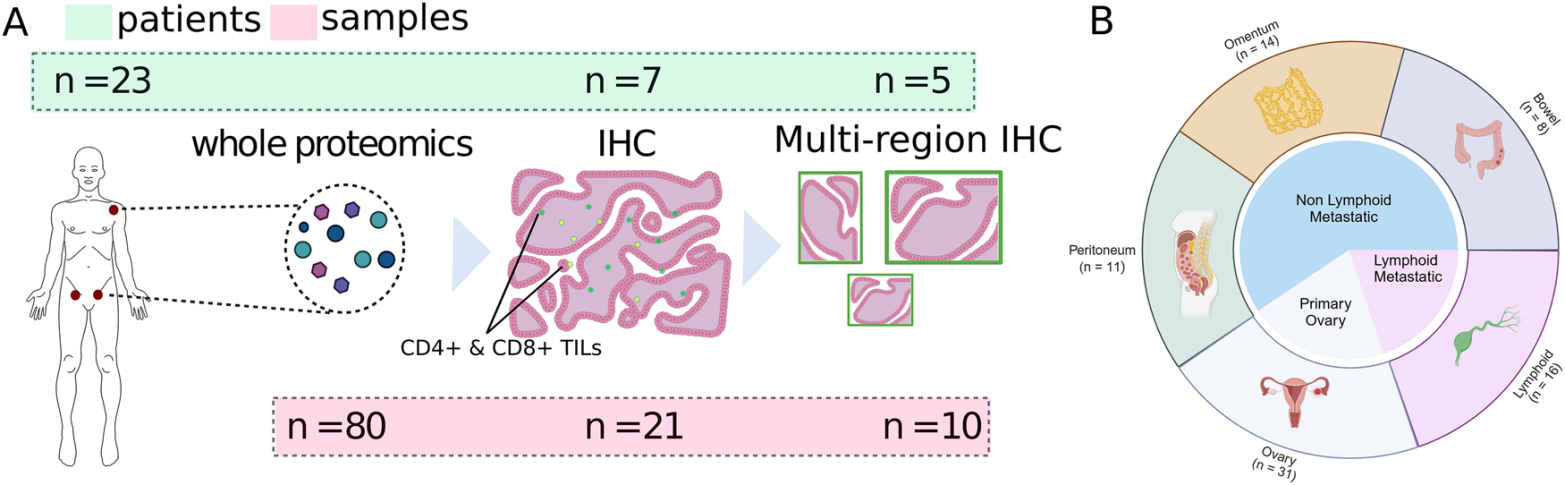
Patient Sampling and Study Design. **A** The methods used for profiling tumors, and number of patients (top box) and samples (bottom box) available for each. **B** The number of samples from each tumor site that underwent global proteomic profiling (Created with Biorender.com).

We performed multi-site global proteomic profiling on 80 HGSOC tumor samples from 23 patients and mapped the spatial distribution of CD4⁺ and CD8⁺ TILs in a subset of samples using IHC (Fig. 1A, B). To investigate intra-tumor heterogeneity (ITH), we performed high-magnification IHC on individual regions from primary ovarian and omental tumors. By integrating each omics into a pseudo-temporal model of metastatic progression, we demonstrate the critical role of Tregs in both primary disease and metastatic spread, uncovering novel mechanisms that drive Treg dynamics and facilitate immune evasion in HGSOC.

## Results

### The recruitment of Tregs correlates with metastatic progression

We conducted global proteomics on Formalin-Fixed Paraffin-Embedded tumor sections (*n* = 23 patients/80 samples) (Supplementary Table 1). In total, 5824 unique proteins were identified (median/sample = 4654), aligning with the coverage reported in prior studies^8^. Furthermore, the number of proteins identified and their dynamic ranges were consistent across tumor sites (Supplementary Fig. 1A, B).

**Fig. 2:**
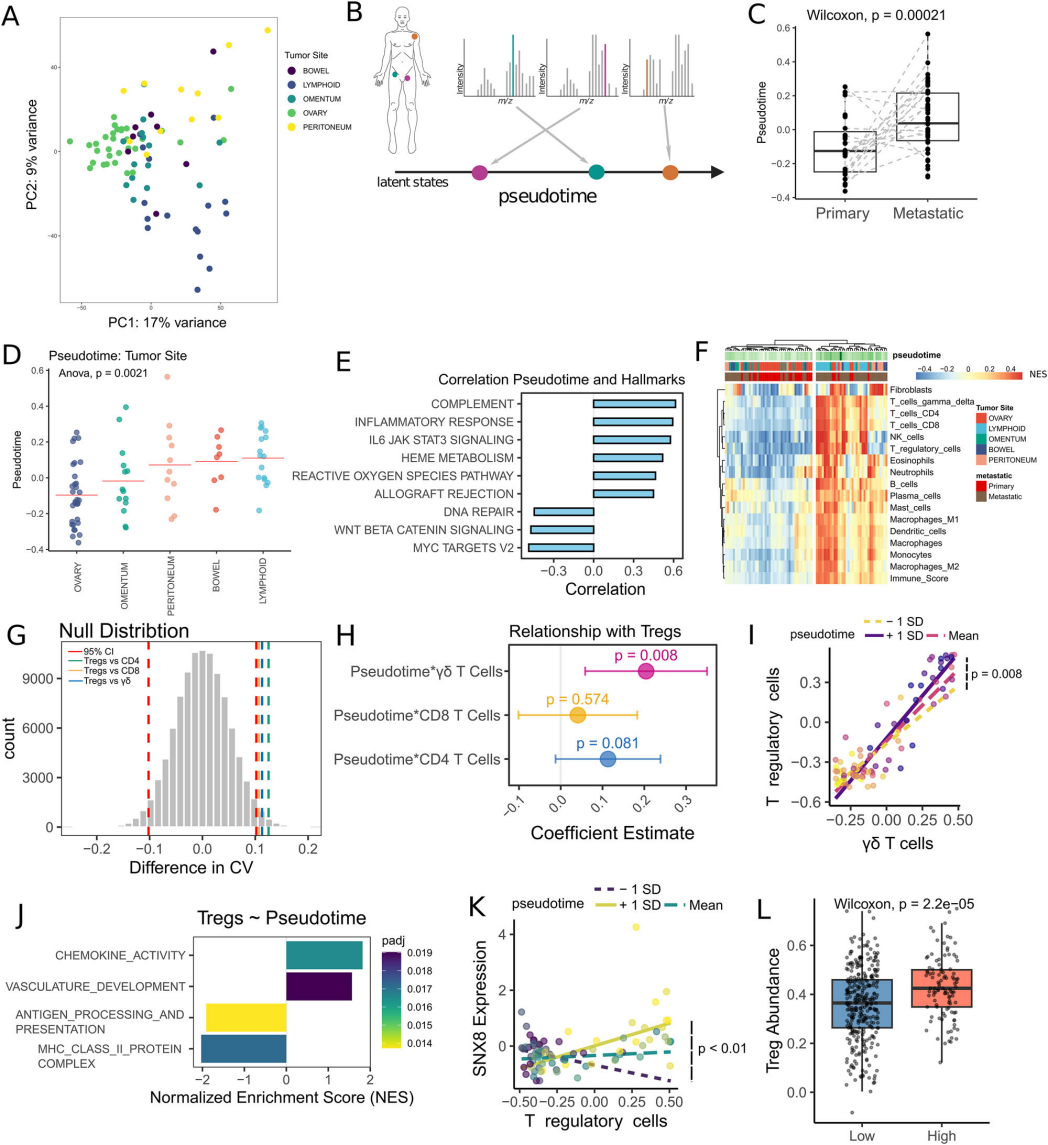
Pseudotime Modeling of Metastatic Progression in HGSOC. **A** Principal component analysis on the proteomic profiles of HGSOC tumor samples, annotated by tumor site. **B** A schematic of the pseudotime analysis in multiple samples from one patient to identify temporal information that traces metastatic progression in HGSOC from a cross-sectional cohort. **C** Pseudotime estimates in primary and metastatic samples. **D** Pseudotime estimates in samples from distinct tumor sites. **E** Spearman’s rho between the pseudotime estimates and pathway activity scores inferred using single-sample GSEA. The hallmark pathways were used, and the top 8 most significant pathways were selected. **F** Hierarchical clustering of HGSOC tumor samples according to deconvolution derived estimates of different cell types. The abundance of each cell type is represented by a normalized enrichment score (NES). **G** A null distribution representing the differences in CV between cell types, estimated using permutations (*n* = 100,000) without replacement. The red lines represent the 95% confidence interval; the remaining lines represent comparisons between cell types. **H** The coefficient estimates for the interaction effects of pseudotime with CD4, CD8, and γδ T cells on Treg abundance. **I** The correlation between the γδ T cells and Tregs, assessed across samples with different pseudotime estimates (−1 standard deviation; mean; and +1 standard deviation). **J** Enriched pathways for genes whose correlation with Tregs changes as a function of pseudotime. A positive NES indicates pathways where the correlation with Tregs increases with pseudotime, while a negative NES reflects pathways where this correlation decreases. **K** The correlation between SNX8 expression and Treg abundance for samples with different pseudotime estimates (−1 standard deviation; mean; and +1 standard deviation). **L** The levels of Tregs in ovarian cancer samples from TCGA, stratified by SNX8 expression.

Principal component analysis identified metastasis status and tumor site as the primary sources of variation, rather than patient identity (Fig. 2A) (Supplementary Fig. 1C). We hypothesized that all samples share a common underlying biological process, with each sample representing a distinct stage in its progression. To investigate this, we performed an unbiased pseudotime analysis using Phenopath^6^, projecting each sample into a continuous, one-dimensional latent space (Fig. 2B). The pseudotime scores increased from primary to metastatic samples (*p* < 0.05) (Fig. 2C), with tumor sites positioned in line with clinical observations of metastatic spread: primary ovarian samples first, followed by the omentum and peritoneum due to direct dissemination within the abdominal cavity, then the bowel via peritoneal spread, and finally lymphoid sites (*n* = 12 retroperitoneal para-aortic; *n* = 4 spleen) (Fig. 2D)^9,10^. Although metastatic progression is shaped by complex inter-patient heterogeneity, the pseudotime analysis aligns with the transcoelomic dissemination of HGSOC - a prevalent route of metastasis^11^ - providing a novel framework to investigate its underlying molecular mechanisms.

To identify pathways involved in metastatic progression, we calculated Spearman’s rho between the pseudotime estimates and pathway activity scores (Hallmarks database), inferred using single-sample GSEA. Immune-related pathways such as the complement system, inflammatory response, and IL6 signaling through JAK and STAT, were the most significant pathways positively correlated with pseudotime (Fig. 2E) (FDR < 0.05). IL-6 produced by omental adipocytes promotes invasion of ovarian cancer cells^12^, whereas the complement system and inflammatory response reflect escalating immune dysfunction, wherein cancer cells hijack macrophages and Tregs to create an immunosuppressive environment^13^. Conversely, DNA repair, MYC targets, and WNT signaling were negatively correlated with pseudotime. Elevated MYC and WNT signaling are associated with immune exclusion in HGOSC^3^, whereas DNA repair defects drive genomic instability and the selection of aggressive clones^14^. To further resolve the molecular mechanisms underlying these associations, we identified genes significantly correlated with pseudotime (FDR < 0.05). Notably, CD44 and ALDH1A1^15^ - established cancer stem cell markers displayed strong positive correlations with pseudotime, reinforcing the established role of a stem-like phenotype in metastatic progression (Supplementary Table 2).

We explored the relationship between tumor composition and metastatic progression by estimating cell type abundances using a deconvolution algorithm^16^. Hierarchical clustering grouped the samples by tumor site, metastatic status, and pseudotime, revealing increased immune cell infiltration at later pseudotime scores (Fig. 2F). Tregs demonstrated the highest coefficient of variation (CV) across the samples (Supplementary Fig. 1D). Given our focus on understanding T cell dynamics during metastatic progression, we compared the variability of effector T cell subsets (CD4⁺, CD8⁺, Tregs, and γδ T cells) and found that Tregs exhibited significantly greater variation, as determined by a permutation-based null distribution (FDR < 0.05) (Fig. 2G).

We hypothesized that the high variability in Treg levels reflects their recruitment to metastatic sites, where they suppress immune responses from effector T cells. To explore this, we analyzed how the relationship between Tregs and each effector T cell phenotype evolved over pseudotime. Notably, the correlation between γδ T cells and Tregs increased significantly with pseudotime (*p* = 0.008), while a near-significant trend was observed for CD4⁺ T cells (*p* = 0.081), and no significant change was seen for CD8⁺ T cells (*p* = 0.57) (Fig. 2H, I). These findings suggest that Treg infiltration at metastatic sites is particularly important for counterbalancing the abundance of γδ T cells.

To investigate the mechanisms underlying Treg recruitment during metastatic progression, we identified genes whose correlation with Tregs changed as a function of pseudotime and mapped these to functional pathways. As pseudotime advanced, we observed a significant increase in the correlation between Tregs and pathways involved in chemokine signaling and vasculature development, but a decrease in the correlation with antigen presentation pathways (FDR < 0.05) (Fig. 2J). This indicates that Treg abundance correlates with antigen presentation in early-stage tumors, and with chemokine-mediated signaling and vascularization at metastatic sites, perhaps reflecting the frequent loss of HLA expression during metastatic progression^17,18^.

From this analysis, the correlation between SNX8 and Tregs increased the most with pseudotime (FDR = 0.003) (Fig. 2K, Supplementary Fig. 1E). SNX8 plays a critical role in the STING pathway^19^, which detects cytosolic DNA resulting from genomic instability and activates downstream non-canonical NF-κB signaling to promote metastasis^20^. This is consistent with the previously observed negative correlation between metastatic progression and DNA repair, a driver of genomic instability (FDR < 0.01) (Fig. 2E)^21^. In The Cancer Genome Atlas (TCGA), ovarian tumors with high SNX8 expression (≥75th percentile) demonstrated significantly increased Treg abundance (*p* < 0.05) (Fig. 2L), further supporting an association between SNX8 expression and Treg infiltration.

### The spatial distribution of TILs changes during metastatic progression

**Fig. 3:**
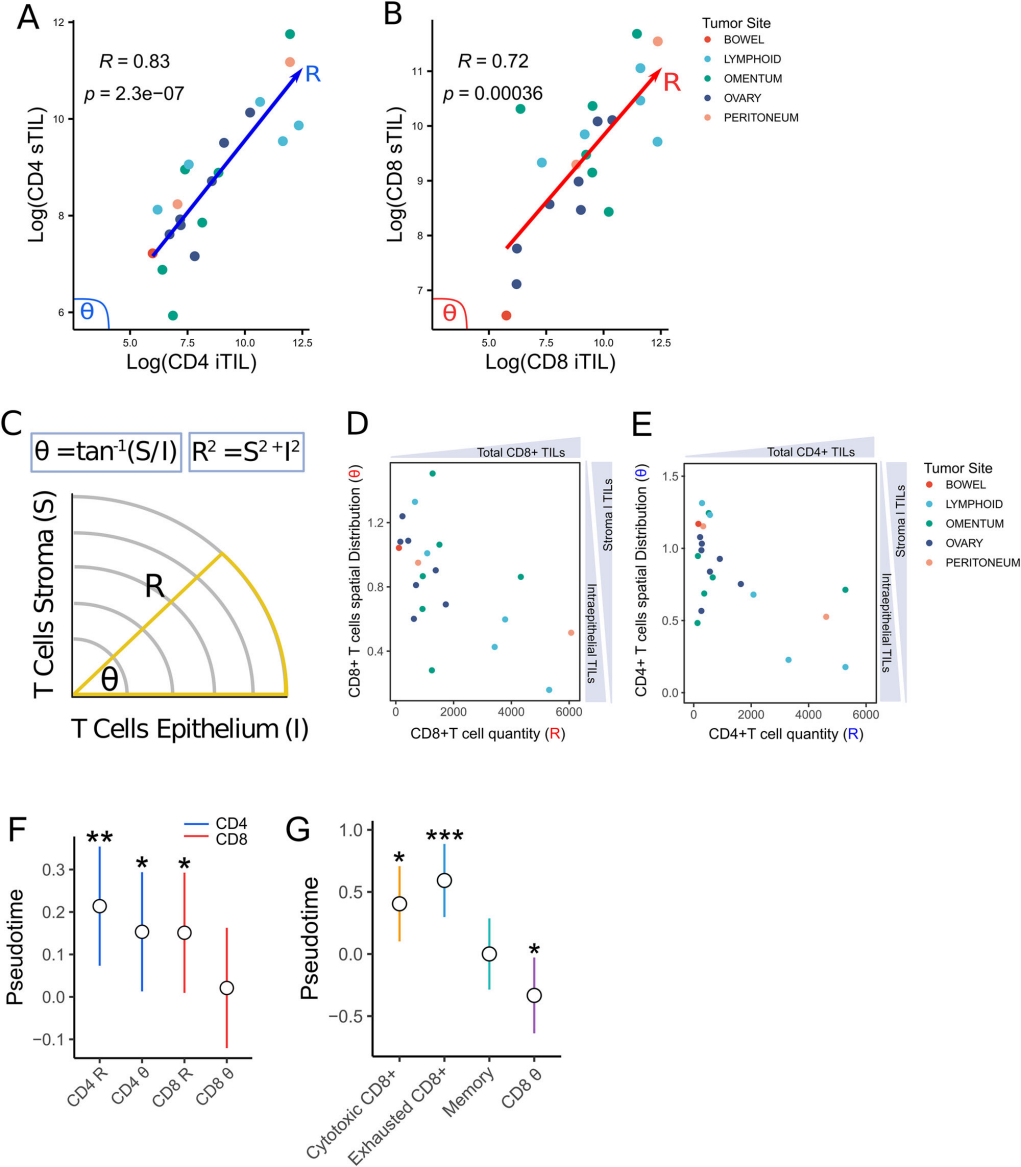
The Spatial Distribution of TILs During Metastatic Progression. **A**, **B** Spearman’s rho between intra-epithelial TILs (iTILs) and stromal TILs (sTILs) for CD4+ and CD8 + T cells, respectively. **C** The method for calculating the polar coordinates. The R value quantifies the overall density of iTILs and sTILs, defined as R = square root [(TILs tumor)^2^ + (TILs stroma)^2^]. θ quantifies the spatial distribution of TILs in the stoma relative to the epithelium, defined as θ = atan(TILs stroma/TILs tumor). **D**, **E** The overall density (R) and spatial distribution (θ) of CD4+ and CD8+ TILs, respectively. **F** Coefficient estimates for the association of the polar coordinates with pseudotime. **G** Coefficient estimates for the association of CD8 + T cell phenotypes and the spatial distribution of CD8+ TILs (θ value) with pseudotime. T cell phenotypes were quantified using gene signatures from a published study^25^. Vertical lines indicate the 5% and 95% confidence intervals (CIs). P-values are denoted as follows: ***p < 0.001; **p < 0.01; *p < 0.05.

We mapped the spatial distribution of CD4+ and CD8+ TILs in intra-epithelial (iTILs) and stromal (sTILs) regions using IHC (*n* = 7 patients/21 samples) (Supplementary Table 3). A positive correlation was observed between the density of iTILs and sTILs for both CD4+ (*R* = 0.83; *p* < 0.01) and CD8+ (*R* = 0.72; *p* < 0.01) TILs (Fig. 3A, B). Therefore, the density of TILs that infiltrate the epithelium is strongly related to the densities observed in the stroma.

To better understand the spatial distribution of TILs we created a new polar coordinate system by converting the densities of CD4+ and CD8+ TILs into two new metrics: (1) The overall density of CD8+ and CD4+ TILs in the epithelium and stromal compartments (R value), and (2) the distribution of CD8+ and CD4+ TILs in the epithelium compared to stromal compartments (θ value) (detailed in methods; adopted from ref. ^22^) (Fig. 3C–E).

We explored the spatial evolution of TILs during metastatic progression by analyzing the relationship between pseudotime and the polar coordinates. Consistent with our previous results, the overall densities of CD8⁺ (*p* = 0.012) and CD4⁺ (*p* = 0.004) TILs positively correlated with pseudotime (R value) (Fig. 3F). Notably, the exclusion of CD4⁺ TILs from the epithelium (*p* < 0.05), but not the exclusion of CD8⁺ TILs, was correlated with pseudotime (Fig. 3F).

scRNA-seq and spatial transcriptomic studies in HGSOC have demonstrated that the spatial distribution of CD8+ TILs varies by phenotype: exhausted T cells infiltrate the tumor epithelium, while pre-exhausted and memory T cells are confined to the stromal compartment^23,24^. Therefore, we hypothesized that differences between CD8⁺ T cell phenotypes might confound the association between the spatial distribution of CD8⁺ TILs (θ value) and pseudotime. Using published gene signatures^25^, we quantified exhausted, cytotoxic, and memory CD8⁺ T cells in the global proteomics data. Once these were controlled for in the model, we observed a positive correlation between CD8 + TIL infiltration into the epithelium and metastatic progression (*p* < 0.05) (Fig. 3G). Furthermore, exhausted CD8⁺ T cells were the strongest positive predictor of pseudotime, suggesting that metastatic progression is accompanied by higher levels of exhaustion (*p* < 0.05) (Fig. 3G).

### CD4+ and CD8 + TILs exhibit distinct patterns of heterogeneity

**Fig. 4:**
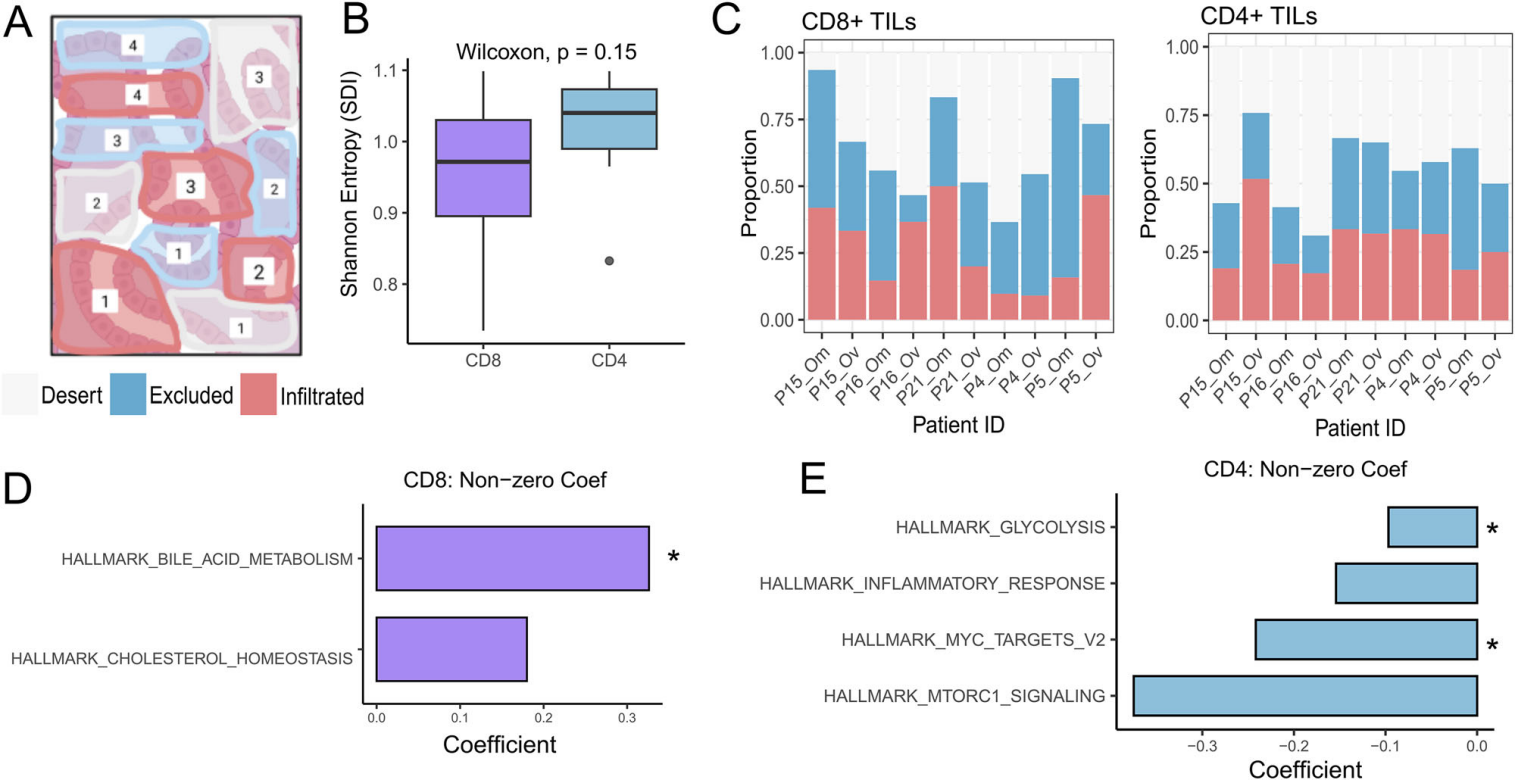
Intra-tumor Heterogeneity of The Immune Phenotypes. **A** An illustrative IHC slide, analyzed at high magnification (x20), where the number of immune, excluded, and infiltrated regions are quantified. **B** The Shannon diversity index (SDI) for the distribution of immune phenotypes in a tumor, assessed for both CD8+ and CD4+ TILs. **C** The distribution of immune phenotypes for CD8+ and CD4+ TILs, respectively. The association between the immune phenotype distributions and tumor samples was assessed using a chi-squared test. **D**, **E** The non-zero coefficients from ridge regression, obtained by modeling pathway activity scores against the SDI values for CD8+ and CD4+ TILs, respectively. Parametric bootstrapping identified coefficient estimates with 95% confidence intervals excluding zero are denoted by *.

In HGSOC, TILs exhibit greater ITH than intra-patient heterogeneity, posing an obstacle for immunotherapy treatments^26^. We investigated ITH in early-stage primary ovarian and omental tumors by analyzing each IHC slide at high magnification (x20), and quantifying the number regions classified as infiltrated, excluded, or desert (illustrative example, Fig. 4A) (Supplementary Table 4). For example, at a low magnification, a full-face section was initially divided into five major regions and classified as excluded due to a higher density of sTILs compared to iTILs. However, when the magnification was increased, 26 individual regions were identified, consisting of infiltrated (*n* = 12), excluded (*n* = 6), and desert (*n* = 16) immune phenotypes (Supplementary Fig. 2A–E).

We used the Shannon diversity index (SDI) to quantify the ITH of immune phenotypes in each sample. In this context, a lower SDI reflects a tumor dominated by a single immune phenotype, while a higher SDI signifies a more balanced distribution of immune phenotypes. CD8+ TILs trended toward a lower SDI compared to CD4+ TILs (*p* = 0.15) (Fig. 4B). Furthermore, the distribution of immune phenotypes showed greater variability across tumor samples for CD8+ TILs compared to CD4+ TILs (*p* < 0.01 and *p* = 0.06, respectively) (Fig. 4C). These findings suggest that within a tumor, a single immune phenotype tends to dominate for CD8+ TILs. However, the dominant phenotype varies significantly across tumors, perhaps reflecting distinct selection pressures exerted on CD4+ and CD8+ TILs.

To understand the selection pressures exerted on CD8+ and CD4 + T cells, we preformed lasso regression with parametric bootstrapping (10,000 replicates) to identify pathways that best explain the variation in ITH. This approach identifies coefficient estimates whose 95% confidence interval does not include zero. We identified bile acid metabolism (95% CI: 0.31–1.64) as a consistent predictor for CD8+ TILs, whereas glycolysis (95% CI: −0.2 to −0.18) and MYC targets (95% CI: −0.19 to −0.18) were predictive for CD4+ TILs. Tumor metabolic constraints modulate T cell phenotypes: under low-glucose conditions, CD8 + T cells demonstrate diminished effector function^27^, whereas CD4 + T cell differentiation is biased toward regulatory T cell formation^28^. Our findings demonstrate that tumor metabolism, specifically bile acid metabolism and glycolysis, also shapes the ITH of CD8⁺ and CD4⁺ TILs, respectively.

### Keratin expressing cancer cells constrain the immune response via Tregs

Having identified mechanisms that drive metastatic progression and ITH in early-stage disease, we analyzed the primary ovarian and omental and samples to elucidate novel tumor-immune interactions that permit localized disease. The densities of CD8+ and CD4+ TILs, determined by IHC analysis, were significantly correlated with deconvolution-derived estimates of CD8+ and CD4 + T cells in the global proteomics dataset (FDR < 0.05) (Supplementary Fig. 3A), highlighting a concordance and mutual robustness of both approaches. Therefore, we categorized each sample as infiltrated or non-infiltrated based on the predominant immune phenotype from the multi-region IHC analysis and identified differentially expressed proteins between both groups.

**Fig. 5:**
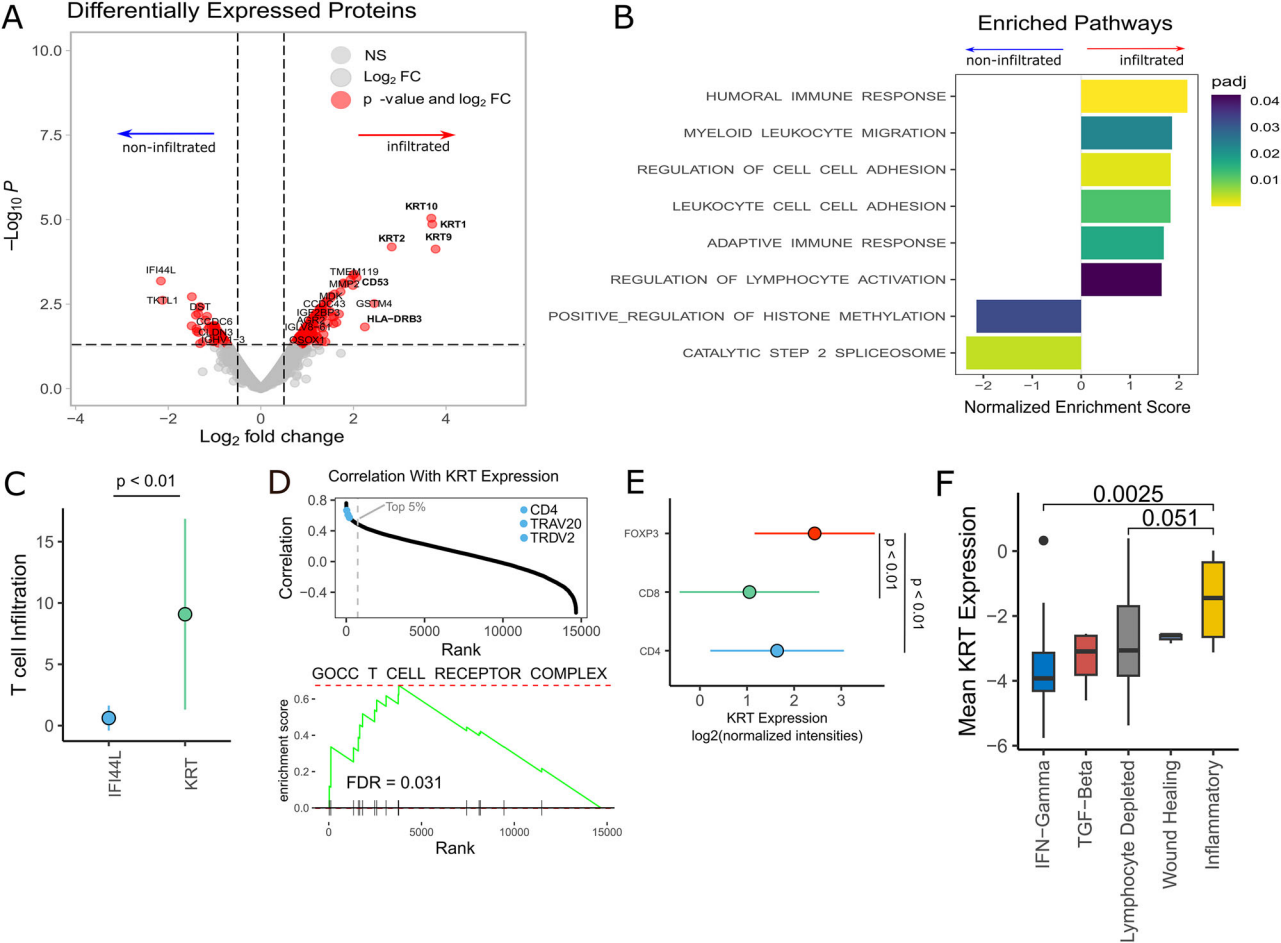
The mechanisms underlying T cell infiltration and exclusion. **A** The differentially expressed proteins between infiltrated and non-infiltrated tumors. The genes delineated in red satisfied the significance (*p* < 0.05) and fold change (log2FC > 1) thresholds. **B** Enriched pathways between infiltrated and non-infiltrated tumors. An FDR threshold of 0.05 was implemented. **C** Coefficient estimates for the association of keratin and IFI44L expression with the fraction of T cells in the published dataset^26^, compared using a t-test. The vertical lines represent the 5% and 95% CIs. **D** Pearson’s correlation coefficient between each gene and keratin expression (top panel). Genes left of the dotted gray line are in the top 5%. GSEA for the T cell receptor complex pathway using genes ranked by their correlation with keratin expression (bottom panel). **E** Coefficient estimates for the association between keratin expression and the fractions of Tregs, CD4 + , and CD8 + T cells in the published dataset^26^, compared using a t-test. The horizontal lines represent the 5% and 95% CIs. **F** The mean keratin protein expression in the independent proteogenomic ovarian cancer dataset^34^. The tumor phenotypes are compared using a Wilcoxon rank sum test.

Keratin proteins (KRT 1, 2, 9, and 10) were the most upregulated proteins in infiltrated tumors, whereas IFI44L was the most upregulated protein in non-infiltrated tumors (Fig. 5A). As expected, immune-related pathways, such as adaptive immune response and lymphocyte activation were enriched in infiltrated tumors. Conversely, the spliceosome pathway was downregulated, potentially enhancing tumor immunogenicity by creating more splicing-derived neoantigens (FDR < 0.05) (Fig. 5B)^29^.

As an initial validation step, we confirmed the expression of keratin in ovarian cancer cells using two independent scRNA-seq datasets, which were integrated with Harmony (Supplementary Fig. 3B–D)^30–32^. We then leveraged a published dataset with multicolor immunofluorescence quantification of CD4 + , CD8 + , and Tregs in treatment-naïve HGSOC samples (*n* = 37)^26^. At least ten tumor sections per sample were analyzed, excluding stromal areas, yielding 440 regions with matched transcriptomic data. In this dataset, we confirmed that keratin expression (KRT1, KRT2, KRT9, and KRT10) is correlated with T cell infiltration (*p* = 0.02), and this association is significantly stronger compared to IFI44L (*p* < 0.01) (Fig. 5C).

To understand keratin-induced T cell infiltration, we identified genes co-expressed with keratin using Pearson’s correlation in the validation dataset, and mapped these to Gene Ontology pathways with GSEA. The co-expressed genes were significantly enriched for the T cell receptor complex pathway, with CD4, TRAV20, and TRDV2 among the top five percent of co-expressed genes (FDR < 0.05) (Fig. 5D). Keratin expressing stromal cells constrain the immune response by presenting self-antigens via MHC class II to Tregs^33^. Given the co-expression of keratin proteins with variable TCR regions and the concomitant upregulation of HLA-DRB3 in infiltrated tumors (Fig. 5A), we hypothesized that ovarian cancer cells retain this mechanism from their epithelial origin. Indeed, keratin expression was the strongest predictor of Treg infiltration in the validation dataset^26^, followed by CD4 + T cells, and then CD8 + T cells in (*p* < 0.01) (Fig. 5E). Furthermore, in ovarian cancer cells from the integrated scRNA-seq dataset, keratin expression was positively correlated with MHC class II activity (*R* = 0.22, *p* < 0.01) (Supplementary Fig. 3E).

Next, we analyzed keratin protein expression in a proteogenomic dataset of ovarian cancer samples (*n* = 82) classified according to phenotypes from the TCGA pan-cancer study^34,35^. Tumors with an inflammatory phenotype upregulated keratin expression compared to lymphocyte-depleted and IFNγ-expressing tumors (*p* = 0.051 and *p* = 0.0025, respectively) (Fig. 5F). This demonstrates that keratin expression is associated with inflammation, infiltrating lymphocytes, but not with interferon gamma production, supporting its role in constraining the immune response through Tregs. Finally, high keratin expression was associated with worse overall survival in ovarian cancer datasets from TCGA and gene expression omnibus (GEO) (*p* < 0.05) (Supplementary Fig. 3F), demonstrating that Treg infiltration due to keratin expression is associated with worse prognosis in HGSOC^36^.

### The proteome underlying keratin expression restricts T cell clonal expansion

Upon antigen-specific T-cell receptor stimulation, Tregs suppress the cytotoxic activity of CD8 + T cells and restrict clonal expansion against both self and foreign antigens^37^. To investigate how the transcriptome underlying elevated keratin expression affects the clonal composition of immune cells, we analyzed data from Zhang et al.^1^, which included matched Nanostring transcriptomics and T- and B-cell receptor sequencing data from 111 samples across 20 HGSOC patients. These samples were classified to a desert, excluded, or infiltrated immune phenotype by the original authors^1^.

**Fig. 6:**
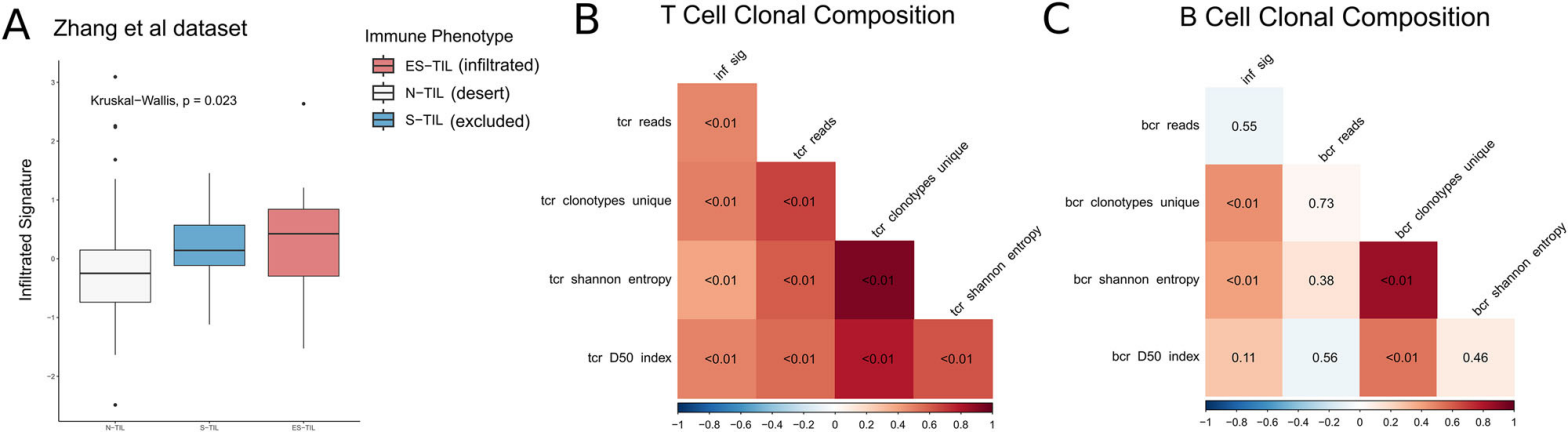
The proteome underlying keratin expression restricts clonal expansion. **A** The activity of proteins identified as upregulated in infiltrated samples (the infiltrated signature) from our dataset, assessed in samples from the Zhang et al.¹ dataset. The samples were classified to an immune phenotype by the original authors. **B** The correlation of the infiltrated signature (inf_sig) with T cell abundance (tcr_reads), T cell diversity (Shannon entropy, D50 index), and the number of unique T cell clones (clonotype unique). The color scale represents the correlation coefficients, and the FDR corrected *p* values are provided in the tiles. **C** The correlation of the infiltrated signature (inf_sig) with B cell abundance (bcr_reads), B cell diversity (Shannon entropy, D50 index), and the number of unique B cell clones (clonotype unique). The color scale represents the correlation coefficients, and the FDR corrected p values are provided in the tiles.

First, we confirmed that the proteins identified as upregulated (logFC > 0, *p* < 0.05) in the infiltrated samples from our dataset, referred to as the “infiltrated signature,” were upregulated in the immune-infiltrated phenotype from the published dataset^1^ (*p* < 0.05) (Fig. 6A). This suggests that the proteomic changes underlying elevated keratin expression are conserved at the transcriptomic level in an independent dataset.

Next, we analyzed the T and B cell receptor sequencing data. Given that individual patients contributed multiple samples, we calculated a partial Spearman’s correlation coefficient between the previously defined “infiltrated signature” and metrics defining the clonal composition of T and B cells. The infiltrated signature positively correlated with the number of TCR reads in a sample, a proxy for T cell infiltration, as well as measures of clonal diversity such as Shannon’s entropy and the D50 index (FDR < 0.05) (Fig. 6B). This suggests that keratin proteins correlate with T cell infiltration but not with clonal expansion (emergence of dominant clone), indicative of Treg-induced immunosuppression.

Tregs are essential and sufficient to suppress autoreactive B cells in an antigen-specific manner^38^. The “infiltrated signature” demonstrated no correlation with B cell infiltration (BCR reads) (FDR > 0.05), but was positively correlated with measures of clonal diversity (FDR < 0.05) (Fig. 6E). Therefore, although the proteome underlying keratin expression does not correlate with B cell infiltration, it coincides with limited clonal expansion, potentially through the action of Tregs.

## Discussion

Immune checkpoint blockade has failed to deliver meaningful responses in HGSOC, despite being considered an immunogenic disease^39,40^. To improve patient outcomes, a temporal understanding of tumor-immune coevolution is required. We addressed this by projecting multi-site HGSOC samples into a pseudotime latent space that recapitulates clinical observations of metastatic dissemination. Within this novel framework, we identified mechanisms of immune escape from a localized to metastatic phenotype.

Tregs are known to foster an immune privilege that promotes tumor progression^41–44^. However, their specific role in the pathogenesis and metastatic progression of HGSOC remains understudied due to limited multi-site datasets. We demonstrated that immune cell infiltration increases with metastatic progression, accompanied by the recruitment of Tregs to counterbalance the abundance of γδ T cells. As γδ T cells display a cytotoxic phenotype without expressing immune checkpoints such as PD1 and CTLA4^45^, metastatic tumors may rely on Tregs to suppress their cytotoxic activity.

Tregs accumulate in ovarian cancer tumors either by recognizing neoantigens and undergoing clonal expansion^46^, or through chemokine-mediated recruitment^44^. Our results demonstrate that early-stage tumors rely more on antigen presentation to recruit Tregs, whereas later-stage metastases increasingly depend on chemokine signaling and vasculature development. We posit that the frequent loss of HLA expression observed in metastasizing ovarian cancer cells drives a shift away from antigen-driven Treg recruitment^17^.

Genomic instability promotes metastasis by inducing the release of DNA into the cytoplasm, thus activating the STING pathway and downstream noncanonical NF-κB signaling^20^. In HGSOC, genomic instability caused by BRCA1 mutations promotes STING activation, resulting in T cell infiltration and immune suppression via VEGF^47^. We demonstrate that during metastatic progression, HGSOC tumors leverage SNX8, a critical component of the STING pathway^19^, to increase the abundance of Tregs.

Previous studies in HGSOC have demonstrated that T cells infiltrating the tumor epithelium primarily consist of exhausted CD8 + T cells^23,24^. We demonstrated that the infiltration of CD8+ TILs into the tumor epithelium correlates with metastatic progression, and coincides with high levels of exhaustion. These findings suggest that during metastasis, the infiltration pattern of CD8⁺ TILs is closely linked to their intrinsic phenotype and exhaustion state. In contrast, the exclusion CD4+ TILs from the epithelium correlated with metastatic progression. However, the specific CD4+ phenotype underlying this exclusion remains unknown and warrants further investigation.

As tumors progress, they establish a hostile microenvironment-characterized by nutrient scarcity, waste accumulation, acidity, and hypoxia-that restricts T cell activity^48^. Our data reveal that metabolic activity, particularly bile acid metabolism and glycolysis, modulate the ITH of both CD4⁺ and CD8⁺ TILs in primary ovarian and metastatic omental tumors. In particular, CD8⁺ TILs tend to exhibit a dominant phenotype that varies significantly across tumors, whereas CD4⁺ TILs show a more uniform phenotype across patients, perhaps reflecting distinct responses to the metabolic selection pressures within the tumor milieu. Given that ascites serves as a dynamic reservoir of metabolites, including glucose and cholesterol, and modulates T cell function^49^, future studies are needed to elucidate its impact on TIL ITH.

scRNA-seq of the fallopian epithelium identified a cell phenotype characterized by keratin expression and upregulation of MHC class II genes^50^. In a mouse model, keratin expressing stromal cells present antigens via MHC class II, maintaining an active population of Tregs that constrains antigen-specific immunity^33^. We demonstrated that ovarian cancer cells in early-stage disease likely retain this mechanism from their epithelial origin. This results in an inflammatory phenotype characterized by limited IFNγ production, restricted B and T cell clonal expansion, and ultimately worse patient prognosis.

Overall, this study identifies mechanisms of immune evasion that permit localized disease and drive metastatic progression in HGSOC. The aberrant role of Tregs at each stage of disease underscores the importance of directly targeting this cell type to enhance immunotherapy outcomes for HGSOC patients.

## Methods

### Immunohistochemistry

The tissue was obtained during laparoscopic biopsy or primary cytoreductive surgery, classified by the tumor’s anatomical location (Supplementary Fig. 4A), and subsequently formalin-fixed and paraffin-embedded. Sections were stained with hematoxylin and eosin, visualized with diaminobenzidine, and counterstained with hematoxylin per the manufacturer’s instructions. The slides were dehydrated, cleared, and permanently mounted. This involved 4 μm thick tissue sections mounted onto a “charged “glass slide and baked at 60 degrees for 1 h. The slides were then labeled and loaded on the Leica Bond III immunostainer. The sections were stained with either CD4 (Leica Ready to Use antibody CD4 PA0427 Clone 4B12) or CD8 (Leica Ready to Use antibody CD8 PA0183 Clone 4B11) on an IHC autostainer. The slides were dewaxed, antigens were retrieved, and then the slides were dehydrated, cleared, and coverslipped. The stained slides scanned using an Aperio AT2 digital slide scanner (Leica Biosystems Ltd, Germany). The scanned images were annotated with a consultant pathologist to assess intra-epithelial and stromal CD4 and CD8 expression using ImageScope (Leica Biosystems Ltd), and analyzed using the Aperio Nuclear Algorithm v9 (Leica Biosystems Ltd). Ovarian tumor epithelial cells were distinguished from stromal cells by the shape and size of their nuclei. The TIL density was calculated as the number of CD4 and CD8 positive cells per mm2.

### Global proteomics analyses

High resolution liquid chromatography-tandem mass spectrometry (LC-MS/MS) analysis with Tandem Mass Tag (TMT) labeling was used, as described previously with modifications^51^. FFPE tissues were deparaffinized and proteins were extracted and digested with trypsin as described^52^. Two micrograms of protein from each sample were combined to produce a common control reference sample and 20 μg of protein from each sample and a 20 μg aliquot of the common control were TMT labeled with ThermoFisher Scientific 18-plex reagent kits according to the manufacturer’s instructions. Samples were combined in equal amounts and separated into 8 fractions with high pH reverse phase fractionation spin columns. LC-MS/MS analyses of each fraction were performed with an Orbitrap Fusion Lumos mass spectrometer coupled to an Ultimate 3000 nano-LC. Peptides were separated over a 3 h gradient from 4 to 25% acetonitrile in 0.1% formic acid at a flow rate of 300 nL/min on a Thermo Scientific EasySpray 50 cm × 75 µm, 2 µm particle size RP C18 column. Full scan MS spectra were acquired in the Orbitrap at a resolution of 120,000. The most intense MS1 ions were selected fragmentation in the ion trap at 35% collision energy and an isolation width of 1.2 Da. Six fragment ions were co-isolated using synchronous precursor selection with an isolation width of 1.3 Da and fragmented by HCD with a normalized collision energy of 65%. The product ions were analyzed using the Orbitrap at a resolution of 50,000. Peptide and protein identifications and relative quantification were done with Proteome Discoverer® v. 2.5 software.

The relative abundances were log2 transformed and zero-centered for each protein to obtain final, relative abundance values. Proteins absent in 50% or more of samples in any tumor site were filtered out, followed by imputation across tumor site using the “scImpute” function and tail-based imputation for each sample using the “tImpute” function from PhosR.

### Pseudotime analysis

Pseudotime analysis was performed on global proteomic data from 23 patients (*n* = 80 samples) encompassing both primary and metastatic tumors, using the PhenoPath R package^6^. To account for multiple samples per patient, patient identity was included as a covariate in the model. Pathway associations were assessed by calculating Spearman’s rho between pseudotime and hallmark pathway activity scores (inferred with single-sample GSEA). Benjamini-Hochberg correction was applied to adjust for multiple testing.

### TME deconvolution

TME cell composition was estimated using the ConsensusTME method with ovarian cancer-specific gene sets^16^. The coefficient of variation for each cell type was calculated. To assess the significance of cell type variation differences, a null distribution was generated through a permutation test (100,000 replicates) without replacement.

### Validation analysis in TCGA

RNA-seq reads were mapped to the human transcriptome (NCBI Build 38, GRCh38) using Kallisto^53^. Gene expression levels, measured in transcripts per million (TPM) for each sample, were obtained through the Sleuth workflow. To avoid duplicate entries, Ensembl gene IDs corresponding to multiple external gene name identifiers were aggregated. TPM values were then log₂-transformed for downstream analyses. Patients were categorized into high or low SNX8 expression groups using a 75th percentile cutoff.). Treg abundance was inferred from the RNA-seq data using ConsensusTME and compared between groups^16^.

### The polar coordinate system

The density of CD4+ and CD8+ TILs in the epithelium and stroma were converted into polar coordinates, defined by the following equations: (1) T cell quantity (R) = square root [(TILs tumor)2 + (TILs stroma)2]^2^; (2) T cell spatial distribution (θ) = [atan (TILs stroma/TILs tumor)]. Multiple linear regression was used to determine the coefficients between the polar coordinates, pseudotime and T cell phenotypes, which were then compared using a T-test. The signatures for CD8 + T cell phenotypes were accessed from the supplemental data of Sade Feldman et al.^25^, and were scored in each sample using single sample GSEA.

### Shannon diversity index analysis

The number of desert, excluded, and infiltrated regions for CD4+ and CD8+ TILs were quantified in each sample. These counts served as input for the entropy.empirical function in the ‘entropy‘ R package, employing the maximum likelihood estimation method with default parameters. Pathways from the Hallmarks gene set, available through MSigDB, were scored in each sample, and their relationship with the SDI values were modeled using lasso regression via the glmnet function in R. The lambda that minimized the cross-validated error was selected with an alpha value of 1. Only non-zero coefficients were retained for further analysis. To assess the stability of the lasso model, we implemented a bootstrap procedure with 10,000 replicates. For each coefficient, we computed the bootstrap mean, as well as both percentile and bias-corrected accelerated (BCa) 95% confidence intervals, and recorded the proportion of replicates in which the coefficient was non-zero.

### Single cell RNA-seq integration and cell-cell communication analysis

Two independent scRNA-seq datasets were accessed from the TISCH database under accession numbers GSE147082 and GSE118828. The datasets were processed using Seurat and integrated with Harmony. A keratin signature score (KRT1, KRT2, KRT9, KRT10) and an MCH class II signature score (from the Reactome database), were computed using Seurat’s AddModuleScore function. The correlation between MHC class II activity and the keratin score was calculated in cancer cells only.

### Differential expression and gene set enrichment analysis

Differentially expressed proteins were identified using the limma R package, with patient identity and tumor site included as a covariate. GSEA was conducted using the fgsea package in R. The Gene Ontology gene sets from MSigDB were used. Pre-ranked gene lists, generated based on t-statistics (for infiltrated vs. non-infiltrated tumors) or Pearson correlation coefficients (for co-expressed genes), served as input. An FDR threshold of 0.05 was applied.

### The external transcriptomic and multicolor immunofluorescence dataset

The processed transcriptomics and immunofluorescence data was accessed at https://github.com/cansysbio/HGSOC_TME_Heterogeneity. The number of CD4 + , CD8+ and Treg counts were averaged across each region in a sample and converted to a fraction of total counts for downstream analyses. The relationship between IFI44L expression and the mean expression of KRT1, KRT2, KRT9, and KRT10 with T cell infiltration was determined using independent linear regression models. The resulting coefficients were compared using a T-test.

### The external proteogenomic dataset

The processed proteomics data was accessed at https://pdc.cancer.gov/pdc/cptac-pancancer, and the TCGA molecular subtype annotations were available from the supplemental information of the paper. The mean protein expression of KRT1, KRT2, KRT9, and KRT10 was calculated in each sample, and compared between the TCGA molecular subtypes using an Anova test.

### T and B cell clonal composition analysis

The raw Nanostring counts data were accessed under accession number EGAS00001002839 and normalized using DESeq2. The genes identified as upregulated in infiltrated samples (logFC > 0; *p* < 0.05) were scored using the GSVA package and compared between the immune phenotypes characterized by Zhang et al. The metrics defining T and B cell clonal composition data were accessed from the supplementary data^1^. The partial Spearman correlation was calculated between the infiltrated signature, and metrics quantifying T and B cell clonal composition, controlling for the patient of origin.

## Supplementary information

Supplementary Information

## Data Availability

The data that support the findings of this study have been deposited on MassIVE under ID MSV000094907 and are publicly available as of the date of publication. The principal analysis code used to analyze data and generate the results presented here are available at https://github.com/DonaghEgan/Temporal-Model-HGSOC. Any additional information required to reanalyze the data reported in this paper is available from the lead contact upon request.
