# Supplementary material for: A temporal model of tumor-immune dynamics during the metastatic progression of high-grade serous ovarian cancer
Source: NPJ Precis Oncol. 2025 Jun 16;9:188. doi: 10.1038/s41698-025-00973-y (PMC12170838; doi:10.1038/s41698-025-00973-y)
Supplement: Supplementary file 1 — Supplementary Information [file 41698_2025_973_MOESM1_ESM.pdf]

Supplementary Figures and Tables

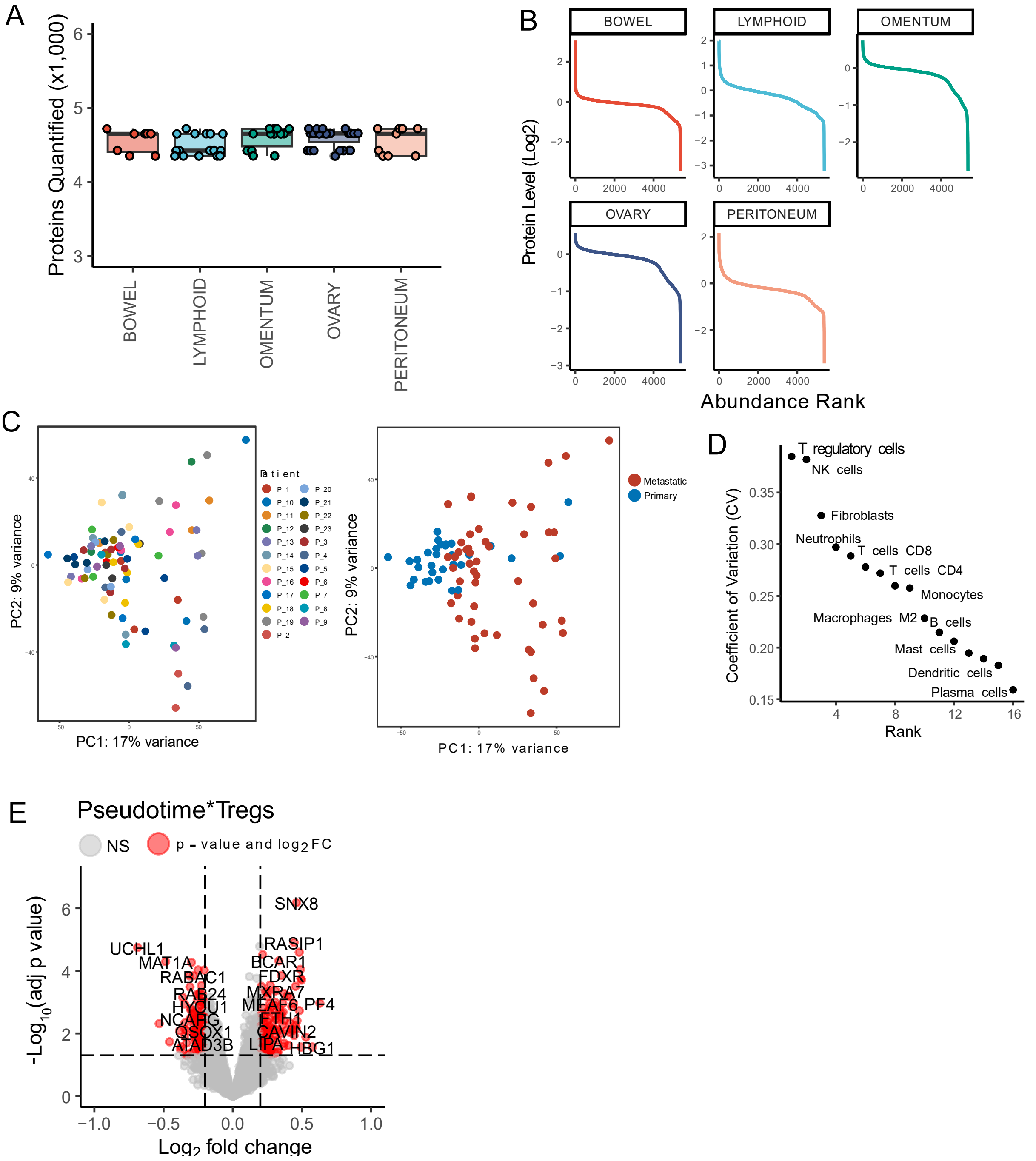

Supplementary Figure 1. A) The number of proteins identified in each tumor site. **B)** The protein expression level and corresponding rank for each tumor site (dynamic range). **C)** Principal component analysis on the proteomic profiles of HGSOC tumor samples, annotated by patient identity and tumor site, respectively. **D)** The coefficient of variation (CV) for each cell type across the tumor samples. **E)** Proteins with

increasing (logFC > 0) or decreasing (logFC < 0) correlations with Tregs as a function of pseudotime.

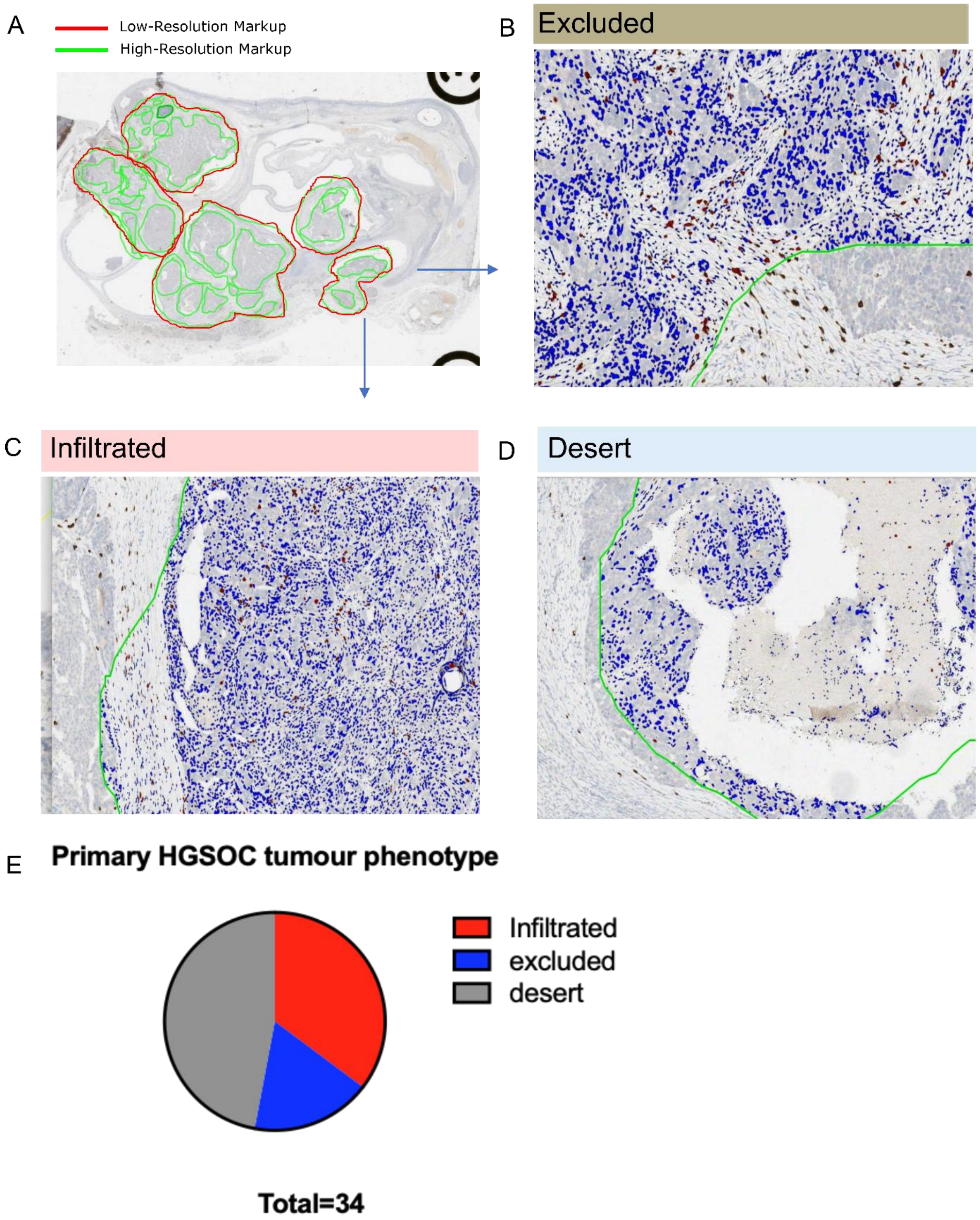

Supplementary Figure 2. A) A primary ovarian sample stained for CD8+ T cells. The red lines denote the approach with fewer analyzed regions, while the green lines indicate the increased number of regions analyzed. **B-D)** x20 magnifications of the same sample demonstrating regions with an excluded, infiltrated, and desert immune phenotype, respectively. **E)** The proportion of each immune phenotype in the primary ovarian sample. 34 regions were examined at x20 magnification.

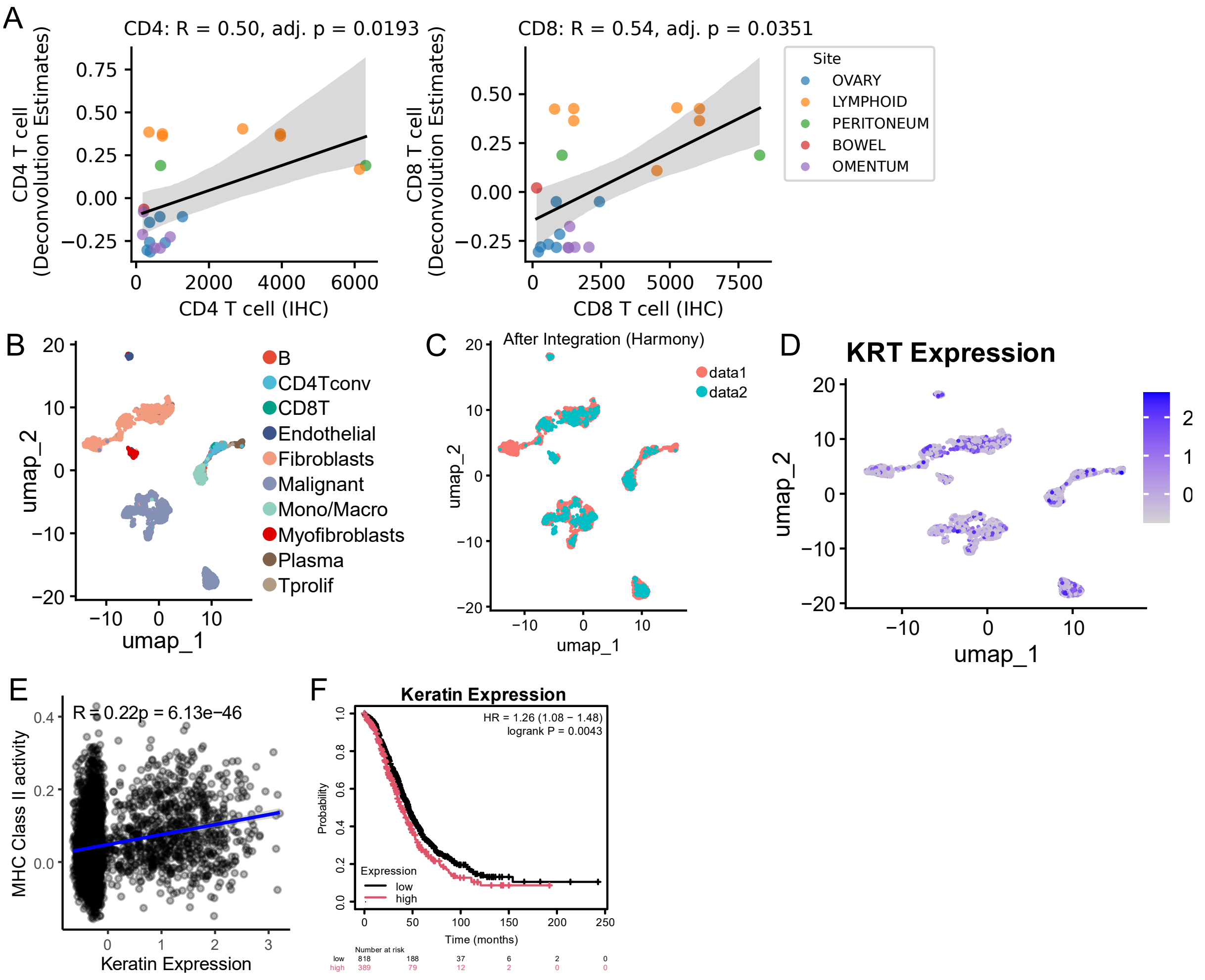

Supplementary Figure 3. A) The correlation between TIL densities from IHC (sum of iTILs and sTILs) and deconvolution-derived estimates of T cell abundances from global proteomics, calculated separately for CD8+ T cells (left panel) and CD4+ T cells (right panel). **B-D)** UMAP of cells from two integrated single cell RNA-seq datasets for ovarian cancer, annotated according to cell type, dataset of origin, and keratin

signature score, respectively. The keratin signature contains KRT1, 2, 9 and 10. **E)** The correlation between keratin expression (KRT1, 2, 9 and 10) and MHC class II activity (from reactome database) in the integrated single cell RNA-seq dataset. **F)** Kaplan Meier overall survival curve for high and low expression of keratin (KRT1, 2, 9 and 10). Patients were dichotomized according to the 75th percentile.

A

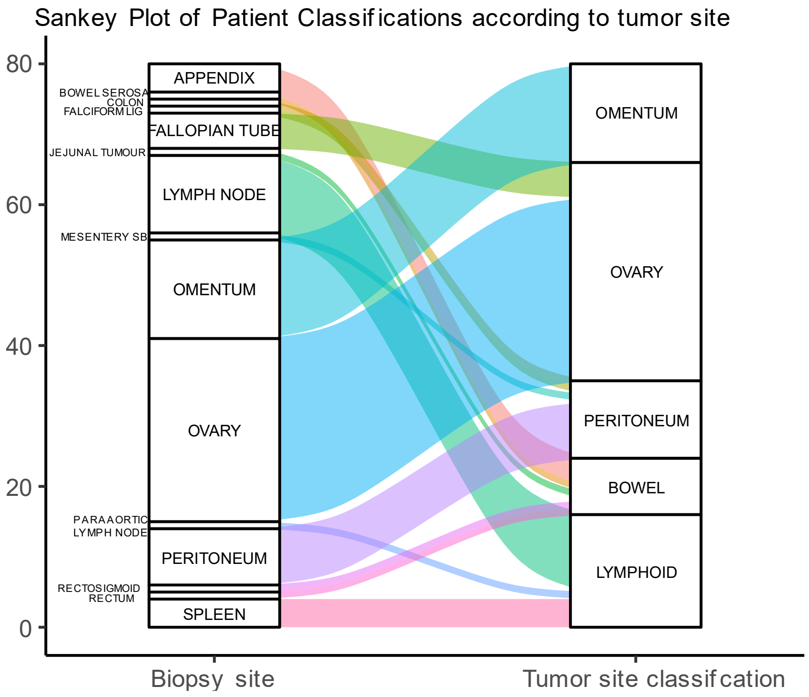

Supplementary Figure 4. A) Sankey diagram illustrating the classification of patient tumor sites based on the anatomical location of the biopsy (n = 80).

Supplementary Table 1. Clinical Data and identifiers for the global proteomic sample.

Protypia ID	MRN	metastatic	patient_no	site_grouped	
28	1241653	Metastatic	P_5	BOWEL	
35	1117620	Metastatic	P_12	BOWEL	
38	753315	Metastatic	P_15	BOWEL	
46	1163757	Metastatic	P_23	BOWEL	
48	1263315	Metastatic	P_3	BOWEL	
68	1274742	Metastatic	P_1	BOWEL	
78	1193857	Metastatic	P_17	BOWEL	
82	886622	Metastatic	P_22	BOWEL	
23	1272126	Metastatic	P_2	LYMPHOID	PARA-AORTIC
25	1272126	Metastatic	P_2	LYMPHOID	PARA-AORTIC
27	1254857	Metastatic	P_4	LYMPHOID	SPLEEN
31	1240597	Metastatic	P_8	LYMPHOID	SPLEEN
32	1241142	Metastatic	P_9	LYMPHOID	SPLEEN
40	1193857	Metastatic	P_17	LYMPHOID	PARA-AORTIC
41	948968	Metastatic	P_18	LYMPHOID	PARA-AORTIC
42	1191805	Metastatic	P_19	LYMPHOID	SPLEEN
47	1274742	Metastatic	P_1	LYMPHOID	PARA-AORTIC
51	843767	Metastatic	P_6	LYMPHOID	PARA-AORTIC
56	715115	Metastatic	P_11	LYMPHOID	PARA-AORTIC
70	1254857	Metastatic	P_4	LYMPHOID	PARA-AORTIC
71	1241653	Metastatic	P_5	LYMPHOID	PARA-AORTIC
72	1240597	Metastatic	P_8	LYMPHOID	PARA-AORTIC
73	1232460	Metastatic	P_10	LYMPHOID	PARA-AORTIC
75	1207105	Metastatic	P_14	LYMPHOID	PARA-AORTIC
24	1274742	Metastatic	P_1	OMENTUM	
26	1263315	Metastatic	P_3	OMENTUM	
36	1221741	Metastatic	P_13	OMENTUM	
37	1207105	Metastatic	P_14	OMENTUM	
39	1197606	Metastatic	P_16	OMENTUM	
49	1254857	Metastatic	P_4	OMENTUM	

50	1241653	Metastatic	P_5	OMENTUM	
53	1240597	Metastatic	P_8	OMENTUM	
54	1241142	Metastatic	P_9	OMENTUM	
55	1232460	Metastatic	P_10	OMENTUM	
66	886622	Metastatic	P_22	OMENTUM	
67	1163757	Metastatic	P_23	OMENTUM	
76	753315	Metastatic	P_15	OMENTUM	
79	948968	Metastatic	P_18	OMENTUM	
1	1274742	Primary	P_1	OVARY	
2	1263315	Primary	P_3	OVARY	
3	1254857	Primary	P_4	OVARY	
4	1241653	Primary	P_5	OVARY	
5	843767	Primary	P_6	OVARY	
6	1240869	Primary	P_7	OVARY	
7	1240597	Primary	P_8	OVARY	
8	1241142	Primary	P_9	OVARY	
9	1232460	Primary	P_10	OVARY	
10	715115	Primary	P_11	OVARY	
11	1117620	Primary	P_12	OVARY	
12	1221741	Primary	P_13	OVARY	
13	1207105	Primary	P_14	OVARY	
14	753315	Primary	P_15	OVARY	
15	1197606	Primary	P_16	OVARY	
16	1193857	Primary	P_17	OVARY	
17	948968	Primary	P_18	OVARY	
18	1191805	Primary	P_19	OVARY	
19	1187532	Primary	P_20	OVARY	
20	1026036	Primary	P_21	OVARY	
21	886622	Primary	P_22	OVARY	
22	1163757	Primary	P_23	OVARY	
30	1240869	Primary	P_7	OVARY	
34	715115	Primary	P_11	OVARY	
43	1187532	Primary	P_20	OVARY	
44	1026036	Primary	P_21	OVARY	
52	1240869	Primary	P_7	OVARY	
57	1221741	Primary	P_13	OVARY	
64	1187532	Primary	P_20	OVARY	
65	1026036	Primary	P_21	OVARY	
81	1026036	Primary	P_21	OVARY	
33	1232460	Metastatic	P_10	PERITONEUM	
45	886622	Metastatic	P_22	PERITONEUM	
58	1207105	Metastatic	P_14	PERITONEUM	
59	753315	Metastatic	P_15	PERITONEUM	
60	1197606	Metastatic	P_16	PERITONEUM	

62	948968	Metastatic	P_18	PERITONEUM	
63	1191805	Metastatic	P_19	PERITONEUM	
69	1263315	Metastatic	P_3	PERITONEUM	
74	1221741	Metastatic	P_13	PERITONEUM	
77	1197606	Metastatic	P_16	PERITONEUM	
80	1191805	Metastatic	P_19	PERITONEUM	

Supplementary Table 2. The genes significantly correlated with pseudotime (FDR < 0.05). The LogFC reflects the coefficient estimate as Limma was used with pseudotime as the response variable.

logFC	AveExpr	t	P.Value	adj.P.Val	B	Gene	pathway
3.091509	-0.14395	9.482636	7.47E-15	3.99E-11	23.27918	MPP1	HEME_METABOLISM
-1.38425	-0.04625	-7.72286	2.39E-11	2.28E-08	15.54998	SNRPB	E2F_TARGETS
3.337816	-0.37213	7.707998	2.55E-11	2.28E-08	15.48508	RAC2	APICAL_JUNCTION
3.138654	-0.24363	7.706915	2.57E-11	2.28E-08	15.48036	STOM	c("ADIPOGENESIS", "P53_PATHWAY")
2.617938	-0.16025	7.660446	3.17E-11	2.42E-08	15.2776	PECAM1	c("APICAL_JUNCTION", "COAGULATION", "KRAS_SIGNALING_UP")
3.22554	-0.42765	7.251623	2.02E-10	8.99E-08	13.50209	PLEK	c("COAGULATION", "COMPLEMENT", "TNFA_SIGNALING_VIA_NFKB")
-1.91987	-0.15675	-7.16742	2.95E-10	1.10E-07	13.13859	CTNND1	APICAL_JUNCTION
-1.83233	-0.1595	-7.09459	4.10E-10	1.29E-07	12.82486	CTNNA1	APICAL_JUNCTION
3.482741	-0.45563	7.067971	4.62E-10	1.37E-07	12.71039	ITGAL	ALLOGRAFT_REJECTION
2.228268	-0.08321	7.031398	5.44E-10	1.49E-07	12.55325	ARRB1	NOTCH_SIGNALING

3.360399	-0.4155	7.00429	6.14E-10	1.49E-07	12.43689	DOCK2	c("KRAS_SIGNALING_UP", "MITOTIC_SPINDLE")
2.958669	-0.286	6.988699	6.58E-10	1.53E-07	12.37002	WAS	c("ALLOGRAFT_REJECTION", "COMPLEMENT")
2.097158	-0.09814	6.917579	9.05E-10	1.80E-07	12.06539	CPQ	c("COAGULATION", "COMPLEMENT")
-1.44076	-0.06625	-6.78381	1.64E-09	2.74E-07	11.49451	NUDT21	c("DNA_REPAIR", "E2F_TARGETS")
2.692494	-0.15663	6.724272	2.14E-09	3.33E-07	11.24134	ME1	c("ADIPOGENESIS", "COMPLEMENT", "FATTY_ACID_METABOLISM", "GLYCOLYSIS", "MTORC1_SIGNALING")
3.662359	-0.60475	6.719619	2.18E-09	3.33E-07	11.22158	CORO1A	MTORC1_SIGNALING
2.579698	-0.27463	6.619497	3.40E-09	4.32E-07	10.79728	CTSS	c("ALLOGRAFT_REJECTION", "COMPLEMENT", "KRAS_SIGNALING_UP")
2.504635	-0.18796	6.514831	5.39E-09	5.87E-07	10.35567	SNCA	HEME_METABOLISM
2.20013	-0.17295	6.507054	5.58E-09	5.96E-07	10.32294	TNFAIP8	c("ANDROGEN_RESPONSE", "TNFA_SIGNALING_VIA_NFKB")
1.606159	-0.15807	6.502625	5.69E-09	5.96E-07	10.3043	RABEP1	ESTROGEN_RESPONSE_LATE
2.333052	-0.2195	6.398553	8.98E-09	8.56E-07	9.867539	GLRX	c("GLYCOLYSIS", "HYPOXIA", "KRAS_SIGNALING_UP", "MTORC1_SIGNALING", "REACTIVE_OXYGEN_SPECIES_PATHWAY")
-2.18816	-0.25538	-6.38617	9.48E-09	8.88E-07	9.815699	KRT8	c("ANDROGEN_RESPONSE", "ESTROGEN_RESPONSE_EARLY")
-1.53241	-0.1785	-6.37742	9.85E-09	8.91E-07	9.779131	BCL2L1	c("APOPTOSIS", "IL2_STAT5_SIGNALING")
-2.01294	-0.20188	-6.35716	1.08E-08	9.33E-07	9.694432	CTNNB1	c("APOPTOSIS", "CHOLESTEROL_HOMEOSTASIS", "TGF_BETA_SIGNALING",

							"WNT_BETA_CATENIN_SIGNALING")
2.26052	-0.07913	6.350886	1.11E-08	9.33E-07	9.668235	EPB41	c("HEME_METABOLISM", "MITOTIC_SPINDLE")
-1.6527	-0.09788	-6.34713	1.12E-08	9.33E-07	9.65255	MRPL15	c("ADIPOGENESIS", "OXIDATIVE_PHOSPHORYLATION")
-1.45547	-0.07475	-6.33225	1.20E-08	9.42E-07	9.590437	TRA2B	c("E2F_TARGETS", "G2M_CHECKPOINT", "MYC_TARGETS_V1")
2.594376	-0.24725	6.315544	1.29E-08	9.82E-07	9.520769	CYBB	INFLAMMATORY_RESPONSE
2.052137	-0.30994	6.312605	1.31E-08	9.82E-07	9.508519	LITAF	TNFA_SIGNALING_VIA_NFKB
-1.44334	-0.09125	-6.30224	1.37E-08	1.00E-06	9.465331	MRPL11	OXIDATIVE_PHOSPHORYLATION
-2.2293	-0.24625	-6.27562	1.53E-08	1.07E-06	9.354512	KRT19	c("ANDROGEN_RESPONSE", "ESTROGEN_RESPONSE_EARLY", "ESTROGEN_RESPONSE_LATE")
-1.27662	-0.01675	-6.26302	1.62E-08	1.11E-06	9.302108	AAAS	DNA_REPAIR
-1.29156	-0.026	-6.19979	2.13E-08	1.36E-06	9.039721	NUP205	c("E2F_TARGETS", "MTORC1_SIGNALING")
1.363251	-0.07025	6.175848	2.37E-08	1.41E-06	8.940577	GNAI2	c("APICAL_JUNCTION", "COMPLEMENT")
-1.08702	-0.071	-6.1586	2.55E-08	1.46E-06	8.86924	SF3B3	MYC_TARGETS_V1
-2.33075	-0.30963	-6.10673	3.19E-08	1.67E-06	8.655187	KRT18	c("APOPTOSIS", "ESTROGEN_RESPONSE_EARLY", "PROTEIN_SECRETION")
3.090067	-0.62138	6.074852	3.66E-08	1.88E-06	8.523927	ITGB3	c("COAGULATION", "EPITHELIAL_MESENCHYMAL_TRANSITION", "IL6_JAK_STAT3_SIGNALING", "INFLAMMATORY_RESPONSE", "UV_RESPONSE_DN")

2.381355	-0.24363	6.072745	3.69E-08	1.88E-06	8.515259	AMPD3	HYPOXIA
-2.27933	-0.22275	-6.06753	3.78E-08	1.90E-06	8.493816	JUP	c("APICAL_JUNCTION", "KRAS_SIGNALING_UP", "XENOBIOTIC_METABOLISM")
-2.03878	-0.40911	-6.05133	4.05E-08	1.98E-06	8.427229	HDAC3	P53_PATHWAY
-1.56851	-0.12272	-6.01738	4.68E-08	2.23E-06	8.287956	POLR1C	DNA_REPAIR
-0.99495	-0.01738	-6.01057	4.82E-08	2.28E-06	8.260038	NXF1	UV_RESPONSE_UP
2.407819	-0.26663	5.989912	5.27E-08	2.40E-06	8.175444	WIPF1	EPITHELIAL_MESENCHYMAL_TRANSITION
2.398493	-0.43318	5.982959	5.43E-08	2.45E-06	8.146999	LCK	c("ALLOGRAFT_REJECTION", "BILE_ACID_METABOLISM", "COMPLEMENT", "INFLAMMATORY_RESPONSE", "PI3K_AKT_MTOR_SIGNALING")
3.763546	-0.79713	5.981881	5.45E-08	2.45E-06	8.142591	CAVIN2	ADIPOGENESIS
-1.26991	-0.01725	-5.96922	5.76E-08	2.52E-06	8.090838	NUP58	UV_RESPONSE_UP
3.856555	-0.40963	5.966044	5.83E-08	2.53E-06	8.077852	ANK1	HEME_METABOLISM
2.483454	-0.25833	5.90858	7.46E-08	3.10E-06	7.843494	LY75	ALLOGRAFT_REJECTION
3.295358	-0.57925	5.906796	7.51E-08	3.10E-06	7.836236	EVL	APICAL_JUNCTION
3.128056	-0.557	5.904684	7.58E-08	3.10E-06	7.82764	LCP1	KRAS_SIGNALING_UP
2.926919	-0.75725	5.862429	9.07E-08	3.56E-06	7.655916	HMOX1	c("APOPTOSIS", "HYPOXIA", "IL6_JAK_STAT3_SIGNALING", "P53_PATHWAY", "UV_RESPONSE_UP", "XENOBIOTIC_METABOLISM")

-1.66468	-0.22213	-5.85863	9.22E-08	3.59E-06	7.640516	SRSF6	DNA_REPAIR
-1.19739	-0.07225	-5.85705	9.28E-08	3.59E-06	7.634085	SRSF1	c("E2F_TARGETS", "G2M_CHECKPOINT", "MYC_TARGETS_V1")
2.290058	-0.1255	5.842655	9.87E-08	3.74E-06	7.575719	ITGB2	c("ALLOGRAFT_REJECTION", "KRAS_SIGNALING_UP", "MTORC1_SIGNALING")
-1.30644	-0.01188	-5.83715	1.01E-07	3.77E-06	7.55341	PRPF4B	G2M_CHECKPOINT
2.017632	-0.20096	5.832357	1.03E-07	3.82E-06	7.533997	GNB4	COMPLEMENT
-1.6135	-0.1635	-5.82542	1.06E-07	3.91E-06	7.505913	HDGF	c("HEME_METABOLISM", "MYC_TARGETS_V1")
1.645944	-0.06525	5.813161	1.12E-07	4.05E-06	7.456303	RHOG	c("COMPLEMENT", "INFLAMMATORY_RESPONSE")
3.742829	-0.42313	5.807981	1.14E-07	4.05E-06	7.435355	SPTB	HEME_METABOLISM
1.13735	-0.02138	5.805293	1.16E-07	4.05E-06	7.424487	STX12	PROTEIN_SECRETION
-1.28157	-0.00413	-5.79768	1.19E-07	4.11E-06	7.393718	FARP1	c("ESTROGEN_RESPONSE_EARLY", "ESTROGEN_RESPONSE_LATE", "MITOTIC_SPINDLE")
-1.31477	-0.16725	-5.79223	1.22E-07	4.16E-06	7.371707	TIMM10	OXIDATIVE_PHOSPHORYLATION
3.737836	-0.18228	5.785917	1.26E-07	4.24E-06	7.346215	EPB42	HEME_METABOLISM
-1.15007	-0.1125	-5.78165	1.28E-07	4.29E-06	7.329005	RBM4	KRAS_SIGNALING_UP
2.123398	-0.18375	5.760414	1.40E-07	4.63E-06	7.243352	STAB1	c("ALLOGRAFT_REJECTION", "INFLAMMATORY_RESPONSE")
3.64922	-0.36938	5.757546	1.42E-07	4.64E-06	7.231792	SPTA1	HEME_METABOLISM

1.854191	-0.05388	5.739491	1.53E-07	4.87E-06	7.159098	LGMN	c("CHOLESTEROL_HOMEOSTASIS", "COAGULATION", "COMPLEMENT", "MTORC1_SIGNALING")
2.190637	-0.136	5.738722	1.53E-07	4.87E-06	7.156002	RAB27A	UV_RESPONSE_UP
-1.248	-0.02063	-5.73674	1.55E-07	4.88E-06	7.148022	PUS1	c("IL2_STAT5_SIGNALING", "MYC_TARGETS_V2")
2.966398	-0.46	5.723385	1.63E-07	5.13E-06	7.094326	HCLS1	c("ALLOGRAFT_REJECTION", "DNA_REPAIR")
2.527434	-0.33863	5.6919	1.87E-07	5.70E-06	6.96793	FCN1	COMPLEMENT
-1.67324	-0.1025	-5.69181	1.87E-07	5.70E-06	6.967551	ELF3	c("ESTROGEN_RESPONSE_EARLY", "GLYCOLYSIS")
-1.59372	-0.164	-5.65685	2.16E-07	6.41E-06	6.82755	MDH2	c("ADIPOGENESIS", "FATTY_ACID_METABOLISM", "GLYCOLYSIS", "OXIDATIVE_PHOSPHORYLATION")
-1.3329	-0.12088	-5.65475	2.18E-07	6.43E-06	6.819156	VDAC1	c("MYC_TARGETS_V1", "OXIDATIVE_PHOSPHORYLATION")
-1.26383	-0.07463	-5.64164	2.30E-07	6.66E-06	6.766787	DAP3	APOPTOSIS
-1.85893	-0.199	-5.62419	2.48E-07	6.89E-06	6.697095	BAIAP2	c("APICAL_JUNCTION", "P53_PATHWAY")
2.5042	-0.33252	5.600739	2.73E-07	7.45E-06	6.603626	PRKCB	c("ALLOGRAFT_REJECTION", "PI3K_AKT_MTOR_SIGNALING")
-1.05546	-0.06938	-5.59258	2.83E-07	7.59E-06	6.571161	SNRPA	MYC_TARGETS_V1
-1.05434	-0.05625	-5.5762	3.03E-07	7.85E-06	6.505997	KHSRP	UNFOLDED_PROTEIN_RESPONSE
-1.05399	-0.04925	-5.56548	3.17E-07	8.13E-06	6.463404	DLG1	c("APICAL_JUNCTION", "MITOTIC_SPINDLE", "UV_RESPONSE_DN")
2.542991	-0.07641	5.550885	3.37E-07	8.45E-06	6.405472	PTK2B	ANDROGEN_RESPONSE

-1.51813	-0.06725	-5.53581	3.58E-07	8.94E-06	6.345722	MIX23	FATTY_ACID_METABOLISM
1.265961	-0.26701	5.529441	3.68E-07	9.14E-06	6.320477	NPC1	c("BILE_ACID_METABOLISM", "XENOBIOTIC_METABOLISM")
2.564202	-0.30221	5.528297	3.70E-07	9.14E-06	6.315947	CLIC2	HEME_METABOLISM
-1.07902	-0.09588	-5.51449	3.92E-07	9.59E-06	6.261285	SNRPD2	MYC_TARGETS_V1
2.024366	-0.29988	5.50445	4.08E-07	9.86E-06	6.221605	VCAM1	c("APICAL_JUNCTION", "EPITHELIAL_MESENCHYMAL_TRANSITION", "INTERFERON_GAMMA_RESPONSE")
4.053595	-0.58863	5.502159	4.12E-07	9.91E-06	6.212551	CA1	HEME_METABOLISM
1.963182	-0.23189	5.49822	4.19E-07	1.00E-05	6.19699	UBE2J1	ANDROGEN_RESPONSE
-1.45329	-0.12175	-5.49171	4.30E-07	1.03E-05	6.171284	FUS	UNFOLDED_PROTEIN_RESPONSE
3.133767	-0.34039	5.4866	4.41E-07	1.04E-05	6.148742	DMTN	HEME_METABOLISM
1.0057	-0.02238	5.481303	4.49E-07	1.05E-05	6.13021	UGP2	c("GLYCOLYSIS", "HYPOXIA")
-1.23849	-0.099	-5.44399	5.24E-07	1.18E-05	5.983234	ILF3	c("E2F_TARGETS", "G2M_CHECKPOINT")
-2.11165	-0.29238	-5.43674	5.40E-07	1.21E-05	5.95476	CDH1	c("APICAL_JUNCTION", "ESTROGEN_RESPONSE_LATE", "TGF_BETA_SIGNALING")
-1.06038	0.0325	-5.43278	5.49E-07	1.22E-05	5.939197	NUP107	E2F_TARGETS
2.934004	-0.52265	5.421177	5.76E-07	1.27E-05	5.893619	IL16	ALLOGRAFT_REJECTION
3.876698	-0.50325	5.413976	5.93E-07	1.29E-05	5.865366	SLC4A1	HEME_METABOLISM

-1.18886	-0.10325	-5.41294	5.96E-07	1.29E-05	5.861309	LUC7L3	E2F_TARGETS
-1.3101	-0.2465	-5.39434	6.43E-07	1.35E-05	5.788391	NCBP2	c("DNA_REPAIR", "MYC_TARGETS_V1")
2.06145	-0.20185	5.394029	6.44E-07	1.35E-05	5.787185	PLIN2	c("ADIPOGENESIS", "HYPOXIA", "IL2_STAT5_SIGNALING")
2.473869	-0.13671	5.391566	6.51E-07	1.36E-05	5.777542	PDGFRB	c("APOPTOSIS", "EPITHELIAL_MESENCHYMAL_TRANSITION", "UV_RESPONSE_DN")
1.535425	-0.06775	5.390583	6.53E-07	1.36E-05	5.773693	STX7	PROTEIN_SECRETION
-1.10686	-0.0415	-5.37086	7.09E-07	1.44E-05	5.696529	CSTF3	DNA_REPAIR
2.51484	-0.3015	5.366607	7.21E-07	1.45E-05	5.679931	GGA2	MTORC1_SIGNALING
-1.40098	-0.16588	-5.3654	7.25E-07	1.45E-05	5.675216	HSPA9	c("MTORC1_SIGNALING", "OXIDATIVE_PHOSPHORYLATION", "UNFOLDED_PROTEIN_RESPONSE")
2.057082	-0.20588	5.364134	7.28E-07	1.46E-05	5.670271	SLC2A3	c("HYPOXIA", "IL2_STAT5_SIGNALING", "MTORC1_SIGNALING", "TNFA_SIGNALING_VIA_NFKB")
3.578942	-0.77888	5.362428	7.34E-07	1.46E-05	5.663606	S100A9	c("COMPLEMENT", "ESTROGEN_RESPONSE_LATE")
2.220983	-0.117	5.360241	7.40E-07	1.47E-05	5.655068	CAT	c("ADIPOGENESIS", "BILE_ACID_METABOLISM", "HEME_METABOLISM", "PEROXISOME", "REACTIVE_OXYGEN_SPECIES_PATHWAY", "XENOBIOTIC_METABOLISM")
1.741647	-0.199	5.352882	7.63E-07	1.50E-05	5.626345	ADA	c("DNA_REPAIR", "P53_PATHWAY")
-1.08318	-0.094	-5.34606	7.84E-07	1.53E-05	5.599747	HNRNPU	c("G2M_CHECKPOINT", "MYC_TARGETS_V1", "UV_RESPONSE_UP")

-1.27727	-0.09738	-5.32656	8.50E-07	1.64E-05	5.523746	DIABLO	APOPTOSIS
2.554104	-0.15482	5.325564	8.53E-07	1.64E-05	5.519875	CAV2	PROTEIN_SECRETION
-1.5939	-0.202	-5.32311	8.62E-07	1.65E-05	5.510312	HSPE1	c("MTORC1_SIGNALING", "MYC_TARGETS_V1", "MYC_TARGETS_V2")
-1.84561	-0.78118	-5.32289	8.63E-07	1.65E-05	5.509468	ERCC1	c("DNA_REPAIR", "PEROXISOME")
2.579426	-0.28851	5.315543	8.89E-07	1.68E-05	5.480888	FGR	ALLOGRAFT_REJECTION
1.701204	-0.0665	5.306403	9.23E-07	1.73E-05	5.445355	IQGAP2	ANDROGEN_RESPONSE
-1.04496	-0.06338	-5.29822	9.54E-07	1.78E-05	5.413582	SF3A3	DNA_REPAIR
1.972096	-0.28545	5.286662	1.00E-06	1.85E-05	5.368711	MAN1A1	XENOBIOTIC_METABOLISM
-1.82318	-0.22625	-5.27266	1.06E-06	1.93E-05	5.314413	BPHL	c("FATTY_ACID_METABOLISM", "XENOBIOTIC_METABOLISM")
-1.76901	-0.67562	-5.26534	1.09E-06	1.97E-05	5.286074	GPRC5C	KRAS_SIGNALING_DN
-1.00736	-0.0325	-5.25324	1.15E-06	2.06E-05	5.239262	NUP93	INTERFERON_GAMMA_RESPONSE
-2.18432	-0.54059	-5.25043	1.16E-06	2.07E-05	5.2284	HKDC1	KRAS_SIGNALING_UP
-1.15081	-0.09213	-5.21554	1.34E-06	2.31E-05	5.093737	PRKDC	E2F_TARGETS
-1.24072	-0.06888	-5.21497	1.34E-06	2.31E-05	5.091536	PTCD3	ADIPOGENESIS
-1.20194	-0.13564	-5.21139	1.36E-06	2.32E-05	5.077721	SRSF10	G2M_CHECKPOINT

2.005716	-0.268	5.196828	1.44E-06	2.43E-05	5.021677	CASP1	c("APOPTOSIS", "COMPLEMENT", "INTERFERON_ALPHA_RESPONSE", "INTERFERON_GAMMA_RESPONSE", "P53_PATHWAY")
-1.0786	-0.05938	-5.18859	1.49E-06	2.49E-05	4.989985	SRSF2	c("E2F_TARGETS", "G2M_CHECKPOINT", "MYC_TARGETS_V1")
-1.55421	-0.147	-5.17705	1.56E-06	2.57E-05	4.945654	PHB2	c("MYC_TARGETS_V1", "OXIDATIVE_PHOSPHORYLATION")
-1.74279	-0.12038	-5.16827	1.62E-06	2.65E-05	4.911983	GRB7	APICAL_JUNCTION
-1.52992	-0.15288	-5.158	1.69E-06	2.74E-05	4.872574	LRPPRC	c("MITOTIC_SPINDLE", "OXIDATIVE_PHOSPHORYLATION")
4.015741	-0.70513	5.14112	1.80E-06	2.91E-05	4.807945	HBB	HEME_METABOLISM
-2.33373	-0.31688	-5.13416	1.86E-06	2.97E-05	4.781328	PKP3	ESTROGEN_RESPONSE_LATE
1.553173	-0.21075	5.132234	1.87E-06	2.98E-05	4.773958	HTATIP2	HEME_METABOLISM
-1.49787	-0.17688	-5.12299	1.94E-06	3.06E-05	4.738639	ECI1	c("FATTY_ACID_METABOLISM", "OXIDATIVE_PHOSPHORYLATION")
1.638587	-0.11988	5.121377	1.95E-06	3.06E-05	4.732476	DEF6	P53_PATHWAY
-1.45709	-0.10638	-5.11979	1.97E-06	3.07E-05	4.726419	NELFCD	DNA_REPAIR
2.174005	-0.321	5.107356	2.07E-06	3.20E-05	4.678963	PTPN6	c("ALLOGRAFT_REJECTION", "ESTROGEN_RESPONSE_LATE", "INTERFERON_GAMMA_RESPONSE")
-1.60955	-0.5538	-5.08584	2.25E-06	3.43E-05	4.597011	KIF13B	P53_PATHWAY
-0.93843	-0.07013	-5.07274	2.37E-06	3.60E-05	4.547188	SNRPB2	MYC_TARGETS_V1

-1.2949	-0.13575	-5.07191	2.38E-06	3.60E-05	4.544013	ALYREF	DNA_REPAIR
-1.01033	-0.06713	-5.06017	2.50E-06	3.73E-05	4.499419	SF3A1	MYC_TARGETS_V1
-1.30118	-0.04955	-5.04749	2.63E-06	3.90E-05	4.451352	MRPS22	OXIDATIVE_PHOSPHORYLATION
-1.1924	-0.0955	-5.03967	2.71E-06	4.01E-05	4.421687	PNN	E2F_TARGETS
2.556585	-0.62912	5.011776	3.03E-06	4.35E-05	4.316201	LIPA	COMPLEMENT
-1.33026	-0.07225	-5.00799	3.08E-06	4.40E-05	4.301897	TNPO2	G2M_CHECKPOINT
-0.94126	-0.03013	-4.99802	3.20E-06	4.54E-05	4.264298	SNRPA1	MYC_TARGETS_V1
1.658523	-0.10719	4.982271	3.41E-06	4.71E-05	4.204933	DPYD	APOPTOSIS
-0.96991	-0.03525	-4.98048	3.43E-06	4.73E-05	4.198174	BUB3	c("G2M_CHECKPOINT", "MYC_TARGETS_V1")
1.358222	-0.07288	4.979123	3.45E-06	4.75E-05	4.193083	PRPS1	c("E2F_TARGETS", "GLYCOLYSIS")
-1.14181	-0.06352	-4.97769	3.47E-06	4.76E-05	4.18767	MAD2L1	c("E2F_TARGETS", "G2M_CHECKPOINT", "MYC_TARGETS_V1")
-1.26994	-0.11075	-4.97672	3.48E-06	4.77E-05	4.184032	PCM1	MITOTIC_SPINDLE
2.053075	-0.14475	4.962202	3.69E-06	5.00E-05	4.129447	BLVRB	c("ESTROGEN_RESPONSE_EARLY", "ESTROGEN_RESPONSE_LATE", "HEME_METABOLISM", "XENOBIOTIC_METABOLISM")
-1.21	-0.06138	-4.95858	3.74E-06	5.06E-05	4.115843	NUP98	G2M_CHECKPOINT
-1.00106	-0.03588	-4.95569	3.78E-06	5.08E-05	4.10499	SRSF7	MYC_TARGETS_V1

-1.53303	-0.13913	-4.9521	3.84E-06	5.12E-05	4.091528	PCBD1	FATTY_ACID_METABOLISM
-1.15367	-0.11025	-4.95163	3.85E-06	5.12E-05	4.089731	COX5B	OXIDATIVE_PHOSPHORYLATION
-1.55512	-0.193	-4.93759	4.07E-06	5.33E-05	4.037096	ALDH7A1	GLYCOLYSIS
-1.12459	-0.1125	-4.92998	4.19E-06	5.48E-05	4.00859	POLR2H	c("DNA_REPAIR", "UV_RESPONSE_UP")
1.436727	-0.09875	4.927981	4.22E-06	5.51E-05	4.001102	NAGK	HYPOXIA
1.367655	-0.16613	4.926695	4.24E-06	5.51E-05	3.996289	CAP1	APICAL_JUNCTION
-1.34964	-0.07713	-4.92469	4.28E-06	5.54E-05	3.988799	SLC25A12	OXIDATIVE_PHOSPHORYLATION
3.965031	-0.71763	4.921719	4.33E-06	5.60E-05	3.977669	HBD	HEME_METABOLISM
1.703342	-0.06588	4.912924	4.48E-06	5.75E-05	3.944783	ADD2	HEME_METABOLISM
1.596884	-0.03	4.909536	4.54E-06	5.77E-05	3.932123	ALAD	c("FATTY_ACID_METABOLISM", "HEME_METABOLISM")
-1.47365	-0.15438	-4.90124	4.69E-06	5.94E-05	3.901145	TOMM22	OXIDATIVE_PHOSPHORYLATION
1.707925	-0.09725	4.899694	4.72E-06	5.96E-05	3.895369	VIM	EPITHELIAL_MESENCHYMAL_TRANSITION
-1.00324	-0.061	-4.88055	5.09E-06	6.25E-05	3.824011	EIF4A3	UNFOLDED_PROTEIN_RESPONSE
-1.031	0.005375	-4.87951	5.11E-06	6.26E-05	3.820128	WDR33	KRAS_SIGNALING_UP
-1.60551	-0.12063	-4.87319	5.24E-06	6.37E-05	3.796595	ALDH18A1	UNFOLDED_PROTEIN_RESPONSE
-2.24251	-0.55028	-4.87245	5.26E-06	6.38E-05	3.793841	ANXA9	c("ESTROGEN_RESPONSE_EARLY", "ESTROGEN_RESPONSE_LATE")

-1.16975	-0.09175	-4.85884	5.54E-06	6.70E-05	3.743261	NDUFAB1	c("ADIPOGENESIS", "MYC_TARGETS_V1", "OXIDATIVE_PHOSPHORYLATION")
1.325684	-0.078	4.854432	5.64E-06	6.75E-05	3.726871	SNAP23	PROTEIN_SECRETION
-1.48452	-0.19225	-4.85441	5.64E-06	6.75E-05	3.726799	HSPD1	c("MTORC1_SIGNALING", "MYC_TARGETS_V1", "MYC_TARGETS_V2")
1.637787	-0.35471	4.836847	6.04E-06	7.15E-05	3.661633	QSOX1	c("EPITHELIAL_MESENCHYMAL_TRANSITION", "GLYCOLYSIS")
-1.2222	-0.18525	-4.83417	6.11E-06	7.20E-05	3.651703	MOV10	INTERFERON_ALPHA_RESPONSE
2.360234	-0.28925	4.821149	6.43E-06	7.52E-05	3.603508	ITGAM	COMPLEMENT
3.187364	-0.41741	4.803614	6.88E-06	7.92E-05	3.538701	CR1	COMPLEMENT
1.984611	-0.25522	4.799388	7.00E-06	8.02E-05	3.523104	LCP2	c("ALLOGRAFT_REJECTION", "COMPLEMENT", "INFLAMMATORY_RESPONSE", "INTERFERON_GAMMA_RESPONSE")
1.694704	-0.06088	4.788914	7.29E-06	8.26E-05	3.484472	FES	REACTIVE_OXYGEN_SPECIES_PATHWAY
3.431027	-0.80925	4.788601	7.30E-06	8.26E-05	3.483319	S100A12	COMPLEMENT
1.006857	-0.052	4.775807	7.67E-06	8.60E-05	3.436197	RSU1	APICAL_JUNCTION
-1.93834	-0.48565	-4.76283	8.07E-06	8.99E-05	3.388467	LAMC2	c("APICAL_JUNCTION", "EPITHELIAL_MESENCHYMAL_TRANSITION", "ESTROGEN_RESPONSE_LATE")
3.143561	-0.68813	4.745116	8.64E-06	9.51E-05	3.323449	RETN	c("ADIPOGENESIS", "KRAS_SIGNALING_UP")
-1.17716	-0.16688	-4.74433	8.67E-06	9.52E-05	3.320561	ATP5F1E	OXIDATIVE_PHOSPHORYLATION

-1.17575	-0.09025	-4.74339	8.70E-06	9.52E-05	3.317127	MRPS31	UV_RESPONSE_DN
-1.14816	-0.10775	-4.74006	8.81E-06	9.61E-05	3.304903	ILF2	MYC_TARGETS_V1
-1.43613	-0.19575	-4.73509	8.98E-06	9.73E-05	3.286691	SDHC	c("ADIPOGENESIS", "FATTY_ACID_METABOLISM", "GLYCOLYSIS", "OXIDATIVE_PHOSPHORYLATION")
1.130652	-0.01213	4.73412	9.02E-06	9.74E-05	3.28315	STAT5A	c("ADIPOGENESIS", "TNFA_SIGNALING_VIA_NFKB")
-2.05861	-0.29825	-4.73339	9.04E-06	9.75E-05	3.280483	LAD1	ESTROGEN_RESPONSE_EARLY
-0.97979	-0.0965	-4.71785	9.60E-06	1.02E-04	3.22361	HNRNPC	MYC_TARGETS_V1
1.390111	-0.26989	4.706956	1.00E-05	1.05E-04	3.183827	CAMK1D	KRAS_SIGNALING_DN
-1.14757	-0.08563	-4.70531	1.01E-05	1.06E-04	3.177824	ATP5F1C	OXIDATIVE_PHOSPHORYLATION
-1.71841	-0.53433	-4.70497	1.01E-05	1.06E-04	3.176588	CANT1	c("DNA_REPAIR", "ESTROGEN_RESPONSE_EARLY")
-1.20486	-0.09013	-4.70264	1.02E-05	1.06E-04	3.168064	SNRPD3	MYC_TARGETS_V1
2.497828	-0.03101	4.702407	1.02E-05	1.06E-04	3.167225	GYPC	c("HEME_METABOLISM", "KRAS_SIGNALING_UP")
1.333087	0.014875	4.702212	1.02E-05	1.06E-04	3.166514	NIBAN1	c("CHOLESTEROL_HOMEOSTASIS", "MTORC1_SIGNALING")
-0.71842	-0.05525	-4.7001	1.03E-05	1.07E-04	3.158798	PHF5A	E2F_TARGETS
-1.34008	-0.13788	-4.69219	1.06E-05	1.09E-04	3.129979	DEK	c("E2F_TARGETS", "MYC_TARGETS_V1")
-1.058	-0.14127	-4.67204	1.15E-05	1.17E-04	3.056651	SNRPD1	c("G2M_CHECKPOINT", "MYC_TARGETS_V1")

-1.38852	-0.69564	-4.66354	1.18E-05	1.20E-04	3.025749	CDK13	UV_RESPONSE_DN
2.742032	-0.23813	4.663141	1.19E-05	1.20E-04	3.024303	FHL1	MYOGENESIS
1.504415	-0.10128	4.655369	1.22E-05	1.22E-04	2.996096	DTNA	c("HYPOXIA", "MYOGENESIS")
1.350394	-0.5793	4.654626	1.23E-05	1.22E-04	2.993402	PDE4DIP	MYOGENESIS
-1.04021	-0.09875	-4.6535	1.23E-05	1.22E-04	2.989299	SFPQ	G2M_CHECKPOINT
-1.37706	-0.12725	-4.65118	1.24E-05	1.23E-04	2.980914	HIBCH	c("ADIPOGENESIS", "FATTY_ACID_METABOLISM")
-1.13339	-0.09588	-4.65081	1.24E-05	1.23E-04	2.979564	DNAJB1	UV_RESPONSE_UP
1.051196	0.0095	4.645876	1.27E-05	1.25E-04	2.961679	TCIRG1	OXIDATIVE_PHOSPHORYLATION
-1.23034	-0.20088	-4.64158	1.29E-05	1.26E-04	2.94611	PRDX5	c("BILE_ACID_METABOLISM", "HYPOXIA", "PEROXISOME")
-1.04122	-0.08888	-4.63957	1.30E-05	1.27E-04	2.93882	COX7A2	OXIDATIVE_PHOSPHORYLATION
-0.99498	-0.03225	-4.63202	1.34E-05	1.29E-04	2.911521	ERH	MYC_TARGETS_V1
1.238123	-0.10288	4.631826	1.34E-05	1.29E-04	2.910814	G6PD	c("GLYCOLYSIS", "MTORC1_SIGNALING", "REACTIVE_OXYGEN_SPECIES_PATHWAY")
1.129653	-0.08475	4.623426	1.38E-05	1.32E-04	2.880443	VASP	APICAL_JUNCTION
-1.53867	-0.56937	-4.61818	1.41E-05	1.34E-04	2.861487	ERCC3	c("DNA_REPAIR", "PEROXISOME")
-1.79241	-0.6371	-4.61324	1.44E-05	1.36E-04	2.843663	BAG1	c("ESTROGEN_RESPONSE_EARLY", "ESTROGEN_RESPONSE_LATE", "MYOGENESIS")

-1.51325	-0.16488	-4.61238	1.44E-05	1.36E-04	2.840547	LSR	ESTROGEN_RESPONSE_LATE
1.52201	-0.11125	4.61022	1.45E-05	1.37E-04	2.832767	EHD1	c("COMPLEMENT", "TNFA_SIGNALING_VIA_NFKB")
-1.53194	-0.17263	-4.59825	1.52E-05	1.41E-04	2.789623	SPTBN2	KRAS_SIGNALING_DN
-1.36878	-0.15275	-4.5969	1.53E-05	1.42E-04	2.784749	APEX1	c("FATTY_ACID_METABOLISM", "MYC_TARGETS_V1")
-0.80757	-0.06113	-4.58349	1.61E-05	1.48E-04	2.736491	EWSR1	G2M_CHECKPOINT
-1.15407	-0.08663	-4.5803	1.63E-05	1.50E-04	2.725051	HNRNPA1	MYC_TARGETS_V1
1.435413	-0.21774	4.577272	1.65E-05	1.51E-04	2.714155	GABARAPL2	MYOGENESIS
-1.68825	-0.205	-4.57433	1.67E-05	1.52E-04	2.703602	CYC1	c("ADIPOGENESIS", "MYC_TARGETS_V1", "OXIDATIVE_PHOSPHORYLATION")
4.095202	-2.038	4.572348	1.68E-05	1.53E-04	2.696474	PPBP	KRAS_SIGNALING_UP
-1.60801	-0.59642	-4.57023	1.69E-05	1.54E-04	2.688863	GPHN	ADIPOGENESIS
3.126415	-0.53388	4.562912	1.74E-05	1.57E-04	2.662616	MMP9	c("ALLOGRAFT_REJECTION", "APICAL_JUNCTION", "COAGULATION", "KRAS_SIGNALING_UP")
-0.94338	-0.03763	-4.54659	1.85E-05	1.66E-04	2.604157	TOMM70	c("MYC_TARGETS_V1", "OXIDATIVE_PHOSPHORYLATION")
-1.66366	-0.66662	-4.53411	1.94E-05	1.72E-04	2.559534	CIT	E2F_TARGETS
1.816428	-0.0395	4.515063	2.09E-05	1.83E-04	2.491569	C1QA	c("COAGULATION", "COMPLEMENT")
-1.05944	-0.07375	-4.51038	2.12E-05	1.85E-04	2.474902	NELFB	DNA_REPAIR

-1.58182	-0.594	-4.50361	2.18E-05	1.88E-04	2.450802	YPEL5	HEME_METABOLISM
3.711294	-0.95475	4.501227	2.20E-05	1.89E-04	2.442309	MPO	REACTIVE_OXYGEN_SPECIES_PATHWAY
1.380715	-0.11725	4.497751	2.23E-05	1.91E-04	2.429948	C1S	c("COAGULATION", "COMPLEMENT", "INTERFERON_ALPHA_RESPONSE", "INTERFERON_GAMMA_RESPONSE")
1.445842	0.002567	4.497361	2.23E-05	1.91E-04	2.428561	EPB41L3	KRAS_SIGNALING_UP
-1.08211	-0.10863	-4.48676	2.32E-05	1.97E-04	2.39089	ATP5MG	OXIDATIVE_PHOSPHORYLATION
-1.43064	-0.17988	-4.48569	2.33E-05	1.97E-04	2.387116	SORD	c("ANDROGEN_RESPONSE", "ESTROGEN_RESPONSE_LATE", "MTORC1_SIGNALING", "MYC_TARGETS_V2")
-0.97443	-0.17765	-4.48088	2.37E-05	1.99E-04	2.370044	OSTC	FATTY_ACID_METABOLISM
1.237983	-0.0655	4.480114	2.38E-05	2.00E-04	2.367311	SYK	APICAL_JUNCTION
1.682766	-0.019	4.478184	2.40E-05	2.01E-04	2.360467	TUBB2A	c("TNFA_SIGNALING_VIA_NFKB", "UNFOLDED_PROTEIN_RESPONSE")
-1.77741	-0.18488	-4.47007	2.47E-05	2.06E-04	2.331704	HMGA1	c("E2F_TARGETS", "G2M_CHECKPOINT")
-1.28443	-0.10225	-4.46667	2.50E-05	2.07E-04	2.319669	STRBP	SPERMATOGENESIS
-1.41072	-0.15188	-4.46658	2.50E-05	2.07E-04	2.319332	TBRG4	c("E2F_TARGETS", "MYC_TARGETS_V2")
-1.53272	-0.56179	-4.45134	2.65E-05	2.17E-04	2.265461	WASL	c("APICAL_JUNCTION", "MITOTIC_SPINDLE")
-1.10705	-0.0795	-4.45066	2.66E-05	2.17E-04	2.263048	OXA1L	OXIDATIVE_PHOSPHORYLATION

-1.59869	-0.54582	-4.45064	2.66E-05	2.17E-04	2.262971	RREB1	ADIPOGENESIS
-1.31978	-0.67093	-4.45018	2.66E-05	2.17E-04	2.261361	GTF3C5	c("DNA_REPAIR", "KRAS_SIGNALING_DN")
-1.03707	-0.09375	-4.44146	2.75E-05	2.23E-04	2.230554	LSM4	UNFOLDED_PROTEIN_RESPONSE
1.199483	-0.11375	4.44035	2.76E-05	2.23E-04	2.226639	DPYSL2	HEDGEHOG_SIGNALING
-2.03915	-0.24963	-4.43416	2.83E-05	2.28E-04	2.204808	EPCAM	UV_RESPONSE_UP
-1.16488	-0.0635	-4.42522	2.92E-05	2.33E-04	2.173321	ATP5PF	OXIDATIVE_PHOSPHORYLATION
-0.84241	-0.01875	-4.41921	2.99E-05	2.38E-04	2.152156	MTREX	UNFOLDED_PROTEIN_RESPONSE
2.462691	-0.33581	4.418128	3.00E-05	2.38E-04	2.148357	ZAP70	ALLOGRAFT_REJECTION
-0.95872	-0.09138	-4.41767	3.01E-05	2.38E-04	2.146754	ZC3H14	SPERMATOGENESIS
0.907116	0.00375	4.413623	3.05E-05	2.41E-04	2.132512	ADD1	c("APOPTOSIS", "HEME_METABOLISM")
-1.04708	-0.06	-4.40547	3.15E-05	2.46E-04	2.10387	BANF1	UNFOLDED_PROTEIN_RESPONSE
-2.45027	-0.3295	-4.40293	3.18E-05	2.48E-04	2.094956	MAL2	CHOLESTEROL_HOMEOSTASIS
-0.88986	-0.09313	-4.39752	3.24E-05	2.51E-04	2.075975	XRCC6	c("ANDROGEN_RESPONSE", "E2F_TARGETS", "MYC_TARGETS_V1")
1.978236	-0.43433	4.394262	3.28E-05	2.54E-04	2.064545	ISG20	c("APOPTOSIS", "ESTROGEN_RESPONSE_LATE", "GLYCOLYSIS", "HYPOXIA", "INTERFERON_ALPHA_RESPONSE", "INTERFERON_GAMMA_RESPONSE")

2.861179	-0.40333	4.387768	3.36E-05	2.59E-04	2.041788	CD36	c("ADIPOGENESIS", "COMPLEMENT", "FATTY_ACID_METABOLISM", "IL6_JAK_STAT3_SIGNALING", "MYOGENESIS", "XENOBIOTIC_METABOLISM")
-1.09853	-0.14713	-4.38708	3.37E-05	2.59E-04	2.039386	PMVK	CHOLESTEROL_HOMEOSTASIS
1.401645	-0.11488	4.386329	3.38E-05	2.59E-04	2.036749	MSN	APICAL_JUNCTION
-0.89225	-0.0725	-4.37955	3.47E-05	2.64E-04	2.013013	CETN2	DNA_REPAIR
-1.25417	-0.11825	-4.37336	3.55E-05	2.70E-04	1.991388	NUP153	E2F_TARGETS
-0.95103	-0.10838	-4.36906	3.60E-05	2.73E-04	1.976334	XPO1	c("E2F_TARGETS", "G2M_CHECKPOINT", "MYC_TARGETS_V1")
-0.96323	-0.01363	-4.36727	3.63E-05	2.75E-04	1.97008	NCBP1	MYC_TARGETS_V1
1.842582	-0.24138	4.364976	3.66E-05	2.77E-04	1.962081	FBP1	c("HYPOXIA", "XENOBIOTIC_METABOLISM")
-1.54307	-0.20438	-4.36108	3.71E-05	2.79E-04	1.948466	MCM6	c("E2F_TARGETS", "G2M_CHECKPOINT", "MYC_TARGETS_V1")
1.915318	0.037375	4.357947	3.76E-05	2.82E-04	1.93755	C1QC	COMPLEMENT
-1.2471	-0.08013	-4.34841	3.89E-05	2.91E-04	1.90432	LONP1	XENOBIOTIC_METABOLISM
-2.01485	-0.46689	-4.34712	3.91E-05	2.92E-04	1.899816	SDHD	c("FATTY_ACID_METABOLISM", "OXIDATIVE_PHOSPHORYLATION")
-1.12007	-0.14738	-4.34161	3.99E-05	2.98E-04	1.880647	SRP9	PANCREAS_BETA_CELLS
-1.31749	-0.89509	-4.33737	4.05E-05	3.01E-04	1.865886	GNAI1	c("APICAL_JUNCTION", "CHOLESTEROL_HOMEOSTASIS", "WNT_BETA_CATENIN_SIGNALING")

-0.895	-0.0595	-4.32995	4.17E-05	3.07E-04	1.840098	RBM14	G2M_CHECKPOINT
-1.03066	-0.0885	-4.32424	4.26E-05	3.11E-04	1.820256	CDC5L	UV_RESPONSE_UP
1.286949	-0.02113	4.321446	4.30E-05	3.14E-04	1.810558	PRDX2	c("HEME_METABOLISM", "REACTIVE_OXYGEN_SPECIES_PATHWAY")
3.039933	-0.49357	4.318014	4.35E-05	3.17E-04	1.798652	MMP8	c("COAGULATION", "COMPLEMENT")
2.816686	-0.386	4.316619	4.38E-05	3.17E-04	1.793816	CAVIN1	c("ADIPOGENESIS", "HYPOXIA")
-0.78547	-0.06213	-4.31623	4.38E-05	3.17E-04	1.792464	RAE1	DNA_REPAIR
-1.0137	-0.09813	-4.31349	4.43E-05	3.19E-04	1.782963	SSB	MYC_TARGETS_V1
1.306078	-0.23694	4.311602	4.46E-05	3.21E-04	1.776425	HS2ST1	GLYCOLYSIS
-1.61564	-0.62142	-4.30866	4.51E-05	3.24E-04	1.766232	AUTS2	INTERFERON_GAMMA_RESPONSE
-1.04954	-0.107	-4.295	4.74E-05	3.39E-04	1.718975	NME1	c("ALLOGRAFT_REJECTION", "DNA_REPAIR", "E2F_TARGETS", "MYC_TARGETS_V1")
-1.00618	-0.09863	-4.2752	5.10E-05	3.61E-04	1.650617	DHX15	MYC_TARGETS_V1
-1.01763	-0.04511	-4.26351	5.32E-05	3.74E-04	1.610355	EXOSC7	MYC_TARGETS_V1
-1.41308	-0.17813	-4.2516	5.56E-05	3.86E-04	1.569407	C1QBP	MYC_TARGETS_V1
-0.97192	-0.11038	-4.24373	5.72E-05	3.96E-04	1.542405	MRE11	E2F_TARGETS
0.859268	-0.09925	4.234464	5.92E-05	4.08E-04	1.510632	TPM3	MYOGENESIS

-1.6059	-0.57692	-4.23034	6.01E-05	4.13E-04	1.496515	SRXN1	REACTIVE_OXYGEN_SPECIES_PATHWAY
-1.1067	-0.05575	-4.22486	6.13E-05	4.19E-04	1.477757	MCCC1	ADIPOGENESIS
-1.16215	-0.16075	-4.22259	6.18E-05	4.21E-04	1.470001	RNMT	DNA_REPAIR
-1.48027	-0.62397	-4.216	6.33E-05	4.31E-04	1.447443	CNOT6	UNFOLDED_PROTEIN_RESPONSE
-1.1524	-0.09375	-4.21007	6.47E-05	4.39E-04	1.427198	SUCLG1	c("ADIPOGENESIS", "FATTY_ACID_METABOLISM", "OXIDATIVE_PHOSPHORYLATION")
0.787515	-0.00313	4.205947	6.57E-05	4.44E-04	1.413139	CAPZB	MITOTIC_SPINDLE
-1.33159	-0.10738	-4.19877	6.74E-05	4.54E-04	1.388667	DDX21	c("MYC_TARGETS_V1", "UV_RESPONSE_UP")
-0.73124	-0.024	-4.18724	7.03E-05	4.70E-04	1.34942	POLR2E	DNA_REPAIR
-1.63684	-0.62827	-4.18258	7.15E-05	4.76E-04	1.333559	LRIG1	c("ESTROGEN_RESPONSE_EARLY", "IL2_STAT5_SIGNALING")
1.141512	-0.04088	4.174513	7.36E-05	4.86E-04	1.306162	NFKB1	c("INFLAMMATORY_RESPONSE", "INTERFERON_GAMMA_RESPONSE", "TNFA_SIGNALING_VIA_NFKB", "UV_RESPONSE_DN")
-0.71273	-0.016	-4.17057	7.47E-05	4.92E-04	1.292771	TARDBP	MYC_TARGETS_V1
-1.4236	-0.21313	-4.16831	7.53E-05	4.95E-04	1.285115	GPD2	c("ADIPOGENESIS", "COMPLEMENT", "FATTY_ACID_METABOLISM")
-1.7485	-0.39421	-4.16797	7.54E-05	4.95E-04	1.283972	POLD2	c("E2F_TARGETS", "MYC_TARGETS_V1")
-0.97687	-0.09038	-4.16457	7.63E-05	4.98E-04	1.272431	COX4I1	OXIDATIVE_PHOSPHORYLATION

-0.80128	-0.0057	-4.15681	7.85E-05	5.06E-04	1.246139	GEMIN4	c("MITOTIC_SPINDLE", "UNFOLDED_PROTEIN_RESPONSE")
1.824538	-0.04975	4.151587	8.00E-05	5.14E-04	1.228463	VWF	c("APICAL_JUNCTION", "COAGULATION")
1.206853	-0.09588	4.151196	8.01E-05	5.14E-04	1.227143	CAST	HEME_METABOLISM
1.57754	-0.56541	4.148621	8.08E-05	5.18E-04	1.218434	PSMG1	c("MTORC1_SIGNALING", "SPERMATOGENESIS")
-1.55405	-0.26	-4.14799	8.10E-05	5.19E-04	1.216301	ZNF185	ESTROGEN_RESPONSE_EARLY
-2.14576	-0.24113	-4.13437	8.51E-05	5.42E-04	1.170283	UNG	c("E2F_TARGETS", "MTORC1_SIGNALING", "MYC_TARGETS_V2")
-1.00788	-0.04113	-4.13104	8.61E-05	5.46E-04	1.159065	TAX1BP3	P53_PATHWAY
-1.06073	-0.11813	-4.13082	8.62E-05	5.46E-04	1.158317	TOMM40	MTORC1_SIGNALING
1.415604	-0.22182	4.129672	8.65E-05	5.47E-04	1.154452	AGPAT3	ADIPOGENESIS
1.119236	-0.2752	4.123141	8.86E-05	5.58E-04	1.132443	CCNK	P53_PATHWAY
-1.33476	-0.73878	-4.12228	8.89E-05	5.59E-04	1.129536	IKBKG	APICAL_JUNCTION
-1.30697	-0.13409	-4.11822	9.02E-05	5.66E-04	1.115873	MRPL9	MYC_TARGETS_V1
-0.95766	-0.06813	-4.11489	9.13E-05	5.72E-04	1.104686	CSTF2	MYC_TARGETS_V1
-2.46669	-0.23375	-4.11306	9.19E-05	5.74E-04	1.098503	ERBB2	c("APOPTOSIS", "UV_RESPONSE_DN")
1.472031	0.016625	4.112728	9.20E-05	5.74E-04	1.0974	GNG2	COMPLEMENT
1.129095	-0.03963	4.105566	9.44E-05	5.85E-04	1.073332	LYN	c("ALLOGRAFT_REJECTION", "APICAL_SURFACE", "COMPLEMENT",

							"INFLAMMATORY_RESPONSE", "UV_RESPONSE_UP")
-1.5594	-0.59618	-4.10351	9.51E-05	5.89E-04	1.066417	SOS1	MITOTIC_SPINDLE
-0.56171	-0.04538	-4.1018	9.57E-05	5.90E-04	1.060683	PPP1CA	c("PI3K_AKT_MTOR_SIGNALING", "TGF_BETA_SIGNALING")
-1.15883	-0.10263	-4.10039	9.61E-05	5.93E-04	1.055952	PDHB	c("FATTY_ACID_METABOLISM", "OXIDATIVE_PHOSPHORYLATION")
-1.3912	-0.67541	-4.09811	9.69E-05	5.95E-04	1.048321	POLD4	DNA_REPAIR
-0.98108	-0.13575	-4.09306	9.87E-05	6.02E-04	1.03136	UBA2	MYC_TARGETS_V1
-0.96491	-0.11075	-4.09227	9.90E-05	6.03E-04	1.028728	G3BP1	c("G2M_CHECKPOINT", "MYC_TARGETS_V1")
-1.55518	-0.66753	-4.09134	9.93E-05	6.03E-04	1.025616	DPYSL4	c("GLYCOLYSIS", "HYPOXIA")
-1.61614	-0.67503	-4.09097	9.94E-05	6.03E-04	1.02437	CDH3	APICAL_JUNCTION
0.840012	0.028375	4.090256	9.97E-05	6.03E-04	1.021975	AKAP13	MITOTIC_SPINDLE
-0.99328	-0.08388	-4.08879	1.00E-04	6.05E-04	1.017052	CDK9	TGF_BETA_SIGNALING
-1.04997	-0.10513	-4.08781	1.01E-04	6.06E-04	1.013784	PDHA1	c("FATTY_ACID_METABOLISM", "OXIDATIVE_PHOSPHORYLATION")
1.189126	-0.09925	4.08476	1.02E-04	6.08E-04	1.003567	BIN1	c("MITOTIC_SPINDLE", "MYOGENESIS")
-0.69796	-0.064	-4.08459	1.02E-04	6.08E-04	1.002992	PCBP1	MYC_TARGETS_V1
-1.07676	-0.07063	-4.07851	1.04E-04	6.18E-04	0.982658	ATP5PB	OXIDATIVE_PHOSPHORYLATION
0.98247	-0.00325	4.076429	1.05E-04	6.21E-04	0.975698	SPTBN1	c("MITOTIC_SPINDLE", "TGF_BETA_SIGNALING")

-1.10033	-0.07913	-4.06922	1.07E-04	6.37E-04	0.951609	UQCRC1	c("ADIPOGENESIS", "OXIDATIVE_PHOSPHORYLATION")
-1.12254	-0.11338	-4.06249	1.10E-04	6.48E-04	0.929155	CPOX	c("FATTY_ACID_METABOLISM", "HEME_METABOLISM")
-1.26934	-0.13838	-4.06105	1.11E-04	6.51E-04	0.924349	AFG3L2	OXIDATIVE_PHOSPHORYLATION
-1.45188	-0.18588	-4.06024	1.11E-04	6.52E-04	0.921655	DEPTOR	ESTROGEN_RESPONSE_EARLY
-1.05497	-0.141	-4.04589	1.17E-04	6.80E-04	0.873841	GOT2	c("GLYCOLYSIS", "MYC_TARGETS_V1", "OXIDATIVE_PHOSPHORYLATION")
-1.4773	-0.62067	-4.04177	1.19E-04	6.89E-04	0.860157	EXOSC8	E2F_TARGETS
-0.9316	-0.14738	-4.03412	1.22E-04	7.03E-04	0.83473	CBX3	c("MYC_TARGETS_V1", "MYC_TARGETS_V2")
-1.56522	-0.19013	-4.03193	1.23E-04	7.06E-04	0.827485	SLC25A13	GLYCOLYSIS
0.729945	-0.01913	4.030319	1.23E-04	7.08E-04	0.822123	NAPA	PROTEIN_SECRETION
-0.78198	-0.04813	-4.02905	1.24E-04	7.10E-04	0.8179	XRCC5	ANDROGEN_RESPONSE
1.666919	-0.12763	4.024378	0.000126	7.20E-04	0.802418	CD44	c("APOPTOSIS", "EPITHELIAL_MESENCHYMAL_TRANSITION", "ESTROGEN_RESPONSE_EARLY", "ESTROGEN_RESPONSE_LATE", "GLYCOLYSIS", "IL2_STAT5_SIGNALING", "IL6_JAK_STAT3_SIGNALING", "TNFA_SIGNALING_VIA_NFKB")
-0.73666	-0.04663	-4.02424	1.26E-04	7.20E-04	0.801959	TIAL1	APICAL_JUNCTION
-0.78034	-0.04038	-4.01974	1.28E-04	7.30E-04	0.787047	SAMM50	ADIPOGENESIS

-1.26681	-0.21388	-4.01566	1.30E-04	7.37E-04	0.773536	HEXIM1	P53_PATHWAY
0.566354	0.002375	4.014572	1.31E-04	7.38E-04	0.769939	DCTN1	UNFOLDED_PROTEIN_RESPONSE
0.808551	-0.03088	4.014553	1.31E-04	7.38E-04	0.769878	BAG3	UNFOLDED_PROTEIN_RESPONSE
-1.78785	-0.43494	-4.01446	1.31E-04	7.38E-04	0.769565	TJP3	c("ESTROGEN_RESPONSE_EARLY", "ESTROGEN_RESPONSE_LATE")
1.588769	-0.20038	4.013102	1.31E-04	7.39E-04	0.765074	ARHGAP4	MITOTIC_SPINDLE
-1.56655	-0.47844	-4.01097	1.32E-04	7.44E-04	0.758016	CASP8	c("APOPTOSIS", "INTERFERON_ALPHA_RESPONSE", "INTERFERON_GAMMA_RESPONSE")
-1.10384	-0.04413	-4.01045	1.32E-04	7.44E-04	0.756308	PNPT1	c("INTERFERON_ALPHA_RESPONSE", "INTERFERON_GAMMA_RESPONSE")
1.212073	-0.26989	4.007656	1.34E-04	7.50E-04	0.747064	ACSS2	CHOLESTEROL_HOMEOSTASIS
1.802581	-0.31963	4.003173	1.36E-04	7.59E-04	0.732249	PSMB10	c("ALLOGRAFT_REJECTION", "INTERFERON_GAMMA_RESPONSE", "XENOBIOTIC_METABOLISM")
-1.00836	-0.0585	-3.99741	1.39E-04	7.72E-04	0.713228	CLPB	SPERMATOGENESIS
1.524732	-0.04816	3.991159	1.42E-04	7.86E-04	0.692606	CD38	c("APOPTOSIS", "IL6_JAK_STAT3_SIGNALING", "INTERFERON_GAMMA_RESPONSE")
-0.8257	-0.1165	-3.98825	1.43E-04	7.94E-04	0.683024	EDF1	DNA_REPAIR
0.684775	0.055125	3.985612	1.45E-04	8.00E-04	0.674325	PSMD9	HEME_METABOLISM
-1.39934	-0.169	-3.97991	1.48E-04	8.13E-04	0.655571	MCM7	c("E2F_TARGETS", "EPITHELIAL_MESENCHYMAL_TRANSITION", "MYC_TARGETS_V1")

-1.49458	-0.40814	-3.97762	1.49E-04	8.18E-04	0.648024	PEX6	c("BILE_ACID_METABOLISM", "PEROXISOME")
-0.92886	-0.11413	-3.97633	1.49E-04	8.21E-04	0.643787	GMPS	c("HEME_METABOLISM", "MTORC1_SIGNALING")
-0.82976	-0.0535	-3.96973	1.53E-04	8.38E-04	0.622083	LSM7	MYC_TARGETS_V1
0.973951	-0.03525	3.969596	1.53E-04	8.38E-04	0.621647	TRIM21	c("INTERFERON_ALPHA_RESPONSE", "INTERFERON_GAMMA_RESPONSE")
-0.94998	-0.11888	-3.96196	1.57E-04	8.57E-04	0.59657	UBE2I	ANDROGEN_RESPONSE
-1.55647	-0.43699	-3.95971	1.58E-04	8.63E-04	0.589209	IDUA	c("GLYCOLYSIS", "KRAS_SIGNALING_DN")
-1.78838	-0.20113	-3.95122	1.63E-04	8.85E-04	0.561377	PKP2	GLYCOLYSIS
1.0335	-0.0115	3.942473	1.68E-04	9.08E-04	0.53276	RAP2B	P53_PATHWAY
-0.73279	-0.10825	-3.94114	1.69E-04	9.11E-04	0.52839	SYNCRIP	c("E2F_TARGETS", "G2M_CHECKPOINT", "MYC_TARGETS_V1")
-1.61764	-0.26327	-3.93828	1.71E-04	9.19E-04	0.519065	PDK2	KRAS_SIGNALING_DN
1.388021	-0.5477	3.935246	1.73E-04	9.28E-04	0.509148	SP110	c("INTERFERON_ALPHA_RESPONSE", "INTERFERON_GAMMA_RESPONSE")
1.87941	-0.37909	3.9285	1.77E-04	9.44E-04	0.487131	LTBP1	UV_RESPONSE_DN
-1.68257	-0.309	-3.92107	1.81E-04	9.65E-04	0.462916	EPS8L2	P53_PATHWAY
1.056543	-0.075	3.919647	1.82E-04	9.68E-04	0.458275	GCLC	c("GLYCOLYSIS", "HEME_METABOLISM", "MTORC1_SIGNALING", "REACTIVE_OXYGEN_SPECIES_PATHWAY", "XENOBIOTIC_METABOLISM")

-1.17034	-0.1455	-3.91253	1.87E-04	9.89E-04	0.435112	SSRP1	c("DNA_REPAIR", "E2F_TARGETS")
1.112316	0.0165	3.911861	1.87E-04	9.89E-04	0.432936	MARCKS	c("G2M_CHECKPOINT", "MITOTIC_SPINDLE", "TNFA_SIGNALING_VIA_NFKB")
-0.85676	-0.14332	-3.90368	1.93E-04	0.00101	0.406362	TIMM9	OXIDATIVE_PHOSPHORYLATION
1.562248	-0.09998	3.898636	1.96E-04	0.001023	0.389972	ITIH3	KRAS_SIGNALING_DN
-0.82138	-0.04996	-3.89698	1.97E-04	0.001028	0.384601	GRWD1	MYC_TARGETS_V2
-1.1901	-0.18813	-3.88921	2.03E-04	0.00105	0.359409	COX17	c("DNA_REPAIR", "OXIDATIVE_PHOSPHORYLATION")
-1.42275	-0.70408	-3.88806	2.04E-04	0.001052	0.355688	SELENOW	UV_RESPONSE_UP
0.885292	-0.081	3.881257	2.08E-04	0.00107	0.333666	MYO9B	MITOTIC_SPINDLE
-0.93842	-0.02328	-3.88102	2.09E-04	0.00107	0.332904	RBM5	HEME_METABOLISM
1.17653	-0.097	3.876782	2.12E-04	0.001081	0.319194	FABP5	c("CHOLESTEROL_HOMEOSTASIS", "ESTROGEN_RESPONSE_LATE")
-1.13045	-0.11288	-3.87676	2.12E-04	0.001081	0.319132	PMPCA	OXIDATIVE_PHOSPHORYLATION
1.480315	-0.36374	3.874055	2.14E-04	0.001091	0.310381	IL18	c("ALLOGRAFT_REJECTION", "APOPTOSIS", "INFLAMMATORY_RESPONSE", "TNFA_SIGNALING_VIA_NFKB")
-1.40638	-0.75806	-3.87208	2.15E-04	0.001097	0.304001	TRIM33	TGF_BETA_SIGNALING
-1.4585	-0.4541	-3.87039	2.16E-04	0.001102	0.298557	PLXNB1	ESTROGEN_RESPONSE_LATE
1.298346	-0.16404	3.869548	2.17E-04	0.001104	0.295824	NIPBL	UV_RESPONSE_DN

-1.57638	-0.284	-3.86238	2.22E-04	0.001125	0.272702	HMGB3	c("E2F_TARGETS", "G2M_CHECKPOINT")
-0.76554	-0.087	-3.86224	2.23E-04	0.001125	0.272237	RHOT2	c("APOPTOSIS", "MITOTIC_SPINDLE", "OXIDATIVE_PHOSPHORYLATION")
-0.96316	-0.15525	-3.85392	2.29E-04	0.001155	0.245441	SET	MYC_TARGETS_V1
-1.09484	-0.1145	-3.85347	2.29E-04	0.001156	0.243978	OPA1	OXIDATIVE_PHOSPHORYLATION
-0.8198	-0.06863	-3.85265	2.30E-04	0.001157	0.241343	CD2AP	MITOTIC_SPINDLE
-0.89063	-0.15063	-3.85223	2.30E-04	0.001157	0.240013	PABPC4	MYC_TARGETS_V1
-1.28265	-0.60187	-3.84302	2.38E-04	0.001189	0.210399	NF2	c("APICAL_JUNCTION", "SPERMATOGENESIS")
1.862463	-0.03188	3.836127	2.44E-04	0.001212	0.188251	A2M	c("COAGULATION", "IL6_JAK_STAT3_SIGNALING")
-0.73618	-0.0245	-3.83105	2.48E-04	0.001231	0.171971	STAG1	c("E2F_TARGETS", "G2M_CHECKPOINT")
1.28353	-0.00925	3.828697	2.50E-04	0.00124	0.164421	DAB2	c("EPITHELIAL_MESENCHYMAL_TRANSITION", "UV_RESPONSE_DN")
-1.41535	-0.73007	-3.82709	2.51E-04	0.001245	0.159263	F9	COAGULATION
-0.94031	-0.093	-3.82534	0.000253	0.00125	0.153672	IMMT	c("ADIPOGENESIS", "MTORC1_SIGNALING", "OXIDATIVE_PHOSPHORYLATION")
-1.04355	-0.08775	-3.82268	2.55E-04	0.001257	0.145146	NFS1	XENOBIOTIC_METABOLISM
0.986749	-0.08988	3.813573	2.63E-04	0.001293	0.116017	RTN1	HEDGEHOG_SIGNALING
0.862113	-0.054	3.808516	2.68E-04	0.001314	0.099862	ACTR3	c("MTORC1_SIGNALING", "PI3K_AKT_MTOR_SIGNALING")

-1.27371	-0.71238	-3.8021	2.74E-04	0.00134	0.079399	RBPJ	c("HYPOXIA", "WNT_BETA_CATENIN_SIGNALING")
-1.01071	-0.05375	-3.80128	2.75E-04	0.001343	0.076766	HTRA2	c("HEME_METABOLISM", "OXIDATIVE_PHOSPHORYLATION")
0.799513	-0.02014	3.800249	2.76E-04	0.001345	0.073483	GSK3B	c("MTORC1_SIGNALING", "PI3K_AKT_MTOR_SIGNALING")
-0.96416	-0.05463	-3.79605	2.80E-04	0.001357	0.060102	TOP1	c("G2M_CHECKPOINT", "HEME_METABOLISM")
2.402982	-0.3595	3.792726	2.83E-04	0.00137	0.049513	CAV1	c("APOPTOSIS", "ESTROGEN_RESPONSE_LATE", "HYPOXIA", "UV_RESPONSE_DN")
-0.61687	-0.07363	-3.7924	2.83E-04	0.00137	0.048489	RPLP0	MYC_TARGETS_V1
1.349765	-0.16245	3.792273	2.83E-04	0.00137	0.048072	STBD1	HYPOXIA
2.591862	-0.406	3.79176	2.84E-04	0.001371	0.04644	CA2	c("COMPLEMENT", "ESTROGEN_RESPONSE_LATE", "FATTY_ACID_METABOLISM", "HEME_METABOLISM", "IL2_STAT5_SIGNALING", "KRAS_SIGNALING_UP", "UV_RESPONSE_UP", "XENOBIOTIC_METABOLISM")
1.81298	-0.20585	3.789749	2.86E-04	0.001378	0.040037	ITGA7	c("ADIPOGENESIS", "MYOGENESIS")
-0.86978	-0.07771	-3.78771	2.88E-04	0.001384	0.033545	AKT1S1	PI3K_AKT_MTOR_SIGNALING
1.313631	-0.0975	3.781718	2.94E-04	0.001406	0.014499	FKBP5	c("ANDROGEN_RESPONSE", "ESTROGEN_RESPONSE_EARLY", "ESTROGEN_RESPONSE_LATE")
-0.80831	-0.05363	-3.7794	2.96E-04	0.001415	0.007135	PTRH2	IL2_STAT5_SIGNALING
1.020285	-0.03488	3.779128	2.96E-04	0.001415	0.006271	PYGL	GLYCOLYSIS

-1.22615	-0.82138	-3.77672	2.99E-04	0.001424	-0.00138	CSF1	c("ALLOGRAFT_REJECTION", "IL2_STAT5_SIGNALING", "IL6_JAK_STAT3_SIGNALING", "INFLAMMATORY_RESPONSE", "INTERFERON_ALPHA_RESPONSE", "TNFA_SIGNALING_VIA_NFKB")
1.211091	-0.01175	3.762122	3.14E-04	0.001488	-0.04766	LRP1	c("COAGULATION", "COMPLEMENT", "EPITHELIAL_MESENCHYMAL_TRANSITION")
-1.51858	-0.41224	-3.75981	3.17E-04	0.001495	-0.05496	TFB2M	MYC_TARGETS_V2
-0.97245	-0.069	-3.75837	3.18E-04	0.001502	-0.05952	ATP5F1B	OXIDATIVE_PHOSPHORYLATION
0.908186	-0.01413	3.754522	3.22E-04	0.001517	-0.0717	GSN	c("APOPTOSIS", "COAGULATION", "MITOTIC_SPINDLE", "MYOGENESIS")
-1.08691	-0.1695	-3.75429	3.23E-04	0.001517	-0.07245	NUMA1	c("G2M_CHECKPOINT", "MITOTIC_SPINDLE")
1.946724	-0.1987	3.75314	3.24E-04	0.001522	-0.07607	C4BPB	c("COMPLEMENT", "UV_RESPONSE_UP")
1.371589	-0.18083	3.751101	3.26E-04	0.00153	-0.08251	SAMD9	INTERFERON_ALPHA_RESPONSE
3.021039	-0.54713	3.748948	3.28E-04	0.001539	-0.08931	DCN	c("APOPTOSIS", "EPITHELIAL_MESENCHYMAL_TRANSITION", "GLYCOLYSIS", "HYPOXIA")
-0.68119	0.02896	-3.7489	3.29E-04	0.001539	-0.08947	PRKAB1	P53_PATHWAY
-1.1196	-0.07125	-3.74698	3.31E-04	0.001547	-0.09552	TIMM8B	OXIDATIVE_PHOSPHORYLATION
-1.22527	-0.10163	-3.74099	3.37E-04	0.001567	-0.11444	SLC25A10	c("ADIPOGENESIS", "GLYCOLYSIS")
-0.72244	-0.06038	-3.73903	3.40E-04	0.001576	-0.12062	RAN	c("E2F_TARGETS", "MYC_TARGETS_V1")

1.185186	-0.065	3.73864	3.40E-04	0.001577	-0.12184	UBA7	INTERFERON_ALPHA_RESPONSE
-0.94724	-0.07438	-3.73492	3.44E-04	0.001594	-0.13355	NDUFS8	OXIDATIVE_PHOSPHORYLATION
1.614816	-0.0622	3.731129	3.49E-04	0.00161	-0.1455	FCER1G	c("COMPLEMENT", "KRAS_SIGNALING_UP")
1.293087	-0.15363	3.72992	3.50E-04	0.001614	-0.1493	CSK	ALLOGRAFT_REJECTION
-1.24512	-0.196	-3.72415	3.57E-04	0.00164	-0.16745	PPA1	MTORC1_SIGNALING
-1.4448	-0.23975	-3.71011	3.75E-04	0.001704	-0.21154	ITGA3	APICAL_JUNCTION
0.889924	-0.08875	3.706243	3.80E-04	0.001724	-0.22365	ARPC2	APICAL_JUNCTION
-0.99482	-0.01563	-3.70558	3.80E-04	0.001726	-0.22573	SUPV3L1	c("MYC_TARGETS_V2", "OXIDATIVE_PHOSPHORYLATION")
-1.42238	-0.37059	-3.70425	3.82E-04	0.001727	-0.2299	MRPL40	DNA_REPAIR
1.079145	-0.22282	3.704225	3.82E-04	0.001727	-0.22997	SEC11A	c("MTORC1_SIGNALING", "PANCREAS_BETA_CELLS", "UNFOLDED_PROTEIN_RESPONSE")
-1.27327	-0.19063	-3.70418	3.82E-04	0.001727	-0.23012	MCM3	c("E2F_TARGETS", "G2M_CHECKPOINT")
-1.42335	-0.21375	-3.69854	3.90E-04	0.001753	-0.24777	MCM2	c("E2F_TARGETS", "G2M_CHECKPOINT", "MTORC1_SIGNALING", "MYC_TARGETS_V1")
-0.73967	0.006125	-3.69514	3.94E-04	0.001767	-0.25839	SNRPG	MYC_TARGETS_V1
-0.60496	-0.04713	-3.6856	4.07E-04	0.001805	-0.28819	RPS14	UNFOLDED_PROTEIN_RESPONSE
-1.30365	-0.58943	-3.67664	4.19E-04	0.001852	-0.31611	ATAD2	E2F_TARGETS

1.031789	-0.10575	3.673808	4.23E-04	0.001865	-0.32495	FLNA	c("ALLOGRAFT_REJECTION", "EPITHELIAL_MESENCHYMAL_TRANSITION", "MITOTIC_SPINDLE")
-1.19106	-0.15213	-3.66994	4.29E-04	0.001888	-0.33699	MCM4	c("E2F_TARGETS", "MTORC1_SIGNALING", "MYC_TARGETS_V1", "MYC_TARGETS_V2")
-0.98421	-0.10913	-3.66277	4.39E-04	0.001922	-0.35928	NPM1	c("ALLOGRAFT_REJECTION", "MYC_TARGETS_V1", "MYC_TARGETS_V2", "UNFOLDED_PROTEIN_RESPONSE")
-0.83633	-0.07513	-3.65808	4.46E-04	0.001944	-0.37385	UMPS	c("DNA_REPAIR", "IL2_STAT5_SIGNALING")
2.192635	-0.38625	3.658047	4.46E-04	0.001944	-0.37394	CD2	c("ALLOGRAFT_REJECTION", "APOPTOSIS")
-0.60111	-0.078	-3.65323	4.54E-04	0.001971	-0.38887	RACK1	c("MYC_TARGETS_V1", "P53_PATHWAY")
2.366873	-0.466	3.652137	4.55E-04	0.001975	-0.39228	PGLYRP1	ANGIOGENESIS
-1.4245	-0.19725	-3.65	4.59E-04	0.001986	-0.3989	MACROH2A2	KRAS_SIGNALING_DN
1.091096	-0.20854	3.645386	4.66E-04	0.002009	-0.41319	GALK2	GLYCOLYSIS
1.052168	-0.3135	3.634147	4.84E-04	0.002076	-0.44795	PGK2	SPERMATOGENESIS
-1.23477	-0.02063	-3.63362	4.85E-04	0.002078	-0.44957	SLC25A4	c("OXIDATIVE_PHOSPHORYLATION", "PEROXISOME", "UV_RESPONSE_UP")
-0.90264	-0.06215	-3.63148	4.88E-04	0.002088	-0.45619	MRPS11	OXIDATIVE_PHOSPHORYLATION
-1.60938	-0.5378	-3.62977	4.91E-04	0.002098	-0.46148	CYTH2	MITOTIC_SPINDLE
0.660141	-0.037	3.626559	4.96E-04	0.002113	-0.47138	RPS6KA3	c("ANDROGEN_RESPONSE", "PI3K_AKT_MTOR_SIGNALING", "PROTEIN_SECRETION")

-0.89758	-0.08488	-3.62624	4.97E-04	0.002113	-0.47235	COX5A	c("MYC_TARGETS_V1", "OXIDATIVE_PHOSPHORYLATION")
-0.89406	-0.051	-3.6261	4.97E-04	0.002113	-0.4728	ATP5F1A	OXIDATIVE_PHOSPHORYLATION
-0.82667	-0.06463	-3.62112	5.05E-04	0.002139	-0.48815	NDUFS2	c("OXIDATIVE_PHOSPHORYLATION", "REACTIVE_OXYGEN_SPECIES_PATHWAY")
-0.6209	-0.14038	-3.61671	5.13E-04	0.002167	-0.50174	RPL36	P53_PATHWAY
0.970245	-0.13613	3.611348	5.22E-04	0.002202	-0.51823	SERPINB6	IL2_STAT5_SIGNALING
-0.98105	-0.05625	-3.60503	5.33E-04	0.00224	-0.53764	UQCRC2	OXIDATIVE_PHOSPHORYLATION
-1.1244	-0.20375	-3.596	5.49E-04	0.002298	-0.56536	CDK1	c("E2F_TARGETS", "G2M_CHECKPOINT", "GLYCOLYSIS", "MITOTIC_SPINDLE", "PI3K_AKT_MTOR_SIGNALING", "SPERMATOGENESIS")
1.77116	-0.33088	3.593206	5.54E-04	0.002314	-0.57392	SULT1A1	c("ADIPOGENESIS", "UV_RESPONSE_UP")
-0.9662	-0.1185	-3.58599	5.68E-04	0.00236	-0.59601	CUL4A	G2M_CHECKPOINT
1.163144	-0.06075	3.584332	5.71E-04	0.002372	-0.60109	GLIPR2	IL2_STAT5_SIGNALING
0.7694	-0.06675	3.583441	5.72E-04	0.002376	-0.60382	ACTB	APICAL_JUNCTION
-1.28065	-0.60668	-3.5794	5.80E-04	0.002399	-0.61617	SELENOS	c("INFLAMMATORY_RESPONSE", "REACTIVE_OXYGEN_SPECIES_PATHWAY")
-0.78188	-0.09613	-3.5786	5.82E-04	0.002404	-0.61861	RUVBL2	MYC_TARGETS_V1
1.500986	-0.1925	3.573104	5.92E-04	0.002446	-0.6354	IFI30	c("INTERFERON_ALPHA_RESPONSE", "INTERFERON_GAMMA_RESPON

							SE", "MTORC1_SIGNALING", "P53_PATHWAY")
-1.25567	-0.14788	-3.57276	5.93E-04	0.002447	-0.63643	MCM5	c("E2F_TARGETS", "G2M_CHECKPOINT", "MYC_TARGETS_V1", "MYC_TARGETS_V2")
-0.85796	-0.09963	-3.56818	6.02E-04	0.002482	-0.65041	SRP14	PANCREAS_BETA_CELLS
2.024276	-0.11538	3.562158	6.14E-04	0.002522	-0.66877	SOD3	MYOGENESIS
-0.74476	-0.09175	-3.56105	6.16E-04	0.00253	-0.67213	DARS1	ALLOGRAFT_REJECTION
2.955618	-0.78463	3.557329	6.24E-04	0.002557	-0.68346	LTF	c("COMPLEMENT", "ESTROGEN_RESPONSE_LATE")
-1.00005	-0.10363	-3.55454	6.30E-04	0.002575	-0.69194	IDH3B	c("FATTY_ACID_METABOLISM", "OXIDATIVE_PHOSPHORYLATION")
-0.89422	-0.06763	-3.55339	6.32E-04	0.002581	-0.69545	ATP5PO	c("ADIPOGENESIS", "OXIDATIVE_PHOSPHORYLATION")
1.450375	-0.01548	3.54936	6.41E-04	0.002601	-0.70768	SIRPA	APICAL_JUNCTION
-0.77256	-0.04713	-3.54651	6.47E-04	0.002621	-0.71634	SYMPK	APICAL_JUNCTION
1.883366	-0.31362	3.546338	6.47E-04	0.002621	-0.71686	CXCL12	c("EPITHELIAL_MESENCHYMAL_TRANSITION", "ESTROGEN_RESPONSE_EARLY", "ESTROGEN_RESPONSE_LATE")
-0.69908	-0.044	-3.5453	6.49E-04	0.002628	-0.72002	RAD50	E2F_TARGETS
-1.17089	-0.108	-3.54465	6.51E-04	0.002632	-0.72197	SUCLA2	c("FATTY_ACID_METABOLISM", "OXIDATIVE_PHOSPHORYLATION")
-0.65321	-0.092	-3.54416	6.52E-04	0.002632	-0.72348	RPS12	P53_PATHWAY
-0.85534	-0.0252	-3.54208	6.56E-04	0.002645	-0.72977	DST	c("EPITHELIAL_MESENCHYMAL_TRANSITION", "MITOTIC_SPINDLE", "PROTEIN_SECRETION")

1.56 1523	- 0.11 3	3.54 1334	6.58 E-04	0.00 2646	- 0.73 204	ACAD S	c("ADIPOGENESIS", "FATTY_ACID_METABOLISM")
0.77 196	- 0.01 275	3.54 0067	6.61 E-04	0.00 2652	- 0.73 588	UAP1	ANDROGEN_RESPONSE
1.14 4683	- 0.17 55	3.53 7361	6.66 E-04	0.00 2673	- 0.74 408	CTSC	c("COMPLEMENT", "MTORC1_SIGNALING", "PROTEIN_SECRETION")
- 1.23 064	- 0.12 57	- 3.53 531	6.71 E-04	0.00 2687	- 0.75 031	GLRX5	HEME_METABOLISM
- 1.36 334	- 0.37 047	- 3.53 302	6.76 E-04	0.00 2703	- 0.75 724	ECSIT	PI3K_AKT_MTOR_SIGNALING
- 1.38 177	- 0.11 25	- 3.53 244	6.77 E-04	0.00 2707	- 0.75 898	TIMM5 0	OXIDATIVE_PHOSPHORYLATION
1.13 1764	- 0.08 913	3.53 0149	6.82 E-04	0.00 2721	- 0.76 591	SWAP 70	IL2_STAT5_SIGNALING
- 1.28 32	- 0.14 9	- 3.52 456	6.95 E-04	0.00 2761	- 0.78 283	EHHA DH	c("FATTY_ACID_METABOLISM", "PEROXISOME")
1.44 2192	- 0.35 267	3.52 1247	7.03 E-04	0.00 2787	- 0.79 282	CD82	c("INFLAMMATORY_RESPONSE", "P53_PATHWAY")
- 0.94 653	- 0.12 413	- 3.52 092	7.03 E-04	0.00 2788	- 0.79 381	CDK2	c("APOPTOSIS", "MYC_TARGETS_V1", "PI3K_AKT_MTOR_SIGNALING", "UV_RESPONSE_UP")
2.67 5855	- 0.38 638	3.52 0163	7.05 E-04	0.00 2793	- 0.79 609	FBN1	c("APICAL_JUNCTION", "COAGULATION", "EPITHELIAL_MESENCHYMAL_TR ANSITION")
- 0.97 064	- 0.16 138	- 3.51 418	7.19 E-04	0.00 2839	- 0.81 415	STAU1	MITOTIC_SPINDLE
- 1.18 161	- 0.03 778	- 3.51 008	7.29 E-04	0.00 2869	- 0.82 651	SLC25 A17	PEROXISOME
- 0.70 494	- 0.02 038	- 3.50 491	7.41 E-04	0.00 2909	- 0.84 206	RFC4	c("DNA_REPAIR", "MYC_TARGETS_V1", "SPERMATOGENESIS", "UV_RESPONSE_UP")

1.858969	-0.38545	3.499926	7.53E-04	0.002949	-0.85706	FPR1	c("INFLAMMATORY_RESPONSE", "INTERFERON_GAMMA_RESPONSE")
-0.59461	-0.05025	-3.49966	7.54E-04	0.002949	-0.85786	DDB1	DNA_REPAIR
-1.28719	-0.57732	-3.49703	7.60E-04	0.002972	-0.86577	MRPL35	OXIDATIVE_PHOSPHORYLATION
-0.87111	-0.06113	-3.49525	7.65E-04	0.002987	-0.87109	NDUFB5	OXIDATIVE_PHOSPHORYLATION
-0.84823	-0.06888	-3.49227	7.72E-04	0.003012	-0.88005	COX6B1	OXIDATIVE_PHOSPHORYLATION
1.383544	-0.44066	3.491811	7.73E-04	0.003014	-0.88143	GUCY1B1	IL2_STAT5_SIGNALING
-1.28047	-0.66874	-3.49132	7.75E-04	0.003017	-0.88289	GAB2	ESTROGEN_RESPONSE_EARLY
2.764546	-0.543	3.490618	7.76E-04	0.003022	-0.88501	COL3A1	c("ANGIOGENESIS", "EPITHELIAL_MESENCHYMAL_TRANSITION", "MYOGENESIS", "UV_RESPONSE_DN")
1.148117	-0.14338	3.486458	7.87E-04	0.003058	-0.89749	TGFB1	c("ALLOGRAFT_REJECTION", "EPITHELIAL_MESENCHYMAL_TRANSITION", "G2M_CHECKPOINT", "IL6_JAK_STAT3_SIGNALING", "MYOGENESIS", "P53_PATHWAY", "TGF_BETA_SIGNALING")
-1.03008	-0.08288	-3.48414	7.93E-04	0.003075	-0.90443	UQCRQ	c("ADIPOGENESIS", "OXIDATIVE_PHOSPHORYLATION")
-0.83308	-0.11963	-3.4827	7.97E-04	0.003085	-0.90875	YWHAQ	MYC_TARGETS_V1
-0.68148	-0.08013	-3.47932	8.06E-04	0.003114	-0.91886	AIMP2	c("MYC_TARGETS_V1", "MYC_TARGETS_V2")
-0.90116	-0.13838	-3.47742	8.11E-04	0.003128	-0.92456	PPM1G	MYC_TARGETS_V1
-1.01917	-0.11213	-3.47708	8.11E-04	0.003128	-0.92558	FDPS	c("CHOLESTEROL_HOMEOSTASIS", "MYOGENESIS", "PEROXISOME")

-0.78824	-0.0685	-3.47538	8.16E-04	0.003143	-0.93065	PEX11B	PEROXISOME
0.802934	-0.05125	3.475213	8.16E-04	0.003143	-0.93115	ARHGDIA	c("MITOTIC_SPINDLE", "PI3K_AKT_MTOR_SIGNALING")
-1.59519	-0.54362	-3.47039	8.29E-04	0.003185	-0.94556	SNAP91	c("KRAS_SIGNALING_UP", "SPERMATOGENESIS")
-0.91893	0.024902	-3.46967	8.31E-04	0.00319	-0.94773	PDP1	c("COMPLEMENT", "OXIDATIVE_PHOSPHORYLATION")
-0.87963	-0.06463	-3.46917	8.33E-04	0.003193	-0.94921	UQCRFS1	OXIDATIVE_PHOSPHORYLATION
1.709707	-0.19063	3.454097	8.74E-04	0.003334	-0.99416	ACTA2	EPITHELIAL_MESENCHYMAL_TRANSITION
1.266428	-0.3019	3.453425	8.76E-04	0.003336	-0.99616	CAP2	c("EPITHELIAL_MESENCHYMAL_TRANSITION", "UV_RESPONSE_DN")
-1.72531	-0.36763	-3.45186	8.81E-04	0.003346	-1.00082	PYCR1	XENOBIOTIC_METABOLISM
3.297636	-1.17163	3.435568	9.28E-04	0.003502	-1.04921	DES	MYOGENESIS
-1.04884	-0.12255	-3.43496	9.30E-04	0.003506	-1.051	LLGL2	ESTROGEN_RESPONSE_LATE
0.809184	-0.06325	3.42406	9.63E-04	0.003614	-1.08329	ACP2	XENOBIOTIC_METABOLISM
1.048727	-0.39831	3.416058	9.88E-04	0.003692	-1.10694	GOSR2	c("PROTEIN_SECRETION", "UNFOLDED_PROTEIN_RESPONSE")
-0.57214	-0.088	-3.40148	0.001036	0.00385	-1.1499	RPS19	ALLOGRAFT_REJECTION
-0.65323	-0.102	-3.39718	0.00105	0.003888	-1.16256	RPL22	MYC_TARGETS_V1
-0.98813	-0.04513	-3.39392	0.001061	0.003914	-1.17216	POU2F1	IL2_STAT5_SIGNALING

-0.78021	-0.0505	-3.39373	0.001062	0.003914	-1.17271	CTCF	c("E2F_TARGETS", "G2M_CHECKPOINT")
1.535093	-0.25927	3.392804	0.001065	0.003921	-1.17542	RETSAT	c("ADIPOGENESIS", "APOPTOSIS", "BILE_ACID_METABOLISM", "FATTY_ACID_METABOLISM", "OXIDATIVE_PHOSPHORYLATION", "P53_PATHWAY", "PEROXISOME", "XENOBIOTIC_METABOLISM")
0.700522	0.04075	3.388833	0.001078	0.003965	-1.18708	IGF2R	c("APOPTOSIS", "IL2_STAT5_SIGNALING", "PROTEIN_SECRETION")
-1.33259	-0.62618	-3.38843	0.00108	0.003965	-1.18826	TTC39B	IL2_STAT5_SIGNALING
1.221609	-0.15225	3.375778	0.001124	0.0041	-1.22534	GCA	COMPLEMENT
1.021607	-0.11188	3.374826	0.001128	0.00411	-1.22813	BLVRA	c("FATTY_ACID_METABOLISM", "HEME_METABOLISM")
2.215172	-0.87525	3.372618	0.001136	0.004126	-1.23459	THBS1	c("COAGULATION", "EPITHELIAL_MESENCHYMAL_TRANSITION", "TGF_BETA_SIGNALING")
-0.58609	-0.08825	-3.36692	0.001157	0.004181	-1.25125	RPS3A	ALLOGRAFT_REJECTION
-0.61903	-0.1055	-3.36392	0.001168	0.004212	-1.25999	RPS3	MYC_TARGETS_V1
-1.10135	-0.07688	-3.36288	0.001171	0.004223	-1.26304	MGST3	c("ADIPOGENESIS", "HEME_METABOLISM", "OXIDATIVE_PHOSPHORYLATION")
1.251343	-0.04538	3.362236	0.001174	0.004229	-1.26491	CD14	c("APOPTOSIS", "IL6_JAK_STAT3_SIGNALING", "INFLAMMATORY_RESPONSE")
-1.21033	-0.54361	-3.3614	0.001177	0.004232	-1.26737	DMAC2L	UV_RESPONSE_DN
-1.50976	-0.81126	-3.36008	0.001182	0.004247	-1.27122	KATNA1	c("G2M_CHECKPOINT", "MITOTIC_SPINDLE")

-0.72699	-0.09075	-3.35507	0.001201	0.004303	-1.28579	TCEA1	c("HEME_METABOLISM", "MTORC1_SIGNALING")
-0.6585	-0.05513	-3.3529	0.001209	0.004327	-1.29212	SMC3	c("E2F_TARGETS", "MITOTIC_SPINDLE")
-1.26899	-0.6025	-3.34523	0.001239	0.004419	-1.31445	CHPF2	GLYCOLYSIS
0.651395	-0.0435	3.340767	0.001257	0.004476	-1.3274	GNAI3	c("ANDROGEN_RESPONSE", "COMPLEMENT", "INFLAMMATORY_RESPONSE")
-1.1088	-0.15738	-3.33355	0.001286	0.004561	-1.34833	FHL2	c("ESTROGEN_RESPONSE_EARLY", "UV_RESPONSE_DN")
0.783141	-0.08775	3.325439	0.001319	0.004664	-1.37183	ACTR2	c("MTORC1_SIGNALING", "PI3K_AKT_MTOR_SIGNALING")
-0.90888	-0.03713	-3.32445	0.001323	0.004676	-1.37469	HSD17B10	c("FATTY_ACID_METABOLISM", "OXIDATIVE_PHOSPHORYLATION")
-0.87528	-0.09975	-3.32263	0.001331	0.00469	-1.37995	NCL	G2M_CHECKPOINT
-1.46669	-0.69464	-3.32143	0.001336	0.004702	-1.38342	SMC6	E2F_TARGETS
-1.22159	-0.2889	-3.32118	0.001337	0.004702	-1.38416	DHCR7	c("ADIPOGENESIS", "CHOLESTEROL_HOMEOSTASIS", "ESTROGEN_RESPONSE_EARLY", "ESTROGEN_RESPONSE_LATE", "MTORC1_SIGNALING")
2.374434	-1.50861	3.320233	0.001341	0.004713	-1.38689	CD209	APICAL_JUNCTION
-0.97864	-0.14388	-3.32003	0.001342	0.004713	-1.38747	FEN1	c("DNA_REPAIR", "UV_RESPONSE_UP")
0.845782	0.013625	3.314943	0.001364	0.004771	-1.40217	RRAS	APICAL_JUNCTION
-0.90263	-0.0405	-3.30562	0.001404	0.004899	-1.42904	TCOF1	MYC_TARGETS_V2
-1.25488	-0.18225	-3.30487	0.001408	0.004905	-1.43122	FDXR	c("APOPTOSIS", "BILE_ACID_METABOLISM",

							"MTORC1_SIGNALING", "P53_PATHWAY")
-0.77798	-0.04788	-3.3013	0.001423	0.004945	-1.4415	NDUFA4	OXIDATIVE_PHOSPHORYLATION
2.055608	-0.30166	3.29891	0.001434	0.004978	-1.44836	CD48	c("IL2_STAT5_SIGNALING", "INFLAMMATORY_RESPONSE")
1.508053	-0.21975	3.297357	0.001441	0.004992	-1.45283	TUBB2B	ESTROGEN_RESPONSE_EARLY
1.620505	-0.14325	3.297242	0.001442	0.004992	-1.45316	ALDH1A1	c("BILE_ACID_METABOLISM", "FATTY_ACID_METABOLISM", "PEROXISOME")
-0.81905	-0.10713	-3.29241	0.001464	0.00505	-1.46703	EEF1E1	MTORC1_SIGNALING
0.773064	-0.0905	3.292305	0.001464	0.00505	-1.46735	ARPC3	PI3K_AKT_MTOR_SIGNALING
-0.65923	0.023181	-3.28874	0.001481	0.005103	-1.47757	AKAP1	ESTROGEN_RESPONSE_EARLY
1.563736	-0.092	3.273109	0.001555	0.005322	-1.52235	ACSL1	c("BILE_ACID_METABOLISM", "FATTY_ACID_METABOLISM", "MYOGENESIS", "PEROXISOME")
1.475307	-0.12388	3.266845	0.001586	0.005417	-1.54024	PTPRJ	KRAS_SIGNALING_DN
0.883438	-0.08538	3.265761	0.001591	0.00543	-1.54333	GRK2	PI3K_AKT_MTOR_SIGNALING
1.671555	-0.2632	3.265651	0.001592	0.00543	-1.54365	APOC2	COAGULATION
1.234288	-0.04888	3.262954	0.001605	0.005472	-1.55134	APOD	c("KRAS_SIGNALING_UP", "MYOGENESIS")
-2.29402	-0.3535	-3.26145	0.001613	0.005494	-1.55562	TOP2A	c("APOPTOSIS", "E2F_TARGETS", "ESTROGEN_RESPONSE_LATE", "G2M_CHECKPOINT", "MITOTIC_SPINDLE", "PEROXISOME")
-1.17022	-0.23238	-3.25501	0.001645	0.005592	-1.57399	TRIM28	MYC_TARGETS_V1

1.215526	-0.14538	3.254266	0.001649	0.005599	-1.5761	GBP2	c("ALLOGRAFT_REJECTION", "INTERFERON_ALPHA_RESPONSE")
-1.27186	-0.76277	-3.25396	0.001651	0.005599	-1.57696	RHOB	c("APOPTOSIS", "EPITHELIAL_MESENCHYMAL_TRANSITION", "IL2_STAT5_SIGNALING", "TNFA_SIGNALING_VIA_NFKB", "UV_RESPONSE_UP")
-0.90432	-0.066	-3.25246	0.001658	0.005622	-1.58124	DBT	ADIPOGENESIS
-0.91214	-0.08263	-3.24875	0.001678	0.005666	-1.5918	COQ9	ADIPOGENESIS
-1.08496	-0.09563	-3.24848	0.001679	0.005667	-1.59255	PRDX3	c("ADIPOGENESIS", "MYC_TARGETS_V1", "OXIDATIVE_PHOSPHORYLATION")
-0.88521	-0.18037	-3.24754	0.001684	0.005676	-1.59522	TMBIM6	c("UV_RESPONSE_UP", "XENOBIOTIC_METABOLISM")
-0.9993	-0.69827	-3.2426	0.00171	0.005744	-1.60925	EI24	P53_PATHWAY
-0.64028	-0.11788	-3.24181	0.001714	0.005749	-1.6115	MON2	PROTEIN_SECRETION
0.610339	-0.02813	3.237924	0.001735	0.005804	-1.62252	HOOK3	MITOTIC_SPINDLE
1.183088	-0.08588	3.237729	0.001736	0.005804	-1.62307	GGH	UV_RESPONSE_UP
1.415819	-0.1145	3.235507	0.001748	0.005841	-1.62937	MYL9	c("APICAL_JUNCTION", "EPITHELIAL_MESENCHYMAL_TRANSITION")
-0.90625	-0.13775	-3.23485	0.001752	0.005849	-1.63122	DTYMK	G2M_CHECKPOINT
1.768298	-0.23913	3.229937	0.001779	0.005924	-1.64514	MGLL	c("ADIPOGENESIS", "FATTY_ACID_METABOLISM", "UV_RESPONSE_DN")
-0.98988	-0.15463	-3.22826	0.001788	0.005944	-1.64988	FKBP4	c("ESTROGEN_RESPONSE_EARLY", "ESTROGEN_RESPONSE_LATE",

							"GLYCOLYSIS", "UV_RESPONSE_UP")
1.09 0699	- 0.39 899	3.22 6828	0.00 1796	0.00 5958	- 1.65 394	SRP19	ANDROGEN_RESPONSE
1.03 724	- 0.34 799	3.22 6688	0.00 1797	0.00 5958	- 1.65 433	NFYB	UNFOLDED_PROTEIN_RESPONS E
1.53 7332	- 0.14 363	3.22 5774	0.00 1802	0.00 5971	- 1.65 692	BPGM	c("HEME_METABOLISM", "INTERFERON_GAMMA_RESPON SE", "KRAS_SIGNALING_UP")
1.07 0477	- 0.55 589	3.22 1298	0.00 1827	0.00 6036	- 1.66 956	RBX1	c("DNA_REPAIR", "NOTCH_SIGNALING")
2.05 0187	- 0.28 463	3.22 0463	0.00 1832	0.00 6047	- 1.67 192	FGA	COAGULATION
- 0.82 506	- 0.11 238	- 3.22 033	0.00 1832	0.00 6047	- 1.67 231	ARL3	c("ESTROGEN_RESPONSE_EARL Y", "ESTROGEN_RESPONSE_LATE")
- 1.00 745	- 0.79 17	- 3.21 418	0.00 1868	0.00 6136	- 1.68 964	EXT2	GLYCOLYSIS
1.36 6974	- 0.10 045	3.20 6867	0.00 191	0.00 6261	- 1.71 025	COL15 A1	c("ADIPOGENESIS", "MYOGENESIS")
- 1.27 25	- 0.55 974	- 3.20 198	0.00 1939	0.00 6341	- 1.72 4	UCK2	c("G2M_CHECKPOINT", "IL2_STAT5_SIGNALING")
- 1.42 818	- 0.49 423	- 3.19 86	0.00 196	0.00 6387	- 1.73 348	EFEM P2	EPITHELIAL_MESENCHYMAL_TR ANSITION
- 0.94 87	- 0.08 456	- 3.19 611	0.00 1975	0.00 6425	- 1.74 048	RABG AP1L	c("IL2_STAT5_SIGNALING", "KRAS_SIGNALING_UP")
- 1.12 166	- 0.71 459	- 3.19 227	0.00 1998	0.00 6497	- 1.75 126	RALBP 1	MITOTIC_SPINDLE
- 0.98 066	- 0.16 288	- 3.18 732	0.00 2029	0.00 6589	- 1.76 514	FH	c("FATTY_ACID_METABOLISM", "OXIDATIVE_PHOSPHORYLATION ")
- 1.76 215	- 0.32 771	- 3.18 706	0.00 203	0.00 659	- 1.76 585	HDAC 2	c("MYC_TARGETS_V1", "WNT_BETA_CATENIN_SIGNALIN G")
2.01 7555	- 0.23 438	3.18 4926	0.00 2044	0.00 6621	- 1.77 183	FGG	COAGULATION

0.93058	-0.26659	3.184027	0.00205	0.006632	-1.77435	NDUFC2	OXIDATIVE_PHOSPHORYLATION
1.240254	-0.2195	3.183453	0.002053	0.006639	-1.77595	WARS1	c("ALLOGRAFT_REJECTION", "INTERFERON_ALPHA_RESPONSE", "INTERFERON_GAMMA_RESPONSE", "MTORC1_SIGNALING")
1.099905	-0.55272	3.183074	0.002056	0.006639	-1.77701	SIGMAR1	UV_RESPONSE_UP
-0.94067	-0.17538	-3.18162	0.002065	0.006661	-1.78109	SMARCC1	c("G2M_CHECKPOINT", "MYC_TARGETS_V1", "PEROXISOME")
-0.65646	-0.07763	-3.18074	0.00207	0.006675	-1.78355	HNRNPA3	MYC_TARGETS_V1
-1.03425	-0.092	-3.17966	0.002077	0.006693	-1.78657	SLC25A1	c("ADIPOGENESIS", "HYPOXIA")
-1.15413	-0.65486	-3.16806	0.002152	0.006906	-1.81895	UBE2C	G2M_CHECKPOINT
-0.84172	-0.112	-3.16666	0.002162	0.006928	-1.82286	SDHA	c("FATTY_ACID_METABOLISM", "OXIDATIVE_PHOSPHORYLATION")
2.147251	-0.931	3.166622	0.002162	0.006928	-1.82296	FTL	REACTIVE_OXYGEN_SPECIES_PATHWAY
-2.39846	-0.6219	-3.16345	0.002183	0.006975	-1.8318	BTC	KRAS_SIGNALING_UP
-0.62261	-0.05925	-3.16265	0.002188	0.006984	-1.83403	ADAR	c("INTERFERON_ALPHA_RESPONSE", "INTERFERON_GAMMA_RESPONSE")
-0.9008	-0.18225	-3.16111	0.002199	0.007012	-1.83829	NSDHL	c("CHOLESTEROL_HOMEOSTASIS", "FATTY_ACID_METABOLISM", "GLYCOLYSIS")
-1.15291	-0.43974	-3.15765	0.002222	0.007074	-1.84792	EXOSC2	UNFOLDED_PROTEIN_RESPONSE
-0.55938	-0.10725	-3.1549	0.002241	0.007118	-1.85558	RPL6	MYC_TARGETS_V1

-0.65417	-0.10282	-3.15484	0.002241	0.007118	-1.85575	EXOSC9	UNFOLDED_PROTEIN_RESPONSE
1.029374	-0.07688	3.148475	0.002285	0.007232	-1.87341	MYLK	c("ADIPOGENESIS", "EPITHELIAL_MESENCHYMAL_TRANSITION", "MYOGENESIS")
1.105624	-0.12776	3.146521	0.002299	0.007267	-1.87884	ARFIP2	MITOTIC_SPINDLE
-1.12399	-0.58673	-3.14314	0.002323	0.007325	-1.88821	PRKCA	c("HYPOXIA", "NOTCH_SIGNALING", "UV_RESPONSE_DN")
0.556125	-0.0535	3.141135	0.002337	0.007365	-1.89376	ACLY	c("ADIPOGENESIS", "MTORC1_SIGNALING")
-0.72514	-0.067	-3.14054	0.002341	0.007371	-1.8954	ARFIP1	PROTEIN_SECRETION
-1.06419	-0.19013	-3.13712	0.002366	0.007434	-1.90487	FUT8	GLYCOLYSIS
0.774267	-0.06025	3.133618	0.002391	0.0075	-1.91456	SLC3A2	P53_PATHWAY
-0.47195	-0.05038	-3.13223	0.002401	0.007527	-1.9184	ARCN1	PROTEIN_SECRETION
-0.72803	-0.0735	-3.12184	0.002478	0.007733	-1.94706	PAIP1	UNFOLDED_PROTEIN_RESPONSE
-0.75187	-0.0325	-3.1209	0.002485	0.007745	-1.94965	NDUFA5	c("ADIPOGENESIS", "OXIDATIVE_PHOSPHORYLATION")
-1.22449	-0.69576	-3.12043	0.002489	0.007747	-1.95096	FBN2	EPITHELIAL_MESENCHYMAL_TRANSITION
-0.53923	-0.032	-3.11651	0.002518	0.007831	-1.96175	ETF1	c("APOPTOSIS", "MTORC1_SIGNALING", "MYC_TARGETS_V1")
-1.24933	-0.26786	-3.11225	0.002551	0.007928	-1.97345	SQLE	c("CHOLESTEROL_HOMEOSTASIS", "G2M_CHECKPOINT", "MTORC1_SIGNALING")
-0.54756	-0.10075	-3.10976	0.00257	0.007965	-1.98032	RPS6	MYC_TARGETS_V1

0.8023	-0.02288	3.109468	0.002573	0.007967	-1.98111	SAR1B	XENOBIOTIC_METABOLISM
0.873039	-0.12263	3.108384	0.002581	0.007984	-1.98409	GSTO1	c("IL2_STAT5_SIGNALING", "XENOBIOTIC_METABOLISM")
-0.70197	0.05825	-3.10451	0.002611	0.008069	-1.99472	U2AF1	MYC_TARGETS_V1
-1.03444	-0.10125	-3.10254	0.002627	0.008098	-2.00013	ECHS1	c("ADIPOGENESIS", "FATTY_ACID_METABOLISM", "OXIDATIVE_PHOSPHORYLATION")
0.970338	-0.09425	3.099974	0.002648	0.008152	-2.00716	EPB41L2	c("APICAL_JUNCTION", "MITOTIC_SPINDLE")
1.760445	-0.19779	3.098756	0.002657	0.008174	-2.0105	SORBS1	c("ADIPOGENESIS", "MYOGENESIS")
-0.77422	-0.09475	-3.09393	0.002696	0.008272	-2.02373	PPP1R8	E2F_TARGETS
1.13127	-0.13991	3.089661	0.002731	0.008362	-2.03539	CASP7	c("APOPTOSIS", "COMPLEMENT", "INTERFERON_GAMMA_RESPONSE", "OXIDATIVE_PHOSPHORYLATION")
-1.32163	-0.2775	-3.08964	0.002731	0.008362	-2.03545	AIFM1	c("ADIPOGENESIS", "OXIDATIVE_PHOSPHORYLATION")
-1.45429	-0.69479	-3.08862	0.00274	0.008378	-2.03824	RBPMS	UV_RESPONSE_DN
0.559941	-0.085	3.086745	0.002755	0.00842	-2.04336	PSMA1	MYC_TARGETS_V1
-1.12099	-0.50866	-3.08228	0.002793	0.008529	-2.05555	POLR2J	DNA_REPAIR
0.942693	-0.07413	3.081918	0.002796	0.008534	-2.05654	CTSZ	IL2_STAT5_SIGNALING
0.665142	0.009625	3.080573	0.002807	0.008559	-2.06021	PSMA2	c("INTERFERON_GAMMA_RESPONSE", "MYC_TARGETS_V1")

0.734558	-0.069	3.075179	0.002853	0.008669	-2.07491	GRB2	c("COMPLEMENT", "IL6_JAK_STAT3_SIGNALING", "PI3K_AKT_MTOR_SIGNALING")
-0.54317	-0.08688	-3.07239	0.002877	0.008737	-2.0825	TMED10	PROTEIN_SECRETION
-1.34706	-0.25838	-3.06729	0.002921	0.008844	-2.09638	DCTPP1	c("E2F_TARGETS", "MYC_TARGETS_V2")
2.873448	-0.82613	3.058524	0.002999	0.009046	-2.12017	COL1A2	c("EPITHELIAL_MESENCHYMAL_TRANSITION", "UV_RESPONSE_DN")
1.129214	-0.4184	3.058082	0.003003	0.009053	-2.12137	CYFIP2	c("P53_PATHWAY", "XENOBIOTIC_METABOLISM")
-0.96282	-0.1815	-3.05734	0.00301	0.009063	-2.12338	CSE1L	E2F_TARGETS
-0.86553	-0.20775	-3.05408	0.003039	0.009136	-2.13223	CALU	EPITHELIAL_MESENCHYMAL_TRANSITION
0.509884	0.05675	3.04843	0.003091	0.00926	-2.14751	MYD88	c("IL6_JAK_STAT3_SIGNALING", "INTERFERON_GAMMA_RESPONSE", "PI3K_AKT_MTOR_SIGNALING")
0.71366	-0.06605	3.042353	0.003147	0.009382	-2.16393	PGM2	c("GLYCOLYSIS", "HYPOXIA")
-1.00225	-0.08613	-3.03578	0.00321	0.009549	-2.18166	NUDT19	PEROXISOME
-0.71562	-0.03663	-3.03568	0.003211	0.009549	-2.18193	NDUFV1	OXIDATIVE_PHOSPHORYLATION
-0.96359	-0.165	-3.03386	0.003228	0.009585	-2.18684	FIS1	PEROXISOME
1.57702	-0.18288	3.02941	0.003271	0.009697	-2.19883	BASP1	EPITHELIAL_MESENCHYMAL_TRANSITION
-0.68655	-0.07425	-3.02908	0.003274	0.009701	-2.19972	ATP5F1D	OXIDATIVE_PHOSPHORYLATION
0.740178	-0.04238	3.025509	0.003309	0.009794	-2.20932	CBL	c("IL6_JAK_STAT3_SIGNALING", "KRAS_SIGNALING_UP")

-1.16497	-0.27915	-3.02251	0.003339	0.009861	-2.21737	CDCP1	IL2_STAT5_SIGNALING
-0.71509	-0.06713	-3.01664	0.003398	0.01	-2.23313	TIMM13	OXIDATIVE_PHOSPHORYLATION
-1.69467	-0.56879	-3.01562	0.003408	0.010019	-2.23587	KLK10	c("ESTROGEN_RESPONSE_EARLY", "ESTROGEN_RESPONSE_LATE")
0.6024	-0.057	3.014261	0.003422	0.010048	-2.23952	MYH9	c("APICAL_JUNCTION", "HEDGEHOG_SIGNALING", "HYPOXIA", "MITOTIC_SPINDLE", "MYOGENESIS")
1.011192	-0.11725	3.011817	0.003447	0.010116	-2.24607	ICAM1	c("ALLOGRAFT_REJECTION", "APICAL_JUNCTION", "INFLAMMATORY_RESPONSE", "INTERFERON_GAMMA_RESPONSE", "TNFA_SIGNALING_VIA_NFKB", "UV_RESPONSE_UP")
-1.19486	-0.23538	-3.01068	0.003458	0.010144	-2.24911	CBX5	E2F_TARGETS
-1.45612	-0.28543	-3.00802	0.003486	0.010197	-2.25625	CLDN3	GLYCOLYSIS
0.636345	-0.107	3.007741	0.003488	0.0102	-2.25698	GMPPB	GLYCOLYSIS
-0.82486	-0.03	-3.00648	0.003501	0.010222	-2.26034	LCLAT1	IL2_STAT5_SIGNALING
-0.73532	-0.05625	-3.00334	0.003534	0.010299	-2.26874	DLD	c("ADIPOGENESIS", "FATTY_ACID_METABOLISM", "GLYCOLYSIS", "OXIDATIVE_PHOSPHORYLATION")
-0.5779	-0.0785	-3.00285	0.003539	0.010303	-2.27005	HNRNPA2B1	MYC_TARGETS_V1
-1.3266	-0.72846	-3.00143	0.003554	0.010324	-2.27386	PYGM	c("HYPOXIA", "MYOGENESIS")
-0.86728	-0.09775	-2.99684	0.003603	0.010436	-2.2861	UQCRB	OXIDATIVE_PHOSPHORYLATION

0.55825	-0.03	2.996296	0.003608	0.010448	-2.28756	ATP6V1E1	OXIDATIVE_PHOSPHORYLATION
-0.73141	-0.14263	-2.98277	0.003755	0.010792	-2.32357	COG2	c("GLYCOLYSIS", "PROTEIN_SECRETION")
-0.55192	-0.1165	-2.98275	0.003756	0.010792	-2.32363	RPS10	MYC_TARGETS_V1
-1.12114	-0.16216	-2.97969	0.003789	0.01086	-2.33176	TUBGCP6	MITOTIC_SPINDLE
2.302855	-0.52938	2.977471	0.003814	0.010919	-2.33766	COL5A2	c("ANGIOGENESIS", "EPITHELIAL_MESENCHYMAL_TRANSITION", "UV_RESPONSE_DN")
-0.50177	-0.06588	-2.97656	0.003825	0.010937	-2.34008	SHC1	c("APICAL_JUNCTION", "UNFOLDED_PROTEIN_RESPONSE")
-0.93583	-0.15813	-2.97482	0.003844	0.010976	-2.3447	MSH2	c("E2F_TARGETS", "PEROXISOME")
0.751711	-0.07263	2.974538	0.003847	0.010979	-2.34544	LTA4H	c("COAGULATION", "COMPLEMENT", "MTORC1_SIGNALING")
-0.63066	-0.08325	-2.97314	0.003863	0.011012	-2.34915	DLST	c("FATTY_ACID_METABOLISM", "OXIDATIVE_PHOSPHORYLATION")
-0.49717	-0.07875	-2.96321	0.003978	0.01132	-2.37546	EIF4A1	c("MYC_TARGETS_V1", "UNFOLDED_PROTEIN_RESPONSE")
-1.10229	-0.18463	-2.96234	0.003988	0.011343	-2.37775	IDH2	c("BILE_ACID_METABOLISM", "ESTROGEN_RESPONSE_LATE", "OXIDATIVE_PHOSPHORYLATION", "PEROXISOME")
-0.83494	-0.15663	-2.95951	0.004021	0.011413	-2.38523	AHCY	c("IL2_STAT5_SIGNALING", "XENOBIOTIC_METABOLISM")
-0.93766	-0.19063	-2.95889	0.004028	0.011427	-2.38685	GRPEL1	c("ADIPOGENESIS", "OXIDATIVE_PHOSPHORYLATION", "UV_RESPONSE_UP")
1.104594	-0.10875	2.958502	0.004033	0.011434	-2.38788	PNP	c("DNA_REPAIR", "IL2_STAT5_SIGNALING", "INTERFERON_GAMMA_RESPONSE", "MTORC1_SIGNALING")
0.899298	-0.107	2.95794	0.004039	0.011447	-2.38937	FUCA1	c("EPITHELIAL_MESENCHYMAL_TRANSITION",

							"KRAS_SIGNALING_UP", "P53_PATHWAY")
-0.86809	-0.04732	-2.95591	0.004063	0.011503	-2.39474	MRPL34	OXIDATIVE_PHOSPHORYLATION
-0.93935	-0.10613	-2.95491	0.004075	0.011531	-2.39737	HMGCL	c("FATTY_ACID_METABOLISM", "PEROXISOME")
-0.75374	-0.14988	-2.95387	0.004088	0.011553	-2.40011	HSP90AB1	MYC_TARGETS_V1
0.647657	-0.02588	2.953534	0.004092	0.011559	-2.40099	OPTN	c("BILE_ACID_METABOLISM", "HEME_METABOLISM")
1.028818	-0.09563	2.947996	0.004159	0.011741	-2.41559	SAMHD1	INTERFERON_GAMMA_RESPONSE
-0.94863	-0.37398	-2.9447	0.004199	0.011836	-2.42428	TMEM97	c("CHOLESTEROL_HOMEOSTASIS", "MTORC1_SIGNALING", "MYC_TARGETS_V2", "XENOBIOTIC_METABOLISM")
0.914215	-0.28975	2.937924	0.004283	0.012047	-2.44208	STK38L	MITOTIC_SPINDLE
-1.3262	-0.35588	-2.93681	0.004297	0.01208	-2.445	SPINT2	XENOBIOTIC_METABOLISM
-0.92193	-0.06376	-2.93561	0.004312	0.012109	-2.44815	PEX26	BILE_ACID_METABOLISM
-0.58968	0.063875	-2.92871	0.004399	0.012323	-2.46624	SRSF3	MYC_TARGETS_V1
-1.23231	-0.04051	-2.92804	0.004408	0.012338	-2.46799	MYBBP1A	c("ESTROGEN_RESPONSE_EARLY", "MYC_TARGETS_V2")
-0.90392	-0.14375	-2.92773	0.004412	0.012338	-2.4688	CNBP	c("MYC_TARGETS_V1", "PEROXISOME")
-1.15947	-0.58119	-2.92464	0.004452	0.012411	-2.4769	NOTCH3	NOTCH_SIGNALING
-0.71391	-0.0375	-2.92383	0.004462	0.012424	-2.47902	NDUFA9	OXIDATIVE_PHOSPHORYLATION

0.863191	-0.10088	2.921707	0.00449	0.012491	-2.48457	GM2A	P53_PATHWAY
-0.906	-0.16713	-2.92119	0.004496	0.012503	-2.48593	NNT	c("HEME_METABOLISM", "OXIDATIVE_PHOSPHORYLATION")
-1.20188	-0.1225	-2.91769	0.004542	0.012607	-2.49508	LMNA	APOPTOSIS
0.967648	0.018919	2.917639	0.004543	0.012607	-2.4952	CD63	PROTEIN_SECRETION
0.491803	-0.039	2.910401	0.004639	0.012854	-2.51408	CAB39	PI3K_AKT_MTOR_SIGNALING
-0.60223	-0.00988	-2.90913	0.004656	0.012884	-2.51739	GRHPR	c("FATTY_ACID_METABOLISM", "HYPOXIA")
-0.65372	-0.066	-2.90642	0.004693	0.012956	-2.52446	ABCF2	MTORC1_SIGNALING
-1.04425	-0.21163	-2.90602	0.004699	0.012964	-2.5255	CRYZ	FATTY_ACID_METABOLISM
-0.90431	-0.28449	-2.90381	0.004729	0.013014	-2.53125	ANKZF1	c("GLYCOLYSIS", "HYPOXIA")
1.020273	-0.10438	2.900178	0.004779	0.013138	-2.54069	CTSH	c("COAGULATION", "COMPLEMENT")
2.057342	-0.47203	2.897172	0.00482	0.013239	-2.5485	BANK1	INTERFERON_GAMMA_RESPONSE
0.741229	-0.10375	2.894635	0.004856	0.013324	-2.55508	PTPN1	c("IL6_JAK_STAT3_SIGNALING", "INTERFERON_GAMMA_RESPONSE")
-0.67296	-0.02463	-2.88997	0.004922	0.013486	-2.56717	CDKAL1	KRAS_SIGNALING_DN
1.535621	-0.05213	2.889866	0.004923	0.013486	-2.56745	GPX3	c("ADIPOGENESIS", "APOPTOSIS", "MYOGENESIS", "REACTIVE_OXYGEN_SPECIES_PATHWAY", "UV_RESPONSE_UP")
-1.89854	-0.63575	-2.88682	0.004966	0.013591	-2.57533	SFN	c("ESTROGEN_RESPONSE_EARLY", "ESTROGEN_RESPONSE_LATE",

							"P53_PATHWAY", "PI3K_AKT_MTOR_SIGNALING")
-1.08807	-0.24425	-2.88181	0.005039	0.013774	-2.5883	CYCS	OXIDATIVE_PHOSPHORYLATION
1.803869	-0.18388	2.877598	0.0051	0.013921	-2.59918	COL6A1	IL2_STAT5_SIGNALING
-0.95191	-0.87842	-2.87341	0.005162	0.014082	-2.60998	SERPINE1	c("COAGULATION", "COMPLEMENT", "EPITHELIAL_MESENCHYMAL_TRANSITION", "HYPOXIA", "INFLAMMATORY_RESPONSE", "TGF_BETA_SIGNALING", "TNFA_SIGNALING_VIA_NFKB", "UV_RESPONSE_DN", "XENOBIOTIC_METABOLISM")
-0.78831	-0.11038	-2.87176	0.005186	0.014142	-2.61425	COX6C	c("ESTROGEN_RESPONSE_LATE", "OXIDATIVE_PHOSPHORYLATION")
-0.95042	-0.65377	-2.87087	0.0052	0.014167	-2.61654	SCD	c("ANDROGEN_RESPONSE", "CHOLESTEROL_HOMEOSTASIS", "MTORC1_SIGNALING", "MYOGENESIS")
-0.61299	-0.06263	-2.86846	0.005236	0.01424	-2.62275	DNAJA1	c("APOPTOSIS", "UV_RESPONSE_UP")
-1.00766	-0.21163	-2.86532	0.005283	0.014347	-2.63084	AK2	c("ADIPOGENESIS", "E2F_TARGETS")
-0.52336	0.004875	-2.85734	0.005405	0.014642	-2.65136	SMC1A	c("E2F_TARGETS", "G2M_CHECKPOINT", "MITOTIC_SPINDLE")
-1.11096	-0.64511	-2.85706	0.00541	0.014642	-2.65206	NRP1	c("ANGIOGENESIS", "HEDGEHOG_SIGNALING", "IL2_STAT5_SIGNALING", "KRAS_SIGNALING_UP", "UV_RESPONSE_DN")
2.05664	-0.3455	2.856816	0.005413	0.014642	-2.65269	LUM	c("ANGIOGENESIS", "APOPTOSIS", "EPITHELIAL_MESENCHYMAL_TRANSITION")
1.162662	-0.30216	2.84183	0.00565	0.015213	-2.69108	CELF2	UV_RESPONSE_DN

-0.52733	-0.043	-2.83784	0.005715	0.015371	-2.70127	CNOT2	UNFOLDED_PROTEIN_RESPONSE
-1.15641	-0.44788	-2.83594	0.005746	0.015447	-2.70612	DHPS	XENOBIOTIC_METABOLISM
-0.79775	-0.07485	-2.83065	0.005833	0.015633	-2.71961	ATP2C1	c("INFLAMMATORY_RESPONSE", "UV_RESPONSE_DN")
1.804236	-0.94163	2.829769	0.005848	0.015658	-2.72186	KRT1	c("ALLOGRAFT_REJECTION", "KRAS_SIGNALING_DN")
-1.12467	-0.1175	-2.82603	0.00591	0.015809	-2.73137	SCP2	c("ADIPOGENESIS", "BILE_ACID_METABOLISM", "PEROXISOME")
2.19447	-0.36953	2.824225	0.005941	0.015867	-2.73597	AOC3	FATTY_ACID_METABOLISM
-1.33024	-0.30675	-2.82246	0.005971	0.015938	-2.74045	PSAT1	c("MTORC1_SIGNALING", "UNFOLDED_PROTEIN_RESPONSE")
0.760208	-0.27799	2.82205	0.005977	0.015943	-2.7415	TXNL4A	MYC_TARGETS_V1
1.017302	-0.09538	2.819667	0.006018	0.016025	-2.74756	TPM1	EPITHELIAL_MESENCHYMAL_TRANSITION
-0.62558	-0.12875	-2.81573	0.006086	0.016173	-2.75756	G3BP2	ADIPOGENESIS
-0.62784	-0.07413	-2.81296	0.006134	0.016244	-2.76457	DR1	G2M_CHECKPOINT
-1.11385	-0.20538	-2.81047	0.006177	0.016318	-2.77089	SHMT2	c("MTORC1_SIGNALING", "XENOBIOTIC_METABOLISM")
-0.73001	-0.03325	-2.80631	0.00625	0.016487	-2.78142	NDUFB3	OXIDATIVE_PHOSPHORYLATION
-0.95495	-0.18388	-2.8053	0.006268	0.016522	-2.78398	LIG1	c("DNA_REPAIR", "E2F_TARGETS")
-0.69885	-0.01388	-2.8031	0.006307	0.016613	-2.78952	RBBP7	E2F_TARGETS

1.835631	-0.25338	2.795328	0.006447	0.016915	-2.80916	COL6A3	c("EPITHELIAL_MESENCHYMAL_TRANSITION", "MYOGENESIS")
-0.70284	-0.02838	-2.78852	0.006572	0.017234	-2.8263	DKC1	c("G2M_CHECKPOINT", "UNFOLDED_PROTEIN_RESPONSE")
-1.43901	-0.5052	-2.78465	0.006644	0.017389	-2.83605	GPRC5B	KRAS_SIGNALING_UP
-0.55941	-0.0425	-2.78285	0.006678	0.01746	-2.84057	TRIM25	c("INTERFERON_ALPHA_RESPONSE", "INTERFERON_GAMMA_RESPONSE")
0.987933	-0.10125	2.78075	0.006717	0.017555	-2.84584	CD59	c("COMPLEMENT", "EPITHELIAL_MESENCHYMAL_TRANSITION")
-0.94026	-0.11738	-2.78024	0.006727	0.017571	-2.84712	MTIF2	ALLOGRAFT_REJECTION
-1.10314	0.092908	-2.77894	0.006752	0.017627	-2.85037	PRMT3	MYC_TARGETS_V2
-0.49557	-0.0635	-2.77643	0.006799	0.017726	-2.85668	SKP1	NOTCH_SIGNALING
-0.7015	-0.04225	-2.77169	0.00689	0.017915	-2.86855	PTK2	c("ANGIOGENESIS", "APICAL_JUNCTION", "APOPTOSIS")
-1.32126	-0.64424	-2.77161	0.006892	0.017915	-2.86877	GDA	COAGULATION
-0.73406	-0.05608	-2.76957	0.006932	0.018001	-2.87386	MRPS15	OXIDATIVE_PHOSPHORYLATION
-0.70001	-0.066	-2.76949	0.006933	0.018001	-2.87407	CS	c("ADIPOGENESIS", "OXIDATIVE_PHOSPHORYLATION")
1.809076	-0.27475	2.769171	0.006939	0.018001	-2.87487	COL6A2	c("EPITHELIAL_MESENCHYMAL_TRANSITION", "MYOGENESIS")
-0.78448	-0.0945	-2.76903	0.006942	0.018001	-2.87521	SLC25A3	c("MYC_TARGETS_V1", "OXIDATIVE_PHOSPHORYLATION")
0.905846	0.082163	2.767892	0.006964	0.018032	-2.87806	ELF1	c("ESTROGEN_RESPONSE_EARLY", "INTERFERON_ALPHA_RESPONSE")

-1.25743	-0.2535	-2.76139	0.007092	0.018346	-2.89431	UQCRH	OXIDATIVE_PHOSPHORYLATION
-0.73045	-0.10513	-2.75838	0.007152	0.018474	-2.90182	BPNT1	GLYCOLYSIS
-0.68035	-0.08875	-2.75755	0.007169	0.018508	-2.9039	POLD1	c("DNA_REPAIR", "E2F_TARGETS")
-0.64414	-0.11913	-2.757	0.00718	0.018518	-2.90526	RPA2	c("DNA_REPAIR", "E2F_TARGETS", "G2M_CHECKPOINT")
1.308555	-0.76749	2.756618	0.007188	0.018529	-2.90622	CPB2	COAGULATION
-0.68723	-0.06838	-2.75581	0.007204	0.018562	-2.90824	MFN2	OXIDATIVE_PHOSPHORYLATION
0.875703	-0.37838	2.75525	0.007215	0.018577	-2.90963	ASF1A	E2F_TARGETS
-1.06314	-0.17475	-2.75401	0.00724	0.01862	-2.91271	SLC1A5	c("ADIPOGENESIS", "IL2_STAT5_SIGNALING", "MTORC1_SIGNALING", "XENOBIOTIC_METABOLISM")
-0.90596	-0.28584	-2.75217	0.007277	0.018691	-2.91731	ATP6AP1	c("MYOGENESIS", "OXIDATIVE_PHOSPHORYLATION")
0.914614	-0.29446	2.747604	0.007371	0.018865	-2.92866	GNL3	c("MYC_TARGETS_V1", "MYC_TARGETS_V2")
1.487968	-0.20235	2.745802	0.007408	0.018944	-2.93314	PIGR	KRAS_SIGNALING_UP
-0.64454	-0.10713	-2.74485	0.007427	0.018983	-2.93549	PA2G4	c("ANDROGEN_RESPONSE", "E2F_TARGETS", "MYC_TARGETS_V1", "MYC_TARGETS_V2")
-1.15352	-0.176	-2.74332	0.007459	0.019055	-2.93931	TST	c("ADIPOGENESIS", "ESTROGEN_RESPONSE_LATE", "UV_RESPONSE_UP")
0.917887	-0.30758	2.740159	0.007525	0.019214	-2.94715	ARRB2	UV_RESPONSE_UP
0.865233	-0.14338	2.733669	0.007662	0.019542	-2.96323	PGD	XENOBIOTIC_METABOLISM

-0.77129	-0.06841	-2.73356	0.007665	0.019542	-2.9635	COX7C	OXIDATIVE_PHOSPHORYLATION
-0.75159	-0.10588	-2.73168	0.007705	0.019626	-2.96817	CAD	MYC_TARGETS_V1
-0.66395	-0.09363	-2.73003	0.00774	0.019698	-2.97225	RFC3	c("DNA_REPAIR", "E2F_TARGETS")
-0.52671	-0.12813	-2.72498	0.00785	0.019957	-2.98472	TLE1	HEDGEHOG_SIGNALING
-0.48139	-0.07638	-2.71697	0.008026	0.020356	-3.00446	RPS2	MYC_TARGETS_V1
-0.80543	-0.09013	-2.70711	0.008247	0.020829	-3.02871	LMNB1	c("E2F_TARGETS", "G2M_CHECKPOINT", "MITOTIC_SPINDLE")
-0.71113	-0.116	-2.70647	0.008262	0.020847	-3.03029	TRAF2	c("ALLOGRAFT_REJECTION", "PI3K_AKT_MTOR_SIGNALING")
-0.68737	-0.02575	-2.70222	0.00836	0.021053	-3.04072	NDUFV2	OXIDATIVE_PHOSPHORYLATION
-1.04406	-0.37979	-2.69954	0.008422	0.021149	-3.0473	MAPKAP1	PI3K_AKT_MTOR_SIGNALING
-1.09291	-0.236	-2.68817	0.008689	0.021709	-3.0751	IDI1	c("ANDROGEN_RESPONSE", "BILE_ACID_METABOLISM", "CHOLESTEROL_HOMEOSTASIS", "FATTY_ACID_METABOLISM", "MTORC1_SIGNALING", "PEROXISOME")
-1.04857	-0.105	-2.68399	0.00879	0.021919	-3.0853	EGFR	c("ALLOGRAFT_REJECTION", "APICAL_JUNCTION", "GLYCOLYSIS", "HYPOXIA", "PI3K_AKT_MTOR_SIGNALING", "PROTEIN_SECRETION")
-0.69347	-0.083	-2.68288	0.008817	0.021955	-3.08801	ATP5PD	OXIDATIVE_PHOSPHORYLATION
-0.51561	-0.05263	-2.68025	0.00888	0.022073	-3.0944	PEX19	BILE_ACID_METABOLISM
-0.56495	-0.07425	-2.67556	0.008996	0.022328	-3.10583	EIF4E	c("MYC_TARGETS_V1", "PI3K_AKT_MTOR_SIGNALING",

							"UNFOLDED_PROTEIN_RESPONSE")
-0.78012	-0.85405	-2.67318	0.009054	0.022429	-3.11161	MCEE	FATTY_ACID_METABOLISM
2.12242	-1.18275	2.667606	0.009193	0.022693	-3.12514	PF4	c("ALLOGRAFT_REJECTION", "ANGIOGENESIS", "COAGULATION", "IL6_JAK_STAT3_SIGNALING")
0.540575	-6.25E-04	2.663687	0.009293	0.022863	-3.13464	SPTAN1	c("APOPTOSIS", "MITOTIC_SPINDLE", "MYOGENESIS")
0.543117	0.02	2.655686	0.009498	0.023293	-3.15401	ATP6V1D	c("MTORC1_SIGNALING", "OXIDATIVE_PHOSPHORYLATION")
-0.90081	-0.10325	-2.64784	0.009703	0.023687	-3.17295	SLC25A5	OXIDATIVE_PHOSPHORYLATION
-0.76717	-0.30521	-2.64764	0.009708	0.023687	-3.17344	MPG	DNA_REPAIR
-0.65119	-0.05563	-2.64342	0.00982	0.023911	-3.18358	ABLIM1	c("ESTROGEN_RESPONSE_EARLY", "MYOGENESIS")
-1.29346	-0.3935	-2.64341	0.00982	0.023911	-3.18361	BCAM	c("DNA_REPAIR", "HEME_METABOLISM")
-0.66399	-0.1485	-2.64229	0.009851	0.023971	-3.18632	TPI1	c("GLYCOLYSIS", "HYPOXIA", "MTORC1_SIGNALING")
-0.61457	-0.1	-2.63975	0.009919	0.024093	-3.19242	PDAP1	c("MTORC1_SIGNALING", "UV_RESPONSE_UP")
0.631773	-0.038	2.638948	0.00994	0.024134	-3.19435	WFS1	c("ESTROGEN_RESPONSE_EARLY", "ESTROGEN_RESPONSE_LATE", "UNFOLDED_PROTEIN_RESPONSE")
-0.94307	-0.19968	-2.63814	0.009962	0.024176	-3.19628	ATG4B	MITOTIC_SPINDLE
0.593113	-0.02375	2.635478	0.010034	0.024341	-3.20269	DUSP3	PI3K_AKT_MTOR_SIGNALING
0.809581	-0.22253	2.632523	0.010115	0.024492	-3.20978	RFC2	c("DNA_REPAIR", "E2F_TARGETS")

-0.608	-0.06988	-2.62815	0.010236	0.024728	-3.22026	CLASP1	MITOTIC_SPINDLE
-0.88573	0.014424	-2.62793	0.010242	0.024731	-3.22078	ZBTB10	c("ANDROGEN_RESPONSE", "TNFA_SIGNALING_VIA_NFKB")
-0.64029	-0.08475	-2.62682	0.010273	0.024772	-3.22345	RPA1	c("E2F_TARGETS", "MTORC1_SIGNALING")
-0.55771	-0.098	-2.62552	0.010309	0.024812	-3.22656	ERGIC3	PROTEIN_SECRETION
-0.84323	-0.09538	-2.62016	0.010459	0.025098	-3.23938	VDAC3	c("MYC_TARGETS_V1", "OXIDATIVE_PHOSPHORYLATION", "SPERMATOGENESIS")
-1.58778	-0.36189	-2.61621	0.010572	0.025322	-3.24882	DHCR24	c("ANDROGEN_RESPONSE", "BILE_ACID_METABOLISM", "FATTY_ACID_METABOLISM", "MTORC1_SIGNALING", "PEROXISOME")
-1.18656	-0.15969	-2.60945	0.010766	0.025744	-3.26494	METTL7B	INTERFERON_GAMMA_RESPONSE
-1.21251	-0.72266	-2.60782	0.010814	0.025843	-3.26882	ALDH1A2	KRAS_SIGNALING_UP
1.222847	-0.31213	2.603113	0.010951	0.026093	-3.28	SELENBP1	c("HEME_METABOLISM", "HYPOXIA")
-0.37166	-0.04163	-2.60308	0.010952	0.026093	-3.28009	COPB1	PROTEIN_SECRETION
-0.56941	-0.10588	-2.60188	0.010988	0.026142	-3.28292	RARS1	c("ALLOGRAFT_REJECTION", "GLYCOLYSIS")
-0.74746	-0.07238	-2.59867	0.011083	0.026309	-3.29055	HADHB	c("FATTY_ACID_METABOLISM", "OXIDATIVE_PHOSPHORYLATION")
-0.8199	-0.10063	-2.59776	0.01111	0.026352	-3.29271	GPC4	c("GLYCOLYSIS", "HYPOXIA")
1.786781	-0.3845	2.597252	0.011125	0.026376	-3.29391	BGN	c("APOPTOSIS", "EPITHELIAL_MESENCHYMAL_TRANSITION", "HYPOXIA")
-0.55993	-0.0365	-2.59158	0.011296	0.026698	-3.30737	RAD21	c("E2F_TARGETS", "G2M_CHECKPOINT")

0.544185	0.075125	2.58911	0.011371	0.026839	-3.3132	COPS5	c("MTORC1_SIGNALING", "MYC_TARGETS_V1")
-0.8432	-0.24113	-2.58826	0.011397	0.026889	-3.31521	DLAT	c("ADIPOGENESIS", "OXIDATIVE_PHOSPHORYLATION")
-0.77509	-0.08613	-2.58337	0.011547	0.027219	-3.32677	RFC5	DNA_REPAIR
-0.90321	-0.01262	-2.57748	0.011731	0.027567	-3.34067	SDAD1	UNFOLDED_PROTEIN_RESPONSE
-0.7197	-0.08463	-2.57618	0.011772	0.027638	-3.34373	ACO2	c("ADIPOGENESIS", "FATTY_ACID_METABOLISM", "OXIDATIVE_PHOSPHORYLATION", "XENOBIOTIC_METABOLISM")
-1.30646	-0.27488	-2.57547	0.011794	0.027666	-3.34541	SPON1	KRAS_SIGNALING_UP
-0.75761	-0.05075	-2.5729	0.011875	0.027832	-3.35145	CMTR1	c("INTERFERON_ALPHA_RESPONSE", "INTERFERON_GAMMA_RESPONSE")
-0.8603	-0.71524	-2.57156	0.011918	0.027895	-3.35459	SUPT4H1	DNA_REPAIR
-0.49507	-0.02575	-2.5663	0.012086	0.02824	-3.36697	TMED2	c("DNA_REPAIR", "PROTEIN_SECRETION")
0.519652	-0.021	2.56502	0.012127	0.028324	-3.36996	MIOS	UV_RESPONSE_DN
-1.35646	-0.50004	-2.56393	0.012163	0.028383	-3.37253	SLPI	KRAS_SIGNALING_UP
0.806256	-0.04838	2.563371	0.012181	0.028411	-3.37383	SDCBP	c("DNA_REPAIR", "HEME_METABOLISM")
1.761169	-0.2854	2.554347	0.012477	0.028975	-3.39497	LIPE	c("ADIPOGENESIS", "BILE_ACID_METABOLISM")
-0.74292	-0.08225	-2.55125	0.01258	0.029164	-3.40222	NDUFB4	c("OXIDATIVE_PHOSPHORYLATION", "REACTIVE_OXYGEN_SPECIES_PATHWAY")

-0.4597	-0.07525	-2.54128	0.012917	0.029841	-3.42547	KPNB1	c("G2M_CHECKPOINT", "MYC_TARGETS_V1")
-1.00057	-0.09527	-2.53543	0.013118	0.030281	-3.43907	RFC1	c("E2F_TARGETS", "MITOTIC_SPINDLE")
1.503401	-0.164	2.534487	0.013151	0.030343	-3.44126	LAMA4	ADIPOGENESIS
0.494712	-0.07363	2.529168	0.013337	0.030669	-3.45361	GMPPA	GLYCOLYSIS
-0.71802	-0.06275	-2.52913	0.013338	0.030669	-3.4537	GCDH	FATTY_ACID_METABOLISM
-0.5209	-0.10625	-2.52858	0.013358	0.030688	-3.45497	SERBP1	MYC_TARGETS_V1
-0.90385	-0.08625	-2.5248	0.013492	0.030968	-3.46372	ABCC4	ANDROGEN_RESPONSE
-0.91172	-0.17213	-2.51864	0.013713	0.031408	-3.47798	CBX1	G2M_CHECKPOINT
-0.72817	-0.07925	-2.51403	0.01388	0.031735	-3.48862	SUCLG2	FATTY_ACID_METABOLISM
1.158834	-0.53582	2.50892	0.014067	0.032083	-3.5004	PCK1	HYPOXIA
0.627949	-0.07775	2.507473	0.014121	0.032177	-3.50373	LBR	c("E2F_TARGETS", "G2M_CHECKPOINT")
0.449948	0.017	2.505255	0.014203	0.032324	-3.50883	MAPKAPK2	IL2_STAT5_SIGNALING
-0.4835	-0.08713	-2.50407	0.014248	0.032411	-3.51157	PABPC1	c("MYC_TARGETS_V1", "PEROXISOME")
-0.34521	0.01675	-2.50172	0.014335	0.032555	-3.51695	MARK2	UV_RESPONSE_UP
0.741309	2.50E-04	2.500473	0.014382	0.032648	-3.51983	PARVA	APICAL_JUNCTION

-0.42437	-0.023	-2.49683	0.01452	0.032905	-3.52819	TUBG1	c("APICAL_JUNCTION", "E2F_TARGETS", "MTORC1_SIGNALING")
1.019093	-0.48958	2.495578	0.014568	0.032957	-3.53106	STXBP1	PANCREAS_BETA_CELLS
0.907414	-0.58917	2.495314	0.014578	0.032961	-3.53166	MAPRE3	MYOGENESIS
-0.91081	-0.24463	-2.49297	0.014668	0.033116	-3.53703	ERO1A	c("GLYCOLYSIS", "HYPOXIA", "KRAS_SIGNALING_UP", "MTORC1_SIGNALING", "UNFOLDED_PROTEIN_RESPONSE")
-0.70794	-0.12025	-2.49292	0.014669	0.033116	-3.53714	GLO1	MYC_TARGETS_V1
0.908219	-0.05513	2.49115	0.014738	0.033242	-3.5412	GBP4	c("IL2_STAT5_SIGNALING", "INTERFERON_ALPHA_RESPONSE", "INTERFERON_GAMMA_RESPONSE")
0.544552	-0.02713	2.489186	0.014813	0.033371	-3.5457	VAMP3	PROTEIN_SECRETION
-0.62047	-0.09138	-2.48628	0.014926	0.033597	-3.55235	PPP4C	COMPLEMENT
-0.78805	0.011041	-2.48313	0.015049	0.033817	-3.55954	RRP12	c("ANDROGEN_RESPONSE", "ESTROGEN_RESPONSE_EARLY", "MYC_TARGETS_V2")
-0.71445	-0.06125	-2.48189	0.015098	0.033909	-3.56236	MTX2	OXIDATIVE_PHOSPHORYLATION
-0.8627	-0.14213	-2.48176	0.015103	0.033909	-3.56267	CPT2	c("ADIPOGENESIS", "FATTY_ACID_METABOLISM")
-0.71313	-0.0535	-2.47852	0.015231	0.034154	-3.57006	BCKDHA	c("ADIPOGENESIS", "OXIDATIVE_PHOSPHORYLATION")
-0.36992	-0.04238	-2.4755	0.015351	0.034403	-3.57693	COPB2	c("GLYCOLYSIS", "PROTEIN_SECRETION")
-0.37922	-0.06913	-2.4704	0.015556	0.034781	-3.58854	RPN1	c("MTORC1_SIGNALING", "UV_RESPONSE_UP")

1.212494	-0.1045	2.469915	0.015576	0.034795	-3.58963	AKAP12	c("ANDROGEN_RESPONSE", "HYPOXIA", "KRAS_SIGNALING_UP")
-0.49064	-0.06463	-2.46953	0.015592	0.034815	-3.59051	XPO7	HEME_METABOLISM
-0.76194	-0.10213	-2.46863	0.015628	0.034882	-3.59255	ETFB	c("ADIPOGENESIS", "ESTROGEN_RESPONSE_LATE", "OXIDATIVE_PHOSPHORYLATION")
-0.78957	-0.07025	-2.46791	0.015657	0.034933	-3.5942	SPR	UV_RESPONSE_UP
-0.64506	-0.06363	-2.46763	0.015669	0.034943	-3.59482	EIF2AK2	c("INFLAMMATORY_RESPONSE", "INTERFERON_ALPHA_RESPONSE", "INTERFERON_GAMMA_RESPONSE")
-0.80693	-0.12713	-2.45546	0.016171	0.035825	-3.62241	ETFA	OXIDATIVE_PHOSPHORYLATION
-0.91765	-0.08653	-2.45412	0.016227	0.035919	-3.62543	GALNT3	KRAS_SIGNALING_UP
-0.58654	-0.07625	-2.44876	0.016453	0.036344	-3.63752	PSME3	MTORC1_SIGNALING
-0.89468	-0.2505	-2.44774	0.016497	0.036425	-3.63982	PFKP	c("GLYCOLYSIS", "HYPOXIA", "INTERFERON_GAMMA_RESPONSE", "REACTIVE_OXYGEN_SPECIES_PATHWAY")
0.459997	-0.0065	2.447166	0.016521	0.036464	-3.64112	PSMB2	c("INTERFERON_GAMMA_RESPONSE", "MYC_TARGETS_V1")
1.301066	-0.071	2.446892	0.016533	0.036475	-3.64174	CAVIN3	HYPOXIA
1.034924	-0.22937	2.445234	0.016604	0.036601	-3.64548	ACOX3	XENOBIOTIC_METABOLISM
-1.65005	-0.84347	-2.43867	0.016887	0.037164	-3.66024	HPGD	c("ANDROGEN_RESPONSE", "FATTY_ACID_METABOLISM")
1.247221	-0.23136	2.43814	0.01691	0.0372	-3.66144	PCLO	COMPLEMENT

-1.06958	-0.18904	-2.43785	0.016923	0.037212	-3.66208	TRIM29	ESTROGEN_RESPONSE_LATE
-1.02687	-0.27175	-2.43274	0.017147	0.037628	-3.67356	VDAC2	c("APOPTOSIS", "OXIDATIVE_PHOSPHORYLATION")
-0.9809	-0.26075	-2.43229	0.017167	0.037656	-3.67457	ITPA	DNA_REPAIR
-0.94731	-0.64461	-2.43183	0.017187	0.037685	-3.6756	CDKN2A	c("ALLOGRAFT_REJECTION", "E2F_TARGETS", "P53_PATHWAY")
-0.78199	-0.14758	-2.4314	0.017206	0.037698	-3.67658	CELSR1	c("ESTROGEN_RESPONSE_EARLY", "HEDGEHOG_SIGNALING")
-0.54884	0.055636	-2.43137	0.017207	0.037698	-3.67663	COX7A2L	OXIDATIVE_PHOSPHORYLATION
-0.50531	-0.04275	-2.42984	0.017275	0.037801	-3.68006	NECTIN2	APICAL_JUNCTION
-0.69189	-0.107	-2.42892	0.017316	0.037874	-3.68212	ILVBL	HYPOXIA
2.575542	-0.77924	2.425463	0.017471	0.038134	-3.68986	CR2	COMPLEMENT
-0.90823	-0.51602	-2.42029	0.017704	0.038596	-3.70142	DYRK1A	UV_RESPONSE_DN
-0.68428	-0.09988	-2.41655	0.017874	0.038907	-3.70977	CASP6	c("APOPTOSIS", "GLYCOLYSIS", "HYPOXIA", "XENOBIOTIC_METABOLISM")
0.822771	-0.10213	2.415687	0.017914	0.038974	-3.7117	TPM2	c("EPITHELIAL_MESENCHYMAL_TRANSITION", "MYOGENESIS")
-0.95457	-0.21513	-2.41346	0.018016	0.039165	-3.71666	ATP1B1	OXIDATIVE_PHOSPHORYLATION
-0.40868	-0.08238	-2.41277	0.018048	0.039219	-3.7182	RPS5	MYC_TARGETS_V1
0.683205	-0.13314	2.410634	0.018147	0.039403	-3.72295	MAP4K2	APICAL_JUNCTION

-0.38089	0.014375	-2.40915	0.018216	0.039535	-3.72625	CNOT9	E2F_TARGETS
1.11712	-0.12075	2.404425	0.018437	0.039966	-3.73675	OLFML3	ESTROGEN_RESPONSE_EARLY
0.69111	-0.11175	2.402188	0.018543	0.040178	-3.74172	CTSL	COMPLEMENT
-0.84921	-0.15275	-2.40104	0.018597	0.040279	-3.74427	MYH10	c("APICAL_JUNCTION", "MITOTIC_SPINDLE")
1.480442	-0.34431	2.396291	0.018824	0.040671	-3.75479	GPNMB	KRAS_SIGNALING_UP
-0.60845	-0.11225	-2.3929	0.018986	0.040941	-3.76228	IMPDH2	c("DNA_REPAIR", "MYC_TARGETS_V1")
-1.19186	-0.3675	-2.38695	0.019276	0.041498	-3.77544	GLDC	CHOLESTEROL_HOMEOSTASIS
-0.332	-0.03775	-2.38613	0.019316	0.041551	-3.77724	COPA	EPITHELIAL_MESENCHYMAL_TRANSITION
0.912028	0.035652	2.38596	0.019324	0.041552	-3.77761	PROS1	c("COAGULATION", "XENOBIOTIC_METABOLISM")
1.370561	-0.14375	2.379784	0.01963	0.042106	-3.79122	COL4A1	c("ADIPOGENESIS", "EPITHELIAL_MESENCHYMAL_TRANSITION")
-0.57067	-0.128	-2.37946	0.019646	0.042124	-3.79194	CACYBP	MTORC1_SIGNALING
1.597644	-0.38988	2.377803	0.019728	0.042236	-3.79558	PTGIS	MYOGENESIS
1.489786	-0.13056	2.377776	0.01973	0.042236	-3.79564	ITGA5	c("EPITHELIAL_MESENCHYMAL_TRANSITION", "INFLAMMATORY_RESPONSE")
-0.45547	-0.05213	-2.37747	0.019745	0.042252	-3.79632	IDE	c("PEROXISOME", "SPERMATOGENESIS")
-0.65324	-0.0865	-2.37426	0.019906	0.042528	-3.80336	DECR1	c("ADIPOGENESIS", "FATTY_ACID_METABOLISM", "OXIDATIVE_PHOSPHORYLATION")

-0.67852	0.016	-2.3715	0.020045	0.042775	-3.80942	IDH3G	c("ADIPOGENESIS", "FATTY_ACID_METABOLISM", "OXIDATIVE_PHOSPHORYLATION")
0.477441	0.04775	2.36933	0.020156	0.042993	-3.81418	ADD3	c("ESTROGEN_RESPONSE_EARLY", "ESTROGEN_RESPONSE_LATE", "MTORC1_SIGNALING", "UV_RESPONSE_DN")
-0.64252	-0.13136	-2.36872	0.020187	0.043042	-3.81552	AVL9	KRAS_SIGNALING_UP
-0.96675	-0.18533	-2.36611	0.02032	0.043275	-3.82123	MID1	MITOTIC_SPINDLE
-0.81618	-0.18225	-2.36557	0.020348	0.043317	-3.82242	PAICS	E2F_TARGETS
-0.56174	0.045125	-2.36216	0.020524	0.043552	-3.82988	SLC25A11	OXIDATIVE_PHOSPHORYLATION
-0.76133	-0.19338	-2.36034	0.020618	0.043735	-3.83385	SLC12A2	c("G2M_CHECKPOINT", "SPERMATOGENESIS")
-0.81119	-0.10775	-2.3567	0.020808	0.044103	-3.8418	UQCR10	c("ADIPOGENESIS", "OXIDATIVE_PHOSPHORYLATION")
0.572724	-0.10863	2.354392	0.02093	0.04429	-3.84684	WASF2	MITOTIC_SPINDLE
-0.63122	-0.04913	-2.35144	0.021085	0.044567	-3.85327	HADHA	OXIDATIVE_PHOSPHORYLATION
-1.58454	-0.5445	-2.35115	0.021101	0.044574	-3.8539	CDH2	EPITHELIAL_MESENCHYMAL_TRANSITION
-0.64318	-0.04275	-2.35106	0.021106	0.044574	-3.8541	SMS	c("ANDROGEN_RESPONSE", "FATTY_ACID_METABOLISM")
-0.85446	-0.272	-2.34821	0.021258	0.044842	-3.86031	S100A13	c("COAGULATION", "COMPLEMENT")
0.550558	-0.06313	2.343822	0.021493	0.045284	-3.86984	PFN1	c("APICAL_JUNCTION", "COMPLEMENT", "PI3K_AKT_MTOR_SIGNALING")

-0.66157	0.172023	-2.33818	0.021799	0.045893	-3.88208	PPOX	HEME_METABOLISM
-0.61302	-0.09775	-2.33181	0.022149	0.046576	-3.89587	SCAF4	REACTIVE_OXYGEN_SPECIES_PATHWAY
-1.02383	-0.20488	-2.33119	0.022184	0.046611	-3.89721	PTGR1	XENOBIOTIC_METABOLISM
-0.70509	0.0373	-2.32578	0.022485	0.047208	-3.90888	NDUFAF4	MYC_TARGETS_V2
-0.44354	-0.12395	-2.32486	0.022537	0.047298	-3.91088	NXT1	c("ESTROGEN_RESPONSE_EARLY", "ESTROGEN_RESPONSE_LATE")
-0.83718	-0.1235	-2.32367	0.022604	0.04742	-3.91343	KPNA2	c("E2F_TARGETS", "G2M_CHECKPOINT", "MYC_TARGETS_V1")
-0.86144	-0.24425	-2.32119	0.022744	0.04762	-3.91877	PCNA	c("DNA_REPAIR", "E2F_TARGETS", "MYC_TARGETS_V1", "P53_PATHWAY")
0.846713	-0.16088	2.317532	0.022953	0.047981	-3.92665	PDLIM1	REACTIVE_OXYGEN_SPECIES_PATHWAY
-0.52256	-0.10888	-2.31726	0.022968	0.047995	-3.92723	FARSA	MYC_TARGETS_V2
0.449893	-0.05863	2.316355	0.02302	0.048066	-3.92918	PSMA3	c("INTERFERON_ALPHA_RESPONSE", "INTERFERON_GAMMA_RESPONSE", "MTORC1_SIGNALING")
-0.9981	-0.23975	-2.31302	0.023212	0.048429	-3.93635	GSTK1	c("BILE_ACID_METABOLISM", "PEROXISOME")
-0.68718	-0.06714	-2.30867	0.023464	0.04884	-3.94568	MAP2K6	PI3K_AKT_MTOR_SIGNALING
1.015687	-0.3747	2.306276	0.023604	0.049074	-3.9508	SLC1A4	c("ESTROGEN_RESPONSE_EARLY", "ESTROGEN_RESPONSE_LATE", "MTORC1_SIGNALING", "UNFOLDED_PROTEIN_RESPONSE")
0.575451	-0.07918	2.305677	0.023639	0.049128	-3.95208	HSPA13	UV_RESPONSE_UP

0.57295	0.01375	2.303742	0.023753	0.049307	-3.95623	MGAT1	UV_RESPONSE_UP
-0.79976	-0.11625	-2.30183	0.023866	0.049456	-3.96032	ALDH6A1	c("HEME_METABOLISM", "OXIDATIVE_PHOSPHORYLATION")
0.794126	-0.26418	2.300429	0.023949	0.049578	-3.96331	NDUFS4	OXIDATIVE_PHOSPHORYLATION
-0.88878	-0.5029	-2.2968	0.024165	0.049948	-3.97105	IRAK1	P53_PATHWAY

Supplementary Table 3. The quantities of CD8+ and CD4+ TILs in the stroma and epithelium of samples

CD4_iTIL	CD4_sTIL	CD8_iTIL	CD8_sTIL	MRN	Protypia.ID	sample_base
545.4284	726.7984	1333.836	1102.778	843767	5	OVARY
5198.6	932.7227	3115.855	1413.952	843767	51	LYMPHOID
167.8639	495.9438	734.7821	1319.347	1240597	53	OMENTUM
188.0439	533.0047	579.4002	920.8442	1240597	31	LYMPHOID
133.8823	301.527	448.8971	628.165	1240597		PERITONEUM
144.9043	242.58	199.947	380.5304	1240597	7	OVARY
282.3024	231.6042	1199.305	345.544	1240597	53	OMENTUM
3215.639	744.3042	5241.198	838.8472	1240597	72	LYMPHOID
116.5131	61.07889	728.0715	567.9344	1241142	54	OMENTUM
73.29578	278.8281	158.5912	644.2508	1241142	32	LYMPHOID
147.4979	223.2084	516.1382	353.9549	1241142	8	OVARY
105.1223	195.6607	74.67685	217.118	1117620	11	OVARY
63.2394	149.0581	54.2753	93.03847	1117620	35	BOWEL
377.464	419.82	73.80282	138.3919	1241653	4	OVARY
461.1998	473.8	604.9617	711.962	1241653	50	OMENTUM

1618.29	1307.201	3128.11	2128.4	1241653	71	LYMPHOID
224.8483	143.0538	481.7658	507.0839	1232460	9	OVARY
84.81112	117.8129	83.15691	1270.928	1232460	55	OMENTUM
3988.394	2314.184	5284.012	2987.609	1232460	33	PERITONEUM
1197.732	1122.075	855.8588	1085.27			OVARY
3994.163	3453.241	2816.211	3285.872			OMENTUM

Supplementary Table 4. The number of desert, excluded, and infiltrated regions in omental and ovarian samples for CD4+ and CD8+ TILs.

Type	Ovary CD4	Omentum CD4	Ovary CD8	Omentum CD8	patient_no	MRN	Patient ID
Infiltrated	6	25	1	4	P4	1232460	1232460
excluded	5	16	5	11	P4	1232460	1232460
Desert	8	34	5	26	P4	1232460	1232460
Infiltrated	2	5	7	10	P5		3421487
excluded	2	12	4	47	P5		3421487
Desert	4	10	4	6	P5		3421487
Infiltrated	15	4	3	26	P15	1240597	1240597
excluded	7	5	3	32	P15	1240597	1240597

Desert	7	12	3	4	P15	1240597	1240597
Infiltrated	5	6	11	5	P16	1241142	1241142
excluded	4	6	3	14	P16	1241142	1241142
Desert	20	17	16	15	P16	1241142	1241142
Infiltrated	20	3	7	3	P21	1241653	1241653
excluded	21	3	11	2	P21	1241653	1241653
Desert	22	3	17	1	P21	1241653	1241653
